# Network Pharmacology Analysis of the Therapeutic Mechanisms Underlying Beimu-Gualou Formula Activity against Bronchiectasis with *In Silico* Molecular Docking Validation

**DOI:** 10.1155/2021/3656272

**Published:** 2021-01-05

**Authors:** Xin Shen, Hong Li, Wen-Jun Zou, Jian-Ming Wu, Long Wang, Wei Wang, Hui Chen, Ling-Li Zhou, Yuan-Hui Hu, Xu-Hua Qin, Jing Yang

**Affiliations:** ^1^School of Pharmacy, Chengdu University of Traditional Chinese Medicine, Chengdu 610000, China; ^2^School of Pharmacy, Southwest Medical University, Luzhou 646000, China

## Abstract

**Background:**

The classical Chinese herbal prescription Beimu-Gualou formula (BMGLF) has been diffusely applied to the treatment of respiratory diseases, including bronchiectasis. Although concerning bronchiectasis the effects and mechanisms of action of the BMGLF constituents have been partially elucidated, it remains to be determined how the formula in its entirety exerts therapeutic effects.

**Methods:**

In this study, the multitarget mechanisms of BMGLF against bronchiectasis were predicted with network pharmacology analysis. Using prepared data, a drug-target interaction network was established and subsequently the core therapeutic targets of BMGLF were identified. Furthermore, the biological function and pathway enrichment of potential targets were analyzed to evaluate the therapeutic effects and pivotal signaling pathways of BMGLF. Finally, virtual molecular docking was performed to assess the affinities of compounds for the candidate targets.

**Results:**

The therapeutic action of BMGLF against bronchiectasis involves 18 core target proteins, including the aforementioned candidates (i.e., ALB, ICAM1, IL10, and MAPK1), which are assumed to be related to biological processes such as drug response, cellular response to lipopolysaccharide, immune response, and positive regulation of NF-*κ*B activity in bronchiectasis. Among the top 20 signaling pathways identified, mechanisms of action appear to be primarily related to Chagas disease, allograft rejection, hepatitis B, and inflammatory bowel disease.

**Conclusion:**

In summary, using a network pharmacology approach, we initially predicted the complex regulatory profile of BMGLF against bronchiectasis in which multilink suppression of immune/inflammatory responses plays an essential role. These results may provide a basis for novel pharmacotherapeutic approaches for bronchiectasis.

## 1. Introduction

Bronchiectasis is a common respiratory disease associated with irreversible dilations of the bronchi and bronchioles, due to recurrent respiratory infection, purulent inflammation, and fibrosis, and is typically manifested by chronic coughing, copious purulent sputum, and repeated hemoptysis [1]. The irreparable damage can be attributed primarily to the structural destruction of bronchial smooth muscles and elastic tissues, which gives rise to topical or widespread permanent expansion. As a common outcome of various heterogeneous diseases, including chronic obstructive pulmonary disease and bronchial asthma, bronchiectasis is characterized by a complicated etiology and pathological process [2–4]. Moreover, the recrudescent and aggravated symptoms of this disease can lead to a marked decline in the quality of life and deterioration in health.

According to the recommendations of the European Respiratory Society guidelines for the management of adult bronchiectasis [5], treatments that may be selected as appropriate, depending on the circumstances, include the use of antibiotics, anti-inflammatories, bronchodilators, and mucosal active drugs; surgical treatment; and respiratory physiotherapy. However, given that lower respiratory tract infection is a common pathogenic factor of bronchiectasis in children and adults, therapy continues to focus primarily on the use of inhibitory antibiotics [6, 7]. Nevertheless, adverse reactions to these drugs cannot be neglected and long-term antibiotic treatment can lead to the emergence and colonization of drug-resistant bacteria, with the potential of subsequent superinfection in severe cases [8]. Therefore, in-depth large-scale prospective studies are necessary to evaluate the type, dose, and duration of antibiotic use. This will not only provide important insights regarding the anti-infective therapy for bronchiectasis but will also contribute to the discovery of new therapeutic targets and drugs.

As a classical prescription in traditional Chinese medicine (TCM), Beimu-Gualou formula (BMGLF) has been applied to the treatment of pulmonary disease for more than 200 years and was first recorded in Yi Xue Xin Wu, a Chinese medical book written in the Qing dynasty by Guocheng Peng. This prescription consists of six herbs, namely, *Fritillaria Cirrhosa* (*F. Cirrhosa*, Chuanbeimu), *Fructus Trichosanthis* (*F. Trichosanthis*, Gualou), *Radix Trichosanthis* (*R. Trichosanthis*, Tianhuafen), *Poria Cocos* (*P. Cocos*, Fuling), *Exocarpium Citri Grandis* (*E. C. Grandis*, Huajuhong), and *Platycodon Grandiflora* (*P. Grandiflora*, Jiegeng). In the theory of traditional Chinese medicine, the efficacies of BMGLF are described as clearing the lungs, moistening dryness, regulating Qi activity, and eliminating phlegm. On the basis of *in vivo* and *in vitro* studies, these conventional efficacies are gradually being translated into current pharmacological terms, namely, anti-infective [9, 10], anti-inflammatory [11–15], spasmolytic [16], anti-asthmatic, and expectorant effects [17–19].

Actually, BMGLF has shown promise for treating bronchiectasis because of formula-syndrome correspondence in TCM and its active components. In TCM theory, the manifestations of bronchiectasis are considered to fall into the syndrome of phlegm-heat obstructing lung [20, 21]. Hence, the therapeutic principle of clearing lung and eliminating phlegm in TCM is required for bronchiectasis relevant to that syndrome. In this regard, BMGLF and its modified/derivative formulae (Qing-Jin-Hua-Tan decoction for instance) have been widely used in bronchiectasis treatment owing to their common efficacies of clearing lung and eliminating phlegm [22, 23]. Moreover, the results of current pharmacological research have suggested the anti-inflammatory and expectorant activities play vital roles in BMGLF-induced bronchiectasis remission. Therefore, it is easy to understand the feasibility of BMGLF treating bronchiectasis.

**Figure 1:**
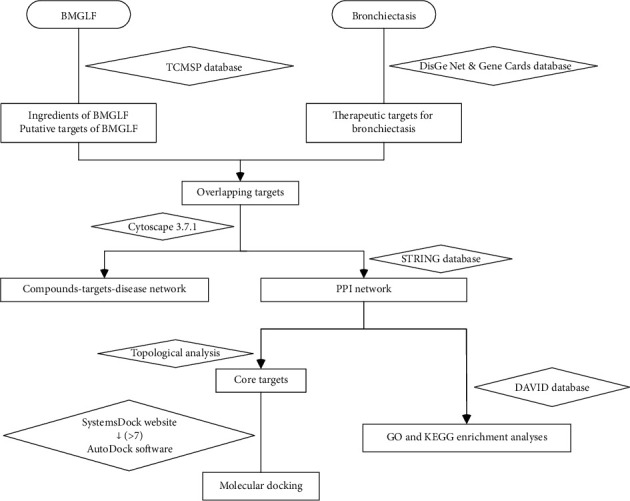
Flowchart of network pharmacology analysis and molecular docking verification of the mechanisms underlying Beimu-Gualou formula (BMGLF) activity against bronchiectasis.

Although it is evident that certain ingredients of BMGLF play crucial roles in the treatment of bronchiectasis, by simply focusing on single targets, we fail to fully comprehend the complex nature of the therapeutic mechanisms of this preparation. In this aspect, the emerging discipline of network pharmacology analysis opens up a window to comprehensively assess the comprehensive mechanisms of action of traditional Chinese medicine prescriptions. Using this approach can help elucidate the relationships among multiconstituent agents, multiple targets, and complex disease by constructing compound-protein and protein-protein interaction networks, thereby facilitating the transition from monotarget to multitarget research in an effective and reliable manner. In this study, we screened the components of BMGLF that show efficacy against bronchiectasis and predicted the potential targets and pathways via network pharmacology analysis. In addition, by way of verification of the predicted mechanisms, we examined the interactions between candidate drug molecules and target proteins by performing virtual docking analysis. On the basis of our findings, we provide guidance relating to the experimental verification of mechanisms and anticipate the future development of multitarget-modulated agents for bronchiectasis treatment. The technical procedures undertaken in this study are presented as a flowchart (Figure 1).

## 2. Materials and Methods

### 2.1. Chemical Constituents and Targets of BMGLF

For data preparation, we searched all the constituent herbs in BMGLF (*F. Cirrhosa*, *F. Trichosanthis*, *R. Trichosanthis*, *P. Cocos*, *E. C. Grandis*, and *P. Grandiflora*) within the Traditional Chinese Medicine Systems Pharmacology Database and Analysis Platform (TCMSP, http://lsp.nwu.edu.cn/tcmsp.php) [24]. Following data summarization, we obtained lists of all the chemical constituents and their corresponding targets, the latter of which were converted to standard gene names via the UniProt database (https://www.uniprot.org/) [25].The duplicated, nonhuman, and nonstandard targets were eliminated.

### 2.2. Screening for Bronchiectasis-Related Targets

To assemble targets associated with bronchiectasis, we searched the DisGeNET (http://www.disgenet.org/) and GeneCards (https://www.genecards.org/) databases [26, 27] using “Bronchiectasis” as a keyword, and after merging, we deleted duplicates to establish a final disease target dataset.

### 2.3. Construction of an Ingredient-Target Network

After collating drug and disease targets, these two target categories were compared and filtered to identify common elements. The potential targets of BMGLF related to the treatment of bronchiectasis were then input into Cytoscape 3.7.1 software for construction of a chemical ingredient-target network [28].

### 2.4. Network Topology Analysis of Protein-Protein Interaction

It is known that intracellular biochemical processes often involve interactions among multiple coparticipating proteins. Owing to emphasis on multitargeted adjustment of a Chinese herbal prescription, we used protein-protein interaction (PPI) networks to characterize the therapeutic mechanisms of BMGLF against bronchiectasis. For acquirement of PPI data, we input the common target information into the STRING database (https://string-db.org/) [29]. Data were analyzed under conditions in which the species was set as “*Homo sapiens*” and the confidence score was defined as >0.4, and the data were visualized in Cytoscape 3.7.1. We opted to define the size and color of nodes and the thickness of edges to separately reflect the magnitude of degree and the level of combination score. On the basis of the PPI network thus established, we employed Network Analyzer [30], a Cytoscape plugin, to perform topology analysis on nodes, using three topological indicators (degree, betweenness, and closeness) as screening criteria to evaluate the essentiality of each node within the entire network and to confirm core targets.

### 2.5. Target Function and Pathway Enrichment Analyses

We used the Database for Annotation, Visualization, and Integrated Discovery (DAVID, https://david.ncifcrf.gov/) to perform Gene Ontology (GO) functional enrichment analysis on the common targets in the PPI network [31]. The three functional categories cell component, molecular function, and biological process were separately assessed to identify target protein functions. In addition, we also carried out pathway enrichment analysis of these targets using the Kyoto Encyclopedia of Genes and Genomes (KEGG) database [32].OmicShare (http://www.omicshare.com/) [33], a free online data analysis platform, was used to visualize the main action pathways.

### 2.6. Docking Score Acquisition Using SystemsDock

The SystemsDock website (http://systemsdock.unit.oist.jp/iddp/home/index) is a web server dedicated to the prediction and analysis of network pharmacology [34] and capable of validating whether a small-molecule compound binds tightly to a protein from the perspective of the interaction parameter. After initially submitting the PDB IDs of potential key molecules, we extracted 3D conformations of the components from the PubChem database (https://pubchem.ncbi.nlm.nih.gov/) [35]. The latter were uploaded to the website for docking. Thereafter, we identified key proteins and small molecules from the principle that a docking score greater than 7 is indicative of strong binding activity. The interacting proteins and small molecules with docking scores greater than 7 were used for further docking verificationvia AutoDock 4.2 [36].

### 2.7. Docking Image Collection Using AutoDock 4.2

AutoDock 4.2 is a type of open-source molecular simulation software that can be used to graphically depict the interactive pattern between a ligand (small molecule) and a receptor (protein). Before performing docking analysis, we prepared structure files of macromolecular proteins and micromolecular ligands. The crystal structures of proteins were successively subjected to dehydration, hydrogenation, and removal of ligands from the RCSB database (http://www.rcsb.org/) [37]. Other docking and scoring parameters were applied using default settings. The optimal conformation was ascertained dependent on the codeterminants of free energy and inhibition constant and then embellished by using PyMOL software [38].

## 3. Results

### 3.1. Details of BMGLF Compounds and Targets

Using the TCMSP database, we identified a total of 319 chemical compounds in BMGLF: 63 compounds in *F. Cirrhosa*, 80 in *F. Trichosanthis*, 15 in *R. Trichosanthis*, 34 in *P. Cocos*, 44 in *E. C. Grandis*, and 102 in *P. Grandiflora* (Supplementary Table 1). Following subsequent searches in the TCMSP database and transformation via the UniProt database, we obtained the gene names of 385 drug-associated targets, with no repetition (Supplementary Table 2).

### 3.2. Construction and Comparative Analysis of Target Data Related to Bronchiectasis

**Figure 2:**
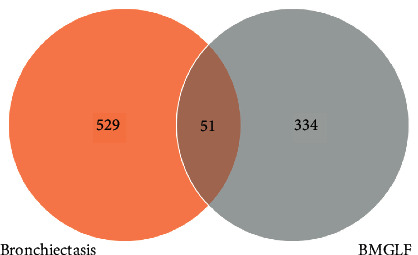
Venn diagram of disease and drug targets. Fifty-one common targets were identified as potential therapeutic targets for the BMGLF treatment of bronchiectasis.

On the basis of searches of the DisGeNET and GeneCards databases, we identified 111 and 569 targets relevant to bronchiectasis, respectively. After repeat data elimination, the remaining 580 targets were used to construct a pathological target database (Supplementary Table 2). By mapping drug targets to disease targets, we identified 51 common targets (Figure 2), including IL10, TLR2, STAT3, IL6, and CXCL8, which served to indicate putative pathways whereby BMGLF could be used to treat bronchiectasis.

### 3.3. Putative Ingredient-Target Network

**Figure 3:**
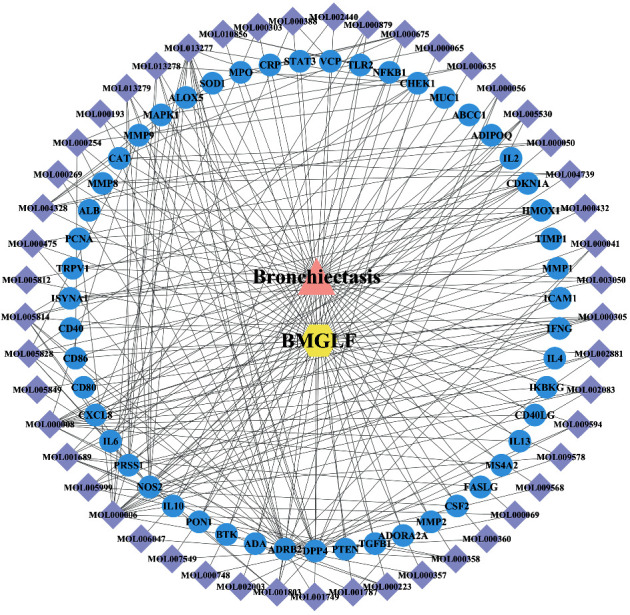
Compound-target-disease network. The triangular pink node represents the disease; the hexagonal yellow node represents the TCM formula; the circular blue nodes represent the common targets; and the rhomboidal purple nodes represent the chemical components of *F. Cirrhosa* (11), *F. Trichosanthis* (15), *R. Trichosanthis* (2), *P. Cocos* (1), *E. C. Grandis* (16), and *P. Grandiflora* (4).

Cytoscape 3.7.1 software was employed to visualize ingredient-target correspondence as an interactive network for bronchiectasis treatment. The network comprised 102 nodes and 252 edges (Figure 3). As shown in the information of the targets and their related compounds (Table 1), most paired compounds, including *β*-sitosterol, lauric acid, and apigenin, were derived from *F. Cirrhosa*, *F. Trichosanthis*, and *E. C. Grandis*. These compounds were found to be closely associated with the most viable targets for treating bronchiectasis.

### 3.4. PPI Network Construction and Target Screening

**Figure 4:**
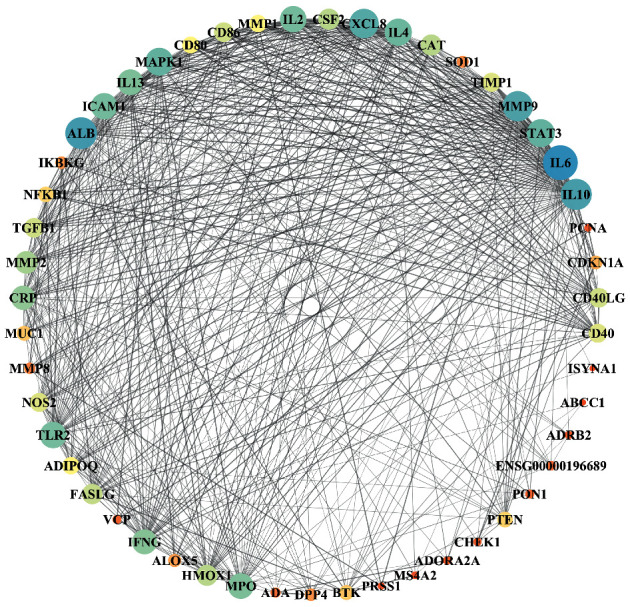
Protein-protein interaction (PPI) network for the activity of BMGLF against bronchiectasis. Eighteen targets that occupy important positions in the network as core targets were identified.

In order to depict an interactive network among targets (Figure 4), we initially entered the common targets into the STRING database and imported the information of node1, node2, and the binding score in the result files into Cytoscape 3.7.1. The resultant network contained 51 nodes and 538 edges, which denoted proteins and the interrelations among proteins, respectively. The larger and darker the node, the larger the corresponding degree value. Similarly, the thicker the edge, the greater the value of the combination score between the two targets. Using topology attribute analysis in NetworkAnalyzer, we found that nodes with a higher betweenness and closeness tended to have a larger degree of connection. The core targets were screened under the condition of betweenness >0.00553327, closeness >0.625, and degree >23, that is, the above three parameters were set as greater than their respective medians, and we accordingly identified 18 key proteins, namely, IL6, ALB, IL10, MMP9, CXCL8, MAPK1, STAT3, IL4, ICAM1, IL2, TLR2, MPO, IL13, IFNG, CPR, MMP2, CAT, and HMOX1.

All the relevant information relating to these proteins is presented in Table 2. These findings clearly indicate the prominent contribution of inflammatory cytokines to the pathogenesis in bronchiectasis and that these could thus serve as potential therapeutic targets.

### 3.5. GO Functional Enrichment

**Figure 5:**
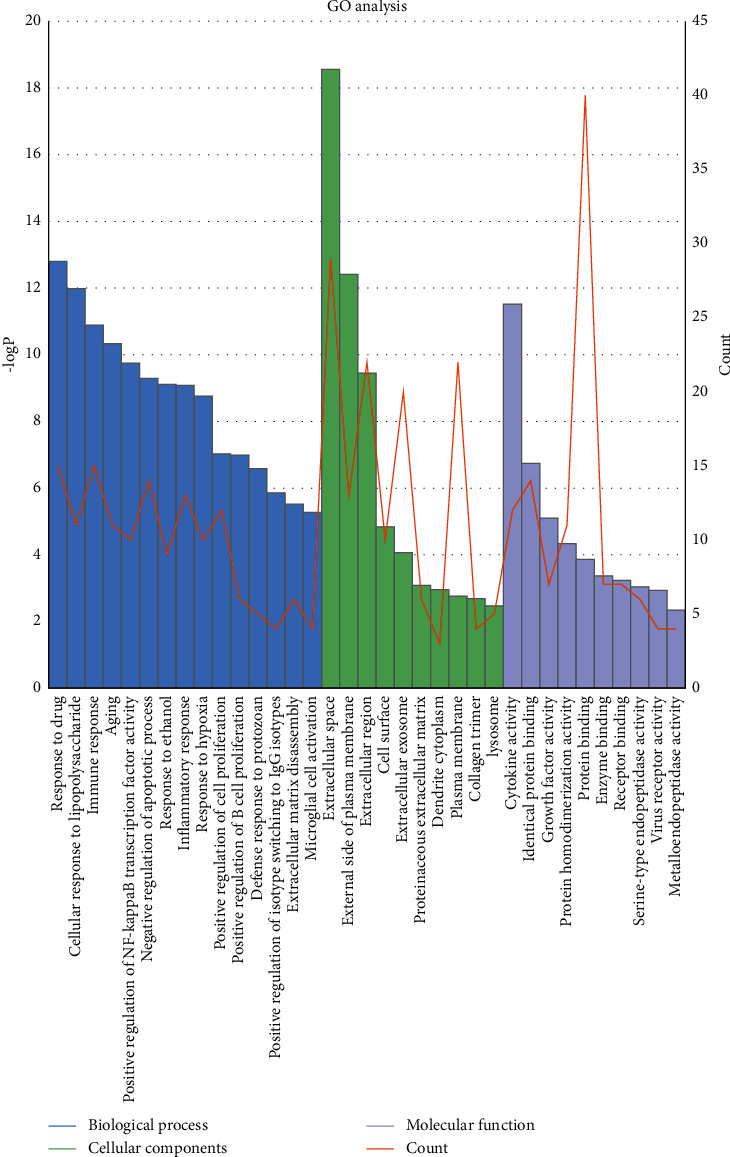
Gene Ontology (GO) enrichment based on bioinformatics data. The histogram reflects the magnitude of the (P) value, and the line chart shows the number of genes.

GO functional enrichment analysis on the 51 potential targets in the PPI network was performed using the DAVID database. Under a significance threshold of *P* < 0.05, we identified 212 GO entries, of which 173 were related to biological processes, including response to drug, cellular response to lipopolysaccharide, immune response, inflammatory response, aging, positive regulation of NF-kappa B transcription factor activity, and negative regulation of apoptotic process. Further 25 entries were related to cellular components, including extracellular space, external side of plasma membrane, extracellular region, extracellular exosome, and cell surface, whereas the remaining 14 were related to molecular functions, including cytokine activity, protein binding, and protein homodimerization activity (Figure 5 and Supplementary Table 3).

### 3.6. KEGG Pathway Enrichment

**Figure 6:**
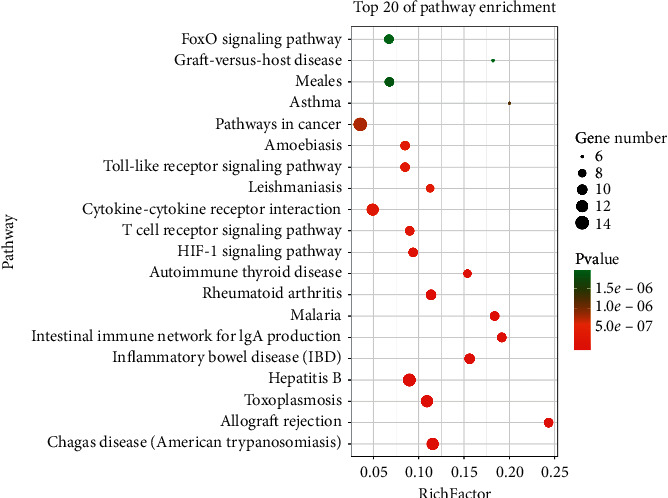
Pathways involving predicted potential therapeutic targets. The signaling pathways associated with the action of BMGLF against bronchiectasis were found to be closely associated with immunity and inflammation.

In addition to functional assignments, we also identified 62 significantly enriched (*P* < 0.05) signaling pathways obtained by KEGG analysis using the DAVID database (Supplementary Table 4). The top 20 pathways most significantly associated with disease were as follows: Chagas disease (American trypanosomiasis), allograft rejection, toxoplasmosis, hepatitis B, inflammatory bowel disease (IBD), intestinal immune network for IgA production, malaria, rheumatoid arthritis, autoimmune thyroid disease, HIF-1 signaling pathway, T-cell receptor signaling pathway, cytokine-cytokine receptor interaction, leishmaniasis, Toll-like receptor signaling pathway, amoebiasis, pathways in cancer, asthma, measles, graft-versus-host disease, and FoxO signaling pathway. We accordingly assume that the mechanisms whereby the constituents of BMGLF act on bronchiectasis involve at least some of these signaling pathways. A bubble map drawn by using OmicShare is shown in Figure 6 to present the results.

### 3.7. Verification of Drug-Target Combinations

Among the core targets in the PPI network, we selected the top 10 for analysis of molecular docking. Given that STAT3 and CXCL8 did not have specific binding sites and that we were unable to identify the 3D structure of the compound ZINC03860434 in the PubChem database, these targets were excluded from the docking analysis. The docking results revealed 14 combinations with a docking score greater than 7, indicating that the BMGLF constituent compounds have high affinities for the target proteins. Detailed information on these combinations relating to the chemical constituents of *F. cirrhosa*, *R. Trichosanthis*, *E. C. Grandis*, and *P. Grandiflora* is presented in Table 3. The matching core targets included ALB, IL10, ICAM1, and MAPK1, indicating that the effective ingredients of the herbs in this formula might physically interact with these targets.This result characterizes the unique pharmacodynamic properties of BMGLF with respect to the treatment of bronchiectasis.

### 3.8. Visualization of Molecular Docking Data

**Figure 7:**
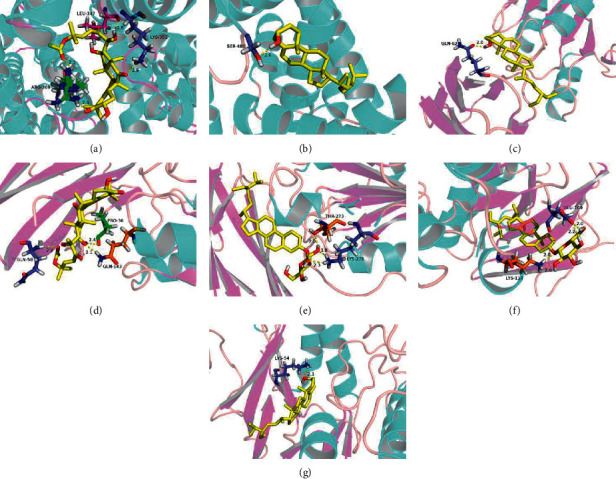
Molecular docking of potential targets and chemical components in BMGLF. (a) ALB vs. cucurbitacin b. (b) ALB vs. *β*-sitosterol. (c) ICAM1 vs. *β*-sitosterol. (d) ICAM1 vs. cucurbitacin b. (e) ICAM1 vs. sitogluside. (f) MAPK1 vs. sitogluside. (g) MAPK1 vs. *β*-sitosterol. The dotted lines denote hydrogen bonds with the corresponding bond lengths displayed above. The sticks represent interacting ligands and amino acid residues, and the proteins are displayed as secondary structures.

The data based on the systems molecular docking analysis of the 14 pairs of BMGLF compound-protein combinations were visualized by using AutoDock software. In this regard, we adopted a semiflexible docking model, *i.e.*, a rigid configuration of the protein versus a flexible configuration of small-molecule constituents. Grid energy was calculated using AutoGrid, and the docking operation was based on a Lamarck genetic algorithm. The parameters used for docking visualization are listed in Table 4. Binding free energy (FE) and inhibition constant (*K*_i_) served as indicators for binding strength. In terms of changing tendency, *K*_i_ showed a negative correlation with binding force, whereas the absolute value of FE was positively correlated with the intensity of bond characterization. We found that a vast majority of the values obtained were consistent with the results of the systems docking analysis, thereby further verifying tight combinations between the proteins and constituent compounds, as well as the reliability of the network hub nodes. To demonstrate the simulated docking more intuitively, we used *K*_i_ < 1 *μ*M threshold in PyMOL to optimize the visible combinations. In the docking models depicted in Figure 7, the compounds are marked yellow and the hydrogen bond lengths and the linked amino acid residues are indicated. As shown in Figure 7, the protein ALB shows intense binding to cucurbitacin b and *β*-sitosterol; ICAM1 combines closely with *β*-sitosterol, cucurbitacin b, and sitogluside; and MAPK1 binds tightly to sitogluside and *β*-sitosterol.

## 4. Discussion

The statistics obtained from a recent global-wide epidemiological investigation have revealed a striking increase in the incidence of bronchiectasis-related morbidity [39–41]. Accordingly, considering the acknowledged limitations of antibiotic therapy, traditional Chinese medicine may provide a more sustainable approach for the treatment of bronchiectasis. In this regard, BMGLF, a traditional Chinese medicine eutherapeutic prescription used against syndromes of cough and expectoration, has overt curative effects on respiratory diseases. However, given its complex composition and compatibility, the mechanisms of action of BMGLF with respect to bronchiectasis remain unclear. Moreover, from the perspective of reductionism, it is difficult to illustrate the sophisticated molecular pathways involved. Under these circumstances, network pharmacology-based identification provides a more holistic approach to elucidating the interconnected molecular mechanisms underlying the effects of BMGLF against bronchiectasis.

In the present study, through integrating the corresponding target data of herbal constituents and interventional target data of bronchiectasis, we identified 18 core targets via PPI network analysis. Subsequent KEGG analysis revealed that most of the relevant molecular pathways are associated with immune response or inflammation. Our results identified numerous diseases that have a bearing on both infectious and noninfectious immune activation, including Chagas disease, allograft rejection, toxoplasmosis, hepatitis B, and inflammatory bowel disease. On the basis of pathological changes, we surmise that the salient activities of BMGLF against bronchiectasis are associated with anti-inflammatory and anti-infection effects.

Since airway inflammation is a characteristic feature of bronchiectasis, the hub targets that are linked with bronchial inflammation may contribute to identifying the molecular pathways relevant to BMGLF therapy [42]. Hence, in our analyses of the remedial mechanism of BMGLF, we mainly focused on the top targets based on degree values. We found that the vast majority of these target proteins are inflammation-related factors (including inflammatory cytokines and intracellular signaling messengers) that play pivotal roles in inflammation- and/or immunoreaction-associated pathological processes. Thus, itcan be speculated that BMGLF may alleviate bronchial inflammation by modulating the levels of inflammatory factors.

The findings of previous studies have highlighted that maladjustment of the inflammatory factor network is a concern in chronic inflammatory diseases [43, 44]. Initially, neutrophils are recruited to the inflammatory locations in response to CXCL8-induced and ICAM1-mediated chemotaxis [45–48]. In addition, constitutive MAPK1 activation also lies at the nexus of CXCL8-triggered neutrophil migration via interceding CXCL8 expression [49, 50]. Through synergistic action with other enzymes, MMP9 induces the inactivation of antileukocyte protease and subsequently aggravates inflammatory tissue damage [51–53]. Characterized as a dual-function regulator, IL6 not only reinforces CD4+ T-cell response by the induced gp130/Jak2/STAT3/NF-*κ*B signaling cascade but also suppresses the secretion of TNF*α* and IL1 to attenuate inflammation [54, 55]. Moreover, IL10 has a downregulatory effect on transcription factor NF-*κ*B and thereby reduces proinflammatory cytokine expression [56, 57]. Accordingly, the interrelated activities of these inflammatory cytokines provide a theoretical basis explaining the PPI interaction network.

**Figure 8:**
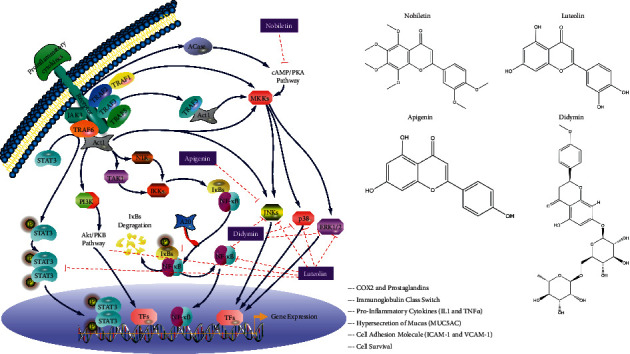
Four compounds representative of ingredients from BMGLF and target proteins of their actions. Of these compounds, didymin restrains the activation of NF-*κ*B, JNK, p38, and ERK1/2. Apigenin mainly suppresses JNK activation. Luteolin extensively downregulates the phosphorylation of inflammatory signaling molecules such as STAT3, PI3K/Akt, NF-*κ*B, JNK, p38, and ERK1/2. Nobiletin has an inhibitory impact on cAMP/PKA signaling, proceeding with the suppression of downstream MAPK signaling. It is thus clear that MAPK signaling (inclusive of JNK, p38, and ERK1/2) may serve as the common and pivotal target for anti-inflammatory treatment with BMGLF.

The herbal constituents in BMGLF represent a material foundation for targeting node proteins and treating bronchiectasis. For example, previous studies have revealed that nobiletin (derived from *E. C. Grandis*) has an anti-inflammatory activity in multiple systems of the human body, and its mechanism of action may be related to the inhibited expressions of iNOS and COX-2 [58]. It is worth mentioning that nobiletin can sensitize the cAMP/PKA-dependent pathway and proceed to activate the topmost CFTR channels in the membranes of bronchial epithelial cells to promote Cl^−^ secretion. This process then helps restore the tracheal mucus clearance through the adjustment of ion transmission [59]. Didymin (derived from *E. C. Grandis*) is a flavonoid compound that has experimentally proven to have a striking effect on restraining inflammatory response and sputum generation. Molecularly, this compound can successively inhibit NF-*κ*B signaling and ICAM-1 and VCAM-1 expressions in cell adhesion and ultimately downregulate the levels of inflammatory cytokines and chemokines. In terms of sputum production, it is able to decrease the phosphorylation of JNK, ERK, and p38 (i.e., partially inhibiting the MAPK signaling) and further downregulate MUC5AC expression to attenuate mucus hypersecretion [60, 61]. Likewise, luteolin (derived from *P. Grandiflora*) plays an anti-inflammatory role by targeting such core regulators of the host immune cascades as Src in the NF-*κ*B pathway, MAPK in the AP-1 pathway, and SOCS3 in the STAT3 pathway [62]. Both MMP9 and PI3K/AKT signaling also contribute to the potential targets of luteolin action for the treatment of ischemic stroke-related inflammation [63]. Similarly, it has been demonstrated experimentally, both *in vitro* and *in vivo*, that apigenin (derived from *E. C. Grandis*) modulates the JNK pathway and significantly inhibits the mRNA and protein expressions of ICAM1, IL6, and CXCL8 in human umbilical vein endothelial cells exposed to DEHP [64]. In fact, the anti-inflammatory effect of apigenin is primarily subject to its immunocyte-suppressive property. The latent mechanisms are likely associated with the inhibition of histamine and TNF*α* release in mast cells and basophils, the induction of neutrophils apoptosis by activating caspases, and the decrease of NO and PGE2 production in macrophage inflammation model [65]. Thus, it can be seen that the therapeutic effects of these representative ingredients principally link with suppressing inflammation-regulatory targets. The distinct targets of actions of these compounds may interconnect and play essential role in inflammation-related signaling pathways, as shown in Figure 8. It is thus clear that at least some of our analytical results relating to anti-inflammatory mechanisms are consistent with the findings of these previous studies. More uniquely, however, the network analysis performed in the present study provides evidence that certain bioactive small molecules as diverse as cucurbitacin *b*, sitogluside, and *β*-sitosterol target MAPK1 and ALB proteins. Moreover, the recorded values of bonded parameters consistently indicate the validity of the putative ingredient-target combinations; for example, cucurbitacin b-ALB binding via LEU-347 with H-bond length 0.18 nm, via LYS-351 with H-bond length 0.19 nm, and via ARG-209 with H-bond lengths 0.21 nm and 0.26 nm; sitogluside-MAPK1 binding via GLU-109 with H-bond lengths 0.2 nm and 0.22 nm and via LYS-114 with double H-bond lengths 0.2 nm; and *β*-sitosterol–MAPK1 binding via LYS-54 with H-bond length 0.21 nm. Additionally, we found the compound-ALB binding corresponds to “response to drug” in the GO functional category biological process, which suggeststhe pharmacokinetics of the components in BMGLF may be linked to the efficacy of BMGLF against bronchiectasis. In general, we speculate that the curative mechanisms of BMGLF in bronchiectasis are primarily associated with the expressional regulation of inflammatory factors such as IL6, IL10, MMP9, CXCL8, MAPK1, and ICAM1.

**Figure 9:**
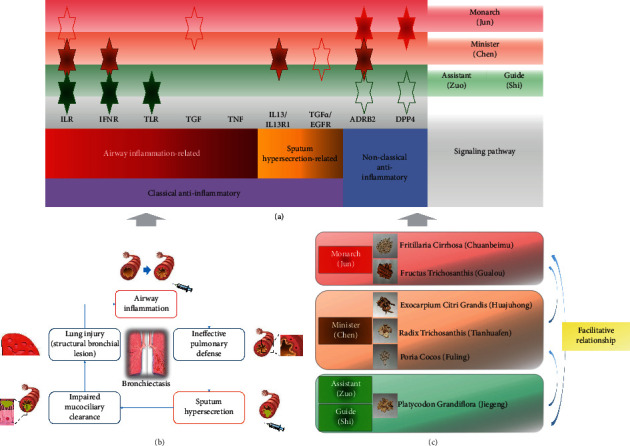
Compatibility of 6 herbs in BMGLF corresponding with pathological process and therapeutic orientation of bronchiectasis. (a) Mechanisms in therapeutic actions of BMGLF functional units. (b) Vicious circle in bronchiectasis progression. (c) Four functional units of BMGLF. The red and tawny hexagrams symbolize the Jun and Chen in BMGLF, respectively. The Zuo and Shi are both represented using the deep green hexagrams. The hollow hexagrams signify the less enriched pathways of the functional units than the filled hexagrams. ILR, interleukin receptor; IFNR, interferon receptor; TLR, Toll-like receptor; TGF, transforming growth factor; TNF, tumor necrosis factor; TFs, transcription factors.

**Figure 10:**
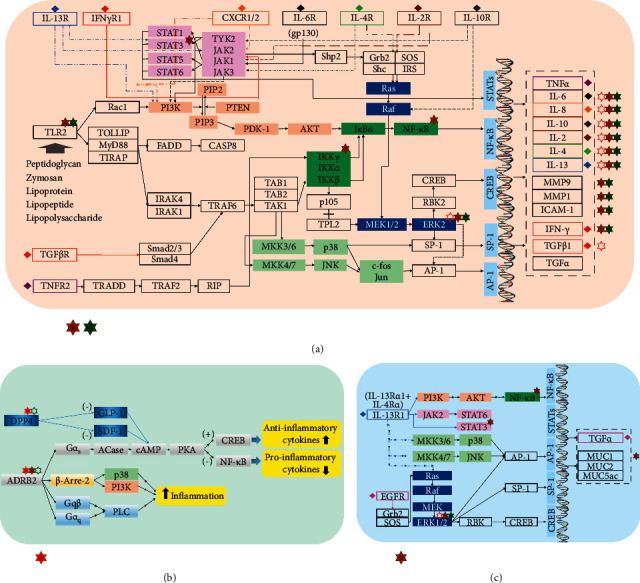
Airway inflammation and sputum hypersecretion pathways and targets of actions of functional units in BMGLF. (a) Classical inflammation-regulatory signaling pathways. (b) Nonclassical inflammation-regulatory signaling pathways. (c) Sputum hypersecretion-related signaling pathways. The arrows between nodes represent the facilitating effects. The rhombi in different colors denote the correlations between varying cytokines and their color-consistent receptors. See Figure 9 for the meanings of the hexagrams.

Besides exploring the relationship between the chemical ingredients and therapeutic targets of BMGLF, we complementally analyzed the compatibility of 6 herbs in BMGLF, relating to the pathogenetic mechanism of bronchiectasis. The primary pathological process of bronchiectasis incorporates airway inflammation, ineffective pulmonary defense, sputum hypersecretion, impaired mucociliary clearance, and lung injury (structural bronchial lesion) [66–68]. These pathological changes form a vicious circle (Figure 9(b)), worsening the symptoms of cough, purulent yellow sputum, hemoptysis, and tightness in breathing. Infection- or noninfection-caused bronchial inflammation and sputum hypersecretion greatly affect bronchiectasis progression and also act as the directions for medication treatment [69]. The bioinformatics data in our analyses manifested that 5 categories of molecular signaling pathways chiefly engaged in airway inflammation, embracing interleukin receptor (ILR) signaling, interferon receptor (IFNR) signaling, Toll-like receptor (TLR) signaling, transforming growth factor (TGF) signaling, and tumor necrosis factor (TNF) signaling (Figure 9(a)). Moreover, 2 types of molecular signaling pathways, such as IL-13/IL-13R1 and TGF*α*/EGFR signaling, bore on sputum hypersecretion (Figure 9(a)). Generally, all of these pathways pertain to the classical inflammation-regulatory signaling pathways (Figures 9(a) and 10(a)). The crosstalk of them confirms the correlation between airway inflammation and sputum hypersecretion, as embodied in JAK/STAT, PI3K/AKT, IKK/NK-*κ*B, and MAPK signaling plus their downstream transcription factors (Figure 9(a)).

From a clinical standpoint of treatment, BMGLF exerts an ameliorative influence on the cardinal symptoms in bronchiectasis patients, especially cough and purulent yellow sputum. What's more, the results in our study have proven that BMGLF centrally intervened in inflammatory reaction, the core pathological change in bronchiectasis. However, the remedial effects of the entire prescription are largely dependent on the combination of the participating herbs, which is expressed as four functional units covering Jun (Monarch drug), Chen (Minister drug), Zuo (Assistant drug), and Shi (Guide drug) in TCM (Figure 9(c)). In the prescription BMGLF, for example, the Jun contains Chuanbeimu (*F. Cirrhosa*) and Gualou (*F. Trichosanthis*), playing a principal role in relieving cough and reducing sputum against the airway syndromes. The Chen encompasses Huajuhong (*E. C. Grandis*), Tianhuafen (*R. Trichosanthis*) and Fuling (P. Cocos), which can enhance the curative action of the Jun and also remove certain secondary symptoms such as dry and sour throat, thick tongue coating, and darker urine. Jiegeng (*P. Grandiflora*) concurrently functions as the Zuo and Shi, capable of attenuating dry and sour throat, improving respiratory function, and leading the whole prescription action to lungs. Obviously, the collaboration of the Jun, Chen, Zuo, and Shi in BMGLF conducts a comprehensive therapy for bronchiectasis.

From the angle of the mechanisms of action, the Jun, Chen, Zuo, and Shi in BMGLF tend toward distinct signaling pathways according to our enrichment analyses (Figures 9(a) and 10). Nevertheless, these pathways may overlap in part or intersect each other at some specific nodes, whichis presented by the integrated data in this research. On the one hand, in terms of the airway inflammation-related molecular pathways (Figures 9(a) and 10(a)), the Jun, Chen, Zuo, and Shi collectively modulated the ILR signaling and yet the enrichment of the Jun in this pathway was inferior to that of Chen, Zuo, and Shi. The latter three also touched upon the IFNR signaling. Moreover, the TLR signaling was exposed to the Zuo and Shi. The TGF signaling was less enriched for the Jun than the other pathways for it. On the other hand, with regard to the molecular pathways in sputum hypersecretion (Figures 9(a) and 10(c)), the IL-13/IL-13R1 signaling was mainly subject to the adjustment of the Chen, while the TGF*α*/EGFR signaling was less subject to the Jun than the other pathways to it. Thus, it can be seen that the significant regulations on the mechanisms of airway inflammation are chiefly in the charge of the Chen, Zuo, and Shi. Furthermore, the Chen markedly modulates the mechanisms of sputum hypersecretion. By contrast, the molecular pathways of both airway inflammation and sputum hypersecretion do not seem preferred for the Jun. Hence, to make clear the Jun's action tendency, we analyzed the potential targets of chemical constituents from the Jun and the rest of the formula. Lastly, two molecules ADRB2 and DPP4 came into sight. In fact, the two molecules are instrumental in the pathological inflammatory processes [70–75], particularly the airway inflammation as stated by previous study reports [76, 77]. Exactly speaking, activated ADRB2 transmits an anti-inflammatory signal via the G*α*s/ACase/cAMP/PKA cascade or amplifies the inflammation through 3 signaling pathways separately concerning *β*-arrestin-2, Gq*β*, and G*α*q proteins; DPP4 unites with the substrates GLP-1 and SDF-1 for alteration of the inflammatory state (Figure 10(b)). Their terminal effects concentrate on the inflammation-associated transcription factors (Figure 9(a)). Hence, a reasonable conjecture is that ADRB2 and DPP4 may enroll in nonclassical inflammation-regulatory pathways (Figures 9(a) and 10(b)). The data suggested that ADRB2 was severally targeted by the Jun, Chen, Zuo, and Shi, whereas the influences of the Zuo and Shi on it were weaker than those of the Jun and Chen. In addition, DPP4 was linked to the Jun, Zuo, and Shi, but the impacts of the Zuo and Shi on it were weaker than that of the Jun. Thus, in summary, the Jun in BMGLF is apt to intervene in the nonclassical inflammation-regulating pathways. The Chen, Zuo, and Shi mostly influence the classical inflammation-regulating pathways. Additionally, the Chen strengthens the Jun's therapeutic effects using the coordination to the nonclassical inflammation-regulating and sputum hypersecretion-related pathways. The Zuo and Shi chiefly work on the classical inflammation-regulating pathways to reinforce the curative effects of the Jun and Chen. These presumptions validate the compatibility of the Jun, Chen, Zuo, and Shi in BMGLF.

## 5. Conclusion

Now widely acknowledged as valuable ethnic medicines, traditional Chinese medicine formulae represent novel treatment options for a range of complex diseases. In this study, we adopted a systematic approach to comprehensively analyze the pharmacological mechanisms underlying the efficacy of BMGLF in the treatment of bronchiectasis. Furthermore, we assessed the various contributions of chemical components and anti-inflammatory pathways based on drug-target and protein–protein interaction networks, which enabled us to predict the latent or pivotal targets for therapeutic intervention. Although further experimental validations will be necessary to confirm our predictions and clarify the mechanisms underlying the effects of BMGLF against bronchiectasis, our current findings provide new insights into the range of selectable therapeutic strategies for bronchiectasis.

## Figures and Tables

**Table 1:** Information of the common targets and related compounds in BMGLF.

ID	Compound	OB (%)	DL	Gene	Herb
MOL001749	ZINC03860434	43.59	0.35	ADRB2	*F*. *cirrhosa*
MOL001787	Adenosine	15.98	0.18	ADA, ADORA2A	*F*. *cirrhosa*
MOL000223	Caffeic acid	25.76	0.05	ADRB2, BTK	*F*. *cirrhosa*
MOL000357	Sitogluside	20.63	0.62	ADRB2	*F*. *cirrhosa*
					*E*. *C*. *Grandis*
MOL000358	*β*-Sitosterol	36.91	0.99	ADRB2, PON1, TGFB1	*F*. *cirrhosa*
					*E*. *C*. *Grandis*
MOL000360	Ferulic acid	39.56	0.06	ADRB2	*F*. *cirrhosa*
MOL000069	Palmitic acid	19.3	0.1	IL10, PTEN	*F*. *cirrhosa*
					*F*. *Trichosanthis*
					*P*. *Cocos*
					*E*. *C*. *Grandis*
					*P*. *Grandiflora*
MOL009568	Methylbenzylideneacetone	30	0.03	ADRB2	*F*. *cirrhosa*
MOL009578	(Z)-3-(3,4,5-Trimethoxyphenyl) acrylic acid	20.45	0.09	ADRB2, NOS2	*F*. *cirrhosa*
MOL009594	Nemerol	77.48	0.1	ADRB2, DPP4	*F*. *cirrhosa*
MOL007549	*α*-Thymidine	10.39	0.11	DPP4	*F*. *cirrhosa*
MOL002083	Tricin	27.86	0.34	DPP4, NOS2, PRSS1	*F*. *Trichosanthis*
MOL002881	Diosmetin	31.14	0.27	DPP4, NOS2, PRSS1	*F*. *Trichosanthis*
MOL000305	Lauric acid	23.59	0.04	CD40, CD80, CD86, CXCL8, IL6	*F*. *Trichosanthis*
					*P*. *Cocos*
MOL003050	Nonanoic acid	40.51	0.02	ISYNA1	*F*. *Trichosanthis*
					*E*. *C*. *Grandis*
MOL000041	Phenylalanine	41.62	0.04	ADRB2, DPP4	*F*. *Trichosanthis*
MOL000432	Linolenic acid	45.01	0.15	ALB, PCNA, TRPV1	*F*. *Trichosanthis*
MOL004739	(2R)-2-Azaniumylpropanoate	85.17	0.01	CAT, MMP8	*F*. *Trichosanthis*
MOL000050	2-Azaniumylacetate	48.74	0	CAT, MMP8	*F*. *Trichosanthis*
MOL005530	Hydroxygenkwanin	36.47	0.27	DPP4, NOS2, PRSS1	*F*. *Trichosanthis*
MOL000056	(2S)-2-Azaniumyl-3-(4-hydroxyphenyl)propanoate	57.55	0.05	ADRB2, DPP4	*F*. *Trichosanthis*
MOL000635	Vanillin	52	0.03	MAPK1, MMP9	*F*. *Trichosanthis*
MOL000065	(3S)-3-Azaniumyl-4-hydroxy-4-oxobutanoate	79.74	0.02	ALOX5	*F*. *Trichosanthis*
MOL000675	Oleic acid	33.13	0.14	CAT, CRP, MPO, PON1, SOD1	*F*. *Trichosanthis*
MOL000879	Methyl palmitate	18.09	0.12	IL6, IL10	*F*. *Trichosanthis*
					*E*. *C*. *Grandis*
MOL000748	5-Hydroxymethylfurfural	45.07	0.02	DPP4	*F*. *Trichosanthis*
MOL002440	Cucurbitacin b	25.9	0.75	STAT3	*R*. *Trichosanthis*
MOL000388	4-Aminobutanoate	24.09	0.01	IL6, VCP	*R*. *Trichosanthis*
MOL000303	Caprylic acid	16.4	0.02	CXCL8	*P*. *Cocos*
MOL010856	Putrescine	81.23	0	NFKB1, TLR2	*E*. *C*. *Grandis*
MOL013277	Isosinensetin	51.15	0.44	ADRB2, CHEK1, DPP4, NOS2, PRSS1	*E*. *C*. *Grandis*
MOL013278	4',5,7,8-Tetramethoxyflavone	23.45	0.36	ADRB2, CHEK1, DPP4, NOS2, PRSS1	*E*. *C*. *Grandis*
MOL013279	5,7,4'-Trimethylapigenin	39.83	0.3	ADRB2, DPP4, NOS2, PRSS1	*E*. *C*. *Grandis*
MOL001803	Sinensetin	50.56	0.45	ADRB2, CHEK1, DPP4, NOS2, PRSS1	*E*. *C*. *Grandis*
MOL000193	(Z)-Caryophyllene	30.29	0.09	ADRB2	*E*. *C*. *Grandis*
MOL000254	Eugenol	56.24	0.04	ADRB2, ALOX5, CD86, MUC1	*E*. *C*. *Grandis*
MOL000269	Elemicin	21.94	0.06	ADRB2	*E*. *C*. *Grandis*
MOL004328	Naringenin	59.29	0.21	ABCC1, ADIPOQ, CAT, MAPK1, SOD1	*E*. *C*. *Grandis*
MOL000475	Anethole	32.49	0.03	ADRB2, IL2	*E*. *C*. *Grandis*
MOL005812	Naringin	6.92	0.78	CDKN1A	*E*. *C*. *Grandis*
MOL005814	Tangeretin	21.38	0.43	ADRB2, CDKN1A, CHEK1, DPP4, HMOX1, NOS2, PRSS1	*E*. *C*. *Grandis*
MOL005828	Nobiletin	61.67	0.52	CHEK1, DPP4, MMP9, NOS2, PRSS1, TIMP1	*E*. *C*. *Grandis*
MOL005849	Didymin	38.55	0.24	ADRB2	*E*. *C*. *Grandis*
MOL000008	Apigenin	23.06	0.21	CD40LG, CDKN1A, DPP4, HMOX1, ICAM1, IFNG, IL2, IL4, IL13, IKBKG, MMP1, MMP9, MS4A2, PRSS1	*E*. *C*. *Grandis*
MOL002003	(-)-Caryophyllene oxide	32.67	0.13	DPP4	*E*. *C*. *Grandis*
MOL006047	3-Butyl-4-methylphthalic acid	68.3	0.09	ADRB2	*P*. *Grandiflora*
MOL001689	Acacetin	34.97	0.24	ADRB2, CDKN1A, CHEK1, DPP4, FASLG, NOS2, PRSS1	*P*. *Grandiflora*
MOL005999	Crotonaldehyde	64.99	0	CSF2, IFNG, IL2, IL10	*P*. *Grandiflora*
MOL000006	Luteolin	36.16	0.25	CD40LG, CDKN1A, DPP4, HMOX1, ICAM1, IFNG, IL2, IL4, IL6, IL10, MAPK1, MMP1, MMP2, MMP9, PCNA, PRSS1	*P*. *Grandiflora*

**Table 2:** Selected core targets and their topological parameters.

Gene	Betweenness	Closeness	Degree
IL6	0.083545	0.892857	44
ALB	0.062818	0.833333	40
IL10	0.042936	0.819672	39
MMP9	0.050301	0.806452	38
CXCL8	0.025899	0.78125	37
MAPK1	0.041765	0.769231	35
STAT3	0.014888	0.757576	35
IL4	0.018709	0.746269	34
ICAM1	0.016277	0.746269	33
IL2	0.013697	0.746269	33
TLR2	0.010204	0.735294	33
MPO	0.031878	0.735294	32
IL13	0.023169	0.714286	32
IFNG	0.008525	0.714286	31
CRP	0.015704	0.714286	30
MMP2	0.007624	0.694444	28
CAT	0.058313	0.666667	26
HMOX1	0.006373	0.666667	26

**Table 3:** The details and scores of systems docking analysis.

Protein name	PDB ID	Test compounds	Docking scores
ALB	4EMX	Sitogluside	8.067
ALB	4EMX	Cucurbitacin b	7.575
ALB	4EMX	Beta-sitosterol	7.428
ICAM1	3TCX	Beta-sitosterol	8.34
ICAM1	3TCX	Didymin	8.247
ICAM1	3TCX	Naringin	8.247
ICAM1	3TCX	Cucurbitacin b	8.133
ICAM1	3TCX	Sitogluside	8.054
IL10	2ILK	Apigenin	7.812
IL10	2ILK	Naringenin	7.726
IL10	2ILK	Luteolin	7.262
MAPK1	6G9A	Cucurbitacin b	8.077
MAPK1	6G9A	Sitogluside	8.073
MAPK1	6G9A	Beta-sitosterol	7.718

**Table 4:** AutoDock docking results for binding free energy (FE) and inhibition constant (*K*_i_).

Protein name	Test compounds	FE (kcal/mol)	Ki (*μ*M)
ALB	Sitogluside	−7.77	2.01
ALB	Cucurbitacin b	−9.19	0.18217
ALB	Beta-sitosterol	−9.61	0.09007
ICAM1	Beta-sitosterol	−8.35	0.75731
ICAM1	Didymin	−6.84	9.65
ICAM1	Naringin	−4.93	242.67
ICAM1	Cucurbitacin b	−8.87	0.31278
ICAM1	Sitogluside	−8.31	0.81154
IL10	Apigenin	−6.35	21.98
IL10	Naringenin	−5.91	46.56
IL10	Luteolin	−5.67	69.62
MAPK1	Cucurbitacin b	−7.43	3.58
MAPK1	Sitogluside	−8.25	0.89856
MAPK1	Beta-sitosterol	−8.73	0.40161

## Data Availability

The raw data and materials in this study are available from the corresponding author on reasonable request.

## Supplementary Materials

Supplementary Materials Supplementary Table 1: the chemical compounds of 6 herbs in BMGLF. Supplementary Table 2: the targets of BMGLF and bronchiectasis. Supplementary Table 3: the data of GO enrichment analysis. Supplementary Table 4: the data of KEGG pathway enrichment analysis.
