# Supplementary material for: Network Pharmacology Analysis of the Therapeutic Mechanisms Underlying Beimu-Gualou Formula Activity against Bronchiectasis with In Silico Molecular Docking Validation
Source: Evid Based Complement Alternat Med. 2021 Jan 5;2021:3656272. doi: 10.1155/2021/3656272 (PMC7803403; doi:10.1155/2021/3656272)
Supplement: Supplementary Materials — Supplementary Table 1: the chemical compounds of 6 herbs in BMGLF. Supplementary Table 2: the targets of BMGLF and bronchiectasis. Supplementary Table 3: the data of GO enrichment analysis. Supplementary Table 4: the data of KEGG pathway enrichment analysis. [file 3656272.f1.zip › 3656272.f1/Supplementary Table 1.docx]

| **A list of chemical compounds of 6 herbs in BMGLF** | | | | | | | | | | | | |
| --- | --- | --- | --- | --- | --- | --- | --- | --- | --- | --- | --- | --- |
| **Herb** | **Mol ID** | **Molecule Name** | **MW** | **AlogP** | **Hdon** | **Hacc** | **OB (%)** | **Caco-2** | **BBB** | **DL** | **FASA-** | **HL** |
| *Fritillaria Cirrhosa* | MOL001404 | Pentatriacontane | 493.07 | 16.34 | 0 | 0 | 7.97 | 1.91 | 1.48 | 0.42 | 0.11 |  |
| *Fritillaria Cirrhosa* | MOL001744 | uracil | 112.1 | -1.01 | 2 | 4 | 42.53 | 0.05 | -0.08 | 0.02 | 0 | 11.77 |
| *Fritillaria Cirrhosa* | MOL001749 | ZINC03860434 | 390.62 | 7.57 | 0 | 4 | 43.59 | 1.04 | 0.6 | 0.35 | 0.3 | 3.97 |
| *Fritillaria Cirrhosa* | MOL001787 | ADO | 267.28 | -2.02 | 5 | 8 | 15.98 | -1.56 | -2.22 | 0.18 | 0.23 |  |
| *Fritillaria Cirrhosa* | MOL000223 | caffeic acid | 180.17 | 1.37 | 3 | 4 | 25.76 | 0.21 | -0.26 | 0.05 | 0.44 |  |
| *Fritillaria Cirrhosa* | MOL002295 | cinnamic acid | 148.17 | 1.9 | 1 | 2 | 19.68 | 0.91 | 0.96 | 0.03 | 0.47 |  |
| *Fritillaria Cirrhosa* | MOL002687 | guanosine | 283.28 | -2.41 | 6 | 9 | 21.43 | -1.21 | -1.62 | 0.21 | 0.23 |  |
| *Fritillaria Cirrhosa* | MOL002703 | OCTADECENE | 252.54 | 8.19 | 0 | 0 | 19.21 | 1.88 | 1.98 | 0.09 | 0.19 |  |
| *Fritillaria Cirrhosa* | MOL000357 | Sitogluside | 576.95 | 6.34 | 4 | 6 | 20.63 | -0.14 | -0.93 | 0.62 | 0.23 |  |
| *Fritillaria Cirrhosa* | MOL000358 | beta-sitosterol | 414.79 | 8.08 | 1 | 1 | 36.91 | 1.32 | 0.99 | 0.75 | 0.23 | 5.36 |
| *Fritillaria Cirrhosa* | MOL000359 | sitosterol | 414.79 | 8.08 | 1 | 1 | 36.91 | 1.32 | 0.87 | 0.75 | 0.22 | 5.37 |
| *Fritillaria Cirrhosa* | MOL000360 | FER | 194.2 | 1.62 | 2 | 4 | 39.56 | 0.47 | -0.03 | 0.06 | 0.34 | 2.38 |
| *Fritillaria Cirrhosa* | MOL004440 | Peimisine | 427.69 | 3.16 | 2 | 4 | 57.4 | 0.18 | -0.45 | 0.81 | 0.23 | 14.39 |
| *Fritillaria Cirrhosa* | MOL004445 | Verticine | 431.73 | 3.4 | 3 | 4 | 17.42 | 0.05 | -0.68 | 0.67 | 0.19 |  |
| *Fritillaria Cirrhosa* | MOL004448 | Solatubin | 397.71 | 5.27 | 1 | 2 | 17.12 | 1.22 | 0.94 | 0.76 | 0.19 |  |
| *Fritillaria Cirrhosa* | MOL004649 | 1-[(2R,3R,4S,5S)-3,4-dihydroxy-5-(hydroxymethyl)oxolan-2-yl]pyrimidine-2,4-dione | 244.23 | -2.45 | 4 | 8 | 17.85 | -1.19 | -1.69 | 0.11 | 0.29 |  |
| *Fritillaria Cirrhosa* | MOL004791 | Ethol | 242.5 | 6.45 | 1 | 1 | 13.32 | 1.31 | 1.07 | 0.08 | 0.15 |  |
| *Fritillaria Cirrhosa* | MOL005705 | Eicosanol | 298.62 | 8.27 | 1 | 1 | 12.1 | 1.37 | 0.86 | 0.16 | 0.17 |  |
| *Fritillaria Cirrhosa* | MOL000069 | palmitic acid | 256.48 | 6.37 | 1 | 2 | 19.3 | 1.09 | 1 | 0.1 | 0 |  |
| *Fritillaria Cirrhosa* | MOL006948 | inosine | 268.26 | -2.22 | 4 | 8 | 11.17 | -1.12 | -1.49 | 0.18 | 0.23 |  |
| *Fritillaria Cirrhosa* | MOL006953 | Thy | 126.13 | -0.56 | 2 | 4 | 74.2 | 0.24 | 0.12 | 0.02 | 0.33 | 11.65 |
| *Fritillaria Cirrhosa* | MOL007549 | alpha-Thymidine | 242.26 | -1.24 | 3 | 7 | 10.39 | -0.64 | -1.07 | 0.11 | 0.26 |  |
| *Fritillaria Cirrhosa* | MOL000771 | p-coumaric acid | 164.17 | 1.64 | 2 | 3 | 43.29 | 0.46 | 0.13 | 0.04 | 0.45 | 4.43 |
| *Fritillaria Cirrhosa* | MOL008245 | DODECENE | 168.36 | 5.46 | 0 | 0 | 17.74 | 1.8 | 2.05 | 0.02 | 0.22 |  |
| *Fritillaria Cirrhosa* | MOL000842 | sucrose | 342.34 | -4.31 | 8 | 11 | 7.17 | -2.89 | -6.67 | 0.23 | 0.2 |  |
| *Fritillaria Cirrhosa* | MOL001837 | Methyl-p-coumarate | 178.2 | 1.89 | 1 | 3 | 20.14 | 0.83 | 0.5 | 0.05 | 0.37 |  |
| *Fritillaria Cirrhosa* | MOL000858 | Glycerol palmitate | 330.57 | 5.57 | 2 | 4 | 26.66 | 0.17 | -0.5 | 0.22 | 0.17 |  |
| *Fritillaria Cirrhosa* | MOL000860 | stearic acid | 284.54 | 7.28 | 1 | 2 | 17.83 | 1.15 | 1.22 | 0.14 | 0.19 |  |
| *Fritillaria Cirrhosa* | MOL009027 | Cyclopamine | 411.69 | 4.26 | 2 | 3 | 55.42 | 0.77 | 0 | 0.82 | 0.22 | 14.67 |
| *Fritillaria Cirrhosa* | MOL009566 | 1,2-Epoxyoctadecane | 268.54 | 7.16 | 0 | 1 | 12.75 | 1.65 | 1.42 | 0.12 | 0.15 |  |
| *Fritillaria Cirrhosa* | MOL009567 | 1-O-feruloyl glycerol | 266.32 | 1.02 | 3 | 5 | 13.22 | 0.19 | -0.72 | 0.13 | 0.3 |  |
| *Fritillaria Cirrhosa* | MOL009568 | Methylbenzylideneacetone | 160.23 | 2.34 | 0 | 1 | 30 | 1.51 | 1.58 | 0.03 | 0.46 |  |
| *Fritillaria Cirrhosa* | MOL009569 | 4,7-DIMETHYLBENZOFURAN | 146.2 | 3.11 | 0 | 1 | 55.66 | 1.8 | 1.82 | 0.04 | 0.26 | 5.88 |
| *Fritillaria Cirrhosa* | MOL009570 | Methyl stearolate | 294.53 | 7.21 | 0 | 2 | 28.18 | 1.46 | 1.04 | 0.17 | 0.18 |  |
| *Fritillaria Cirrhosa* | MOL009572 | Chuanbeinone | 413.71 | 4.23 | 1 | 3 | 41.07 | 0.61 | 0.08 | 0.71 | 0.19 | 8.07 |
| *Fritillaria Cirrhosa* | MOL009573 | cycloposine | 573.85 | 2.52 | 5 | 8 | 12.77 | -0.51 | -1.34 | 0.29 | 0.22 |  |
| *Fritillaria Cirrhosa* | MOL009574 | 4-amino-1-[(2R,3R,4S,5S)-3,4-dihydroxy-5-(hydroxymethyl)oxolan-2-yl]pyrimidin-2-one | 243.25 | -2.43 | 5 | 8 | 10.32 | -0.82 | -1.23 | 0.11 | 0.26 |  |
| *Fritillaria Cirrhosa* | MOL009575 | delavine | 479.68 | -0.94 | 1 | 8 | 7.52 | 0.25 | 0.06 | 0.36 | 0.11 |  |
| *Fritillaria Cirrhosa* | MOL009576 | ebeiedinone | 398.72 | 5.35 | 1 | 1 | 16.63 | 0.59 | 0.34 | 0.74 | 0.17 |  |
| *Fritillaria Cirrhosa* | MOL009577 | Aklavinon | 412.42 | 1.6 | 4 | 8 | 20.25 | -0.65 | -1.27 | 0.78 | 0.33 |  |
| *Fritillaria Cirrhosa* | MOL009578 | (Z)-3-(3,4,5-trimethoxyphenyl)acrylic acid | 238.26 | 1.85 | 1 | 5 | 20.45 | 0.71 | 0.52 | 0.09 | 0.23 |  |
| *Fritillaria Cirrhosa* | MOL009579 | ent-(16S)-atisan-13,17-oxide | 288.52 | 4.27 | 0 | 1 | 47.74 | 1.53 | 1.62 | 0.43 | 0.18 | 8.22 |
| *Fritillaria Cirrhosa* | MOL009580 | ent-17-norkauran-16-one | 274.49 | 4.22 | 0 | 1 | 16.35 | 1.31 | 1.49 | 0.28 | 0.24 |  |
| *Fritillaria Cirrhosa* | MOL009581 | ent-kaur-15-en-17-ol | 288.52 | 4.49 | 1 | 1 | 13.59 | 1.2 | 1.11 | 0.31 | 0.21 |  |
| *Fritillaria Cirrhosa* | MOL009582 | Sipeimine | 429.71 | 3.21 | 2 | 4 | 16.18 | 0.27 | -0.51 | 0.67 | 0.2 |  |
| *Fritillaria Cirrhosa* | MOL009583 | STOCK1N-49993 | 591.87 | 1.47 | 5 | 9 | 4.74 | -1.08 | -1.78 | 0.21 | 0.2 |  |
| *Fritillaria Cirrhosa* | MOL009584 | imperialine-3β-D-glucoside_qt | 429.71 | 3.21 | 2 | 4 | 14.24 | 0.31 | -0.45 | 0.67 | 0.2 |  |
| *Fritillaria Cirrhosa* | MOL009585 | Imperialine-β-N-oxide | 445.71 | 0.96 | 2 | 4 | 15.71 | 0.16 | -0.53 | 0.64 | 0.03 |  |
| *Fritillaria Cirrhosa* | MOL009586 | isoverticine | 431.73 | 3.4 | 3 | 4 | 48.23 | 0.27 | -0.44 | 0.67 | 0.16 | 7.9 |
| *Fritillaria Cirrhosa* | MOL009587 | isoverticine-beta-N-oxide | 447.73 | 1.15 | 3 | 4 | 22.35 | 0.14 | -0.44 | 0.63 | 0.01 |  |
| *Fritillaria Cirrhosa* | MOL009588 | Korseveriline | 431.73 | 3.33 | 3 | 4 | 35.16 | 0.25 | -0.46 | 0.68 | 0.18 | 8.03 |
| *Fritillaria Cirrhosa* | MOL009589 | Korseverinine | 413.71 | 4.36 | 2 | 3 | 53.51 | 0.34 | -0.43 | 0.71 | 0.18 | 9.23 |
| *Fritillaria Cirrhosa* | MOL009590 | Korsevinine | 427.69 | 4 | 1 | 4 | 22.15 | 0.28 | -0.42 | 0.85 | 0.24 |  |
| *Fritillaria Cirrhosa* | MOL009591 | Korsine | 429.71 | 3.52 | 3 | 4 | 22.94 | -0.11 | -0.92 | 0.67 | 0.2 |  |
| *Fritillaria Cirrhosa* | MOL009592 | Kosamol A | 526.68 | 5.77 | 6 | 8 | 16.8 | -0.38 | -1 | 0.77 | 0.33 |  |
| *Fritillaria Cirrhosa* | MOL009593 | verticinone | 429.71 | 3.21 | 2 | 4 | 60.07 | 0.42 | -0.29 | 0.67 | 0.19 | 7.07 |
| *Fritillaria Cirrhosa* | MOL009594 | Nemerol | 247.37 | 2.45 | 0 | 3 | 77.48 | 1.18 | 1.36 | 0.1 | 0.3 | 3.78 |
| *Fritillaria Cirrhosa* | MOL009595 | 2-(chloromethyl)-7-methyl-[1,3,4]thiadiazolo[2,3-b]pyrimidin-5-one | 215.68 | 1.76 | 0 | 4 | 24.61 | 0.68 | 0.5 | 0.06 | 0.5 |  |
| *Fritillaria Cirrhosa* | MOL009596 | sinpemine A | 413.71 | 4.23 | 1 | 3 | 46.96 | 0.76 | 0.24 | 0.71 | 0.2 | 8.49 |
| *Fritillaria Cirrhosa* | MOL009597 | solanidine 3-O-α-L-rhamnopyranosyl-(1-2)-[β-D-glucopyranosyl-(1-4)]-β-D-glucopyranoside | 868.19 | 0.92 | 9 | 16 | 17.77 | -2.29 | -3.31 | 0.03 | 0.21 |  |
| *Fritillaria Cirrhosa* | MOL009598 | solanine | 412.7 | 4.82 | 0 | 2 | 0.32 | 1.29 | 0.54 | 0.49 | 0.24 |  |
| *Fritillaria Cirrhosa* | MOL009599 | songbeinone | 413.71 | 4.23 | 1 | 3 | 45.35 | 0.63 | 0.02 | 0.71 | 0.19 | 7.64 |
| *Fritillaria Cirrhosa* | MOL009600 | songbeisine | 427.69 | 3.58 | 2 | 4 | 13.48 | 0.31 | -0.25 | 0.81 | 0.23 |  |
| *Fructus Trichosanthis* | MOL000114 | vanillic acid | 168.16 | 1.15 | 2 | 4 | 35.47 | 0.43 | 0.09 | 0.04 | 0.34 | 11.62 |
| *Fructus Trichosanthis* | MOL001393 | myristic acid | 228.42 | 5.46 | 1 | 2 | 21.18 | 1.07 | 0.99 | 0.07 | 0.19 |  |
| *Fructus Trichosanthis* | MOL001398 | Methyllinolenate | 292.51 | 6.2 | 0 | 2 | 46.15 | 1.48 | 1.09 | 0.17 | 0.24 | 5.95 |
| *Fructus Trichosanthis* | MOL001494 | Mandenol | 308.56 | 6.99 | 0 | 2 | 42 | 1.46 | 1.14 | 0.19 | 0.25 | 5.39 |
| *Fructus Trichosanthis* | MOL001640 | NON | 172.3 | 3.63 | 1 | 2 | 26.74 | 0.96 | 1.06 | 0.03 | 0.22 |  |
| *Fructus Trichosanthis* | MOL001641 | METHYL LINOLEATE | 294.53 | 6.64 | 0 | 2 | 41.93 | 1.44 | 1.08 | 0.17 | 0.21 | 6.05 |
| *Fructus Trichosanthis* | MOL001739 | zoomaric acid | 254.46 | 5.92 | 1 | 2 | 35.78 | 1.18 | 0.88 | 0.1 | 0.24 | 5.29 |
| *Fructus Trichosanthis* | MOL002038 | 9E,12Z-octadecadienoic acid | 280.5 | 6.39 | 1 | 2 | 41.9 | 1.16 | 0.77 | 0.14 | 0.25 | 5.36 |
| *Fructus Trichosanthis* | MOL002083 | tricin | 330.31 | 2.3 | 3 | 7 | 27.86 | 0.51 | -0.6 | 0.34 | 0.27 |  |
| *Fructus Trichosanthis* | MOL002254 | Barolub | 807.49 | 19.52 | 0 | 6 | 16.29 | 0.88 | -0.06 | 0.22 | 0.21 |  |
| *Fructus Trichosanthis* | MOL002682 | aldehydo-D-galactose | 180.18 | -2.68 | 5 | 6 | 47.81 | -1.67 | -4.26 | 0.03 | 0.25 | 7.97 |
| *Fructus Trichosanthis* | MOL002881 | Diosmetin | 300.28 | 2.32 | 3 | 6 | 31.14 | 0.46 | -0.66 | 0.27 | 0.34 | 16.34 |
| *Fructus Trichosanthis* | MOL000003 | MTL | 182.2 | -2.94 | 6 | 6 | 17.73 | -1.58 | -4.07 | 0.03 | 0.18 |  |
| *Fructus Trichosanthis* | MOL000305 | lauric acid | 200.36 | 4.54 | 1 | 2 | 23.59 | 1.02 | 1.1 | 0.04 | 0 |  |
| *Fructus Trichosanthis* | MOL003050 | nonanoic acid | 158.27 | 3.17 | 1 | 2 | 40.51 | 0.92 | 1.08 | 0.02 | 0.23 | 4.15 |
| *Fructus Trichosanthis* | MOL003969 | L-Serin | 105.11 | -1.49 | 4 | 4 | 98.47 | -1.12 | -2.96 | 0.01 | 0.31 | 11.52 |
| *Fructus Trichosanthis* | MOL003971 | Threonin | 119.14 | -1.11 | 4 | 4 | 73.52 | -0.87 | -2.56 | 0.01 | 0.3 | 11.42 |
| *Fructus Trichosanthis* | MOL000041 | PHA | 165.21 | 0.96 | 3 | 3 | 41.62 | 0.36 | 0.22 | 0.04 | 0 | 4.62 |
| *Fructus Trichosanthis* | MOL000432 | linolenic acid | 278.48 | 5.95 | 1 | 2 | 45.01 | 1.21 | 0.84 | 0.15 | 0 | 5.54 |
| *Fructus Trichosanthis* | MOL004355 | Spinasterol | 412.77 | 7.64 | 1 | 1 | 42.98 | 1.44 | 1.04 | 0.76 | 0.21 | 5.32 |
| *Fructus Trichosanthis* | MOL000458 | campesterol | 400.76 | 7.97 | 1 | 1 | 5.57 | 1.6 | 1.41 | 0.72 | 0.22 |  |
| *Fructus Trichosanthis* | MOL004690 | RNS | 164.18 | -1.8 | 4 | 5 | 40.73 | -1.3 | -3.7 | 0.03 | 0.31 | 11.06 |
| *Fructus Trichosanthis* | MOL004691 | aldehydo-D-ribose | 150.15 | -2.17 | 4 | 5 | 40.76 | -1.59 | -4.17 | 0.02 | 0.3 | 11.26 |
| *Fructus Trichosanthis* | MOL004739 | DAL | 89.11 | -0.6 | 3 | 3 | 85.17 | -0.3 | -0.51 | 0.01 | 0.33 | 11.61 |
| *Fructus Trichosanthis* | MOL000050 | GLY | 75.08 | -0.98 | 3 | 3 | 48.74 | -0.56 | -1.03 | 0 | 0 | 11.95 |
| *Fructus Trichosanthis* | MOL000515 | Melissic acid | 452.9 | 12.75 | 1 | 2 | 13.22 | 1.31 | 0.9 | 0.49 | 0.18 |  |
| *Fructus Trichosanthis* | MOL000052 | Gulutamine | 147.15 | -0.92 | 4 | 5 | 6.66 | -1.05 | -1.97 | 0.02 | 0 |  |
| *Fructus Trichosanthis* | MOL000054 | L- | 174.24 | -1.11 | 7 | 6 | 47.64 | -0.49 | -1.04 | 0.03 | 0 | 0.85 |
| *Fructus Trichosanthis* | MOL005448 | Leucinum | 131.2 | 0.63 | 3 | 3 | 72.92 | -0.05 | -0.37 | 0.01 | 0.29 | 11.41 |
| *Fructus Trichosanthis* | MOL005449 | h-Met-h | 149.24 | -0.27 | 3 | 3 | 70.87 | 0.06 | -0.17 | 0.01 | 0.37 | 11.69 |
| *Fructus Trichosanthis* | MOL000055 | L-Lysin | 146.22 | -0.68 | 5 | 4 | 29.33 | -0.66 | -1.44 | 0.02 | 0 |  |
| *Fructus Trichosanthis* | MOL005530 | Hydroxygenkwanin | 300.28 | 2.32 | 3 | 6 | 36.47 | 0.52 | -0.44 | 0.27 | 0.31 | 15.22 |
| *Fructus Trichosanthis* | MOL000056 | DTY | 181.21 | 0.69 | 4 | 4 | 57.55 | -0.1 | -0.49 | 0.05 | 0 | 0.96 |
| *Fructus Trichosanthis* | MOL000602 | FUM | 116.08 | -0.01 | 2 | 4 | 17.74 | -0.59 | -0.99 | 0.01 | 0.39 |  |
| *Fructus Trichosanthis* | MOL000061 | Prolinum | 115.15 | -0.06 | 2 | 3 | 77.57 | 0.22 | 0.29 | 0.01 | 0 | 11.13 |
| *Fructus Trichosanthis* | MOL000635 | vanillin | 152.16 | 1.31 | 1 | 3 | 52 | 0.68 | 0.41 | 0.03 | 0.33 | 11.79 |
| *Fructus Trichosanthis* | MOL000065 | ASI | 133.12 | -1.25 | 4 | 5 | 79.74 | -1.02 | -1.53 | 0.02 | 0 | 11.38 |
| *Fructus Trichosanthis* | MOL000659 | Montanic acid | 424.84 | 11.84 | 1 | 2 | 13.69 | 1.28 | 0.98 | 0.46 | 0.16 |  |
| *Fructus Trichosanthis* | MOL000662 | Ceric acid | 396.78 | 10.93 | 1 | 2 | 14.24 | 1.26 | 1 | 0.4 | 0.18 |  |
| *Fructus Trichosanthis* | MOL000663 | lignoceric acid | 368.72 | 10.02 | 1 | 2 | 14.9 | 1.24 | 1.01 | 0.33 | 0.17 |  |
| *Fructus Trichosanthis* | MOL000067 | L-Valin | 117.17 | 0.24 | 3 | 3 | 53.33 | 0.04 | -0.14 | 0.01 | 0 | 11.34 |
| *Fructus Trichosanthis* | MOL000675 | oleic acid | 282.52 | 6.84 | 1 | 2 | 33.13 | 1.17 | 0.78 | 0.14 | 0.2 | 4.99 |
| *Fructus Trichosanthis* | MOL006756 | Schottenol | 414.79 | 8.08 | 1 | 1 | 37.42 | 1.33 | 0.91 | 0.75 | 0.22 | 5.63 |
| *Fructus Trichosanthis* | MOL000068 | L-Ile | 131.2 | 0.7 | 3 | 3 | 59.05 | 0.06 | -0.11 | 0.02 | 0 | 11.21 |
| *Fructus Trichosanthis* | MOL000069 | palmitic acid | 256.48 | 6.37 | 1 | 2 | 19.3 | 1.09 | 1 | 0.1 | 0 |  |
| *Fructus Trichosanthis* | MOL006946 | adenosine | 255.27 | -2.35 | 6 | 8 | 19.85 | -1.49 | -2.09 | 0.16 | 0.21 |  |
| *Fructus Trichosanthis* | MOL000071 | Istidina | 155.18 | -1.01 | 4 | 4 | 53.18 | -0.25 | -0.4 | 0.03 | 0 | -5.72 |
| *Fructus Trichosanthis* | MOL007161 | 1-tri-chosanoyl-2- linolenic-3-palmitoyl-glucerin | 851.49 | 18.68 | 0 | 6 | 36.04 | 0.9 | -0.98 | 0.15 | 0.22 | -0.86 |
| *Fructus Trichosanthis* | MOL007162 | 1-tri-chosanoyl-2-linoleoyl-3-palmitoyl-glucerin | 853.51 | 19.12 | 0 | 6 | 33.97 | 0.87 | -0.8 | 0.16 | 0.22 | -0.22 |
| *Fructus Trichosanthis* | MOL007163 | 1-trichosanoyl-2,3-dilinoleoyl-glycerin | 877.53 | 19.15 | 0 | 6 | 36.98 | 0.73 | -1.05 | 0.14 | 0.25 | 3.11 |
| *Fructus Trichosanthis* | MOL007164 | 1-trichosanoyl-2,3-linolenic-glycerin | 873.49 | 18.26 | 0 | 6 | 38.92 | 1.02 | -0.79 | 0.13 | 0.24 | 0.34 |
| *Fructus Trichosanthis* | MOL007165 | 10α-cucurbita-5,24-diene-3β-ol | 426.8 | 7.93 | 1 | 1 | 44.02 | 1.46 | 1.04 | 0.74 | 0.24 | 5.32 |
| *Fructus Trichosanthis* | MOL007166 | 1,3-ditrichosanoyl-2-linoleoyl-glycerin | 875.51 | 18.7 | 0 | 6 | 37.81 | 1 | -0.96 | 0.14 | 0.25 | -1.11 |
| *Fructus Trichosanthis* | MOL007167 | 2,2'bioxazolidine-3,3'-diethanol | 232.32 | -0.84 | 2 | 6 | 3.51 | -0.33 | -0.8 | 0.09 | 0.12 |  |
| *Fructus Trichosanthis* | MOL007168 | Odhpca | 139.12 | 0.02 | 2 | 4 | 43.07 | 0.19 | 0.03 | 0.03 | 0.31 | 11.99 |
| *Fructus Trichosanthis* | MOL007169 | 4-hydroxy-2-methoxybenzoic acid | 168.16 | 1.15 | 2 | 4 | 50.76 | 0.5 | 0.28 | 0.04 | 0.33 | 11.79 |
| *Fructus Trichosanthis* | MOL007170 | cirsiumaldehyde | 234.22 | 2.11 | 0 | 5 | 41.38 | -0.03 | 0.03 | 0.11 | 0.2 | 7.81 |
| *Fructus Trichosanthis* | MOL007171 | 5-dehydrokarounidiol | 438.76 | 5.71 | 2 | 2 | 30.23 | 0.71 | 0.05 | 0.77 | 0.24 | 2.95 |
| *Fructus Trichosanthis* | MOL007172 | 7-oxo-dihydrokaro-unidiol | 456.78 | 5.27 | 2 | 3 | 36.85 | 0.25 | -0.74 | 0.75 | 0.23 | 2.68 |
| *Fructus Trichosanthis* | MOL007173 | beta-D-arabinopyranose | 150.15 | -2 | 4 | 5 | 52.64 | -1.32 | -3.51 | 0.03 | 0.25 | 11.02 |
| *Fructus Trichosanthis* | MOL007174 | 5-hydroxy-2-(3-hydroxy-4-methoxyphenyl)-7-[(2S,3R,4S,5S,6R)-3,4,5-trihydroxy-6-(hydroxymethyl)oxan-2-yl]oxychromen-4-one | 462.44 | 0.41 | 6 | 11 | 28.08 | -1 | -2.07 | 0.82 | 0.27 |  |
| *Fructus Trichosanthis* | MOL007175 | karounidiol 3-o-benzoate | 544.89 | 8.01 | 1 | 3 | 43.99 | 0.84 | -0.05 | 0.5 | 0.3 | 3.31 |
| *Fructus Trichosanthis* | MOL007176 | ARA | 150.15 | -2 | 4 | 5 | 46.48 | -1.31 | -2.9 | 0.03 | 0.26 | 11.11 |
| *Fructus Trichosanthis* | MOL007177 | FCY | 121.18 | -0.52 | 3 | 3 | 22.1 | -0.37 | -0.75 | 0.01 | 0.43 |  |
| *Fructus Trichosanthis* | MOL007178 | 1,4-D-Galactonolactone | 178.16 | -2.12 | 4 | 6 | 59.77 | -1.54 | -4.3 | 0.04 | 0.31 | 11.45 |
| *Fructus Trichosanthis* | MOL007179 | Linolenic acid ethyl ester | 306.54 | 6.55 | 0 | 2 | 46.1 | 1.48 | 1.09 | 0.2 | 0.24 | 5.8 |
| *Fructus Trichosanthis* | MOL007180 | vitamin-e | 490.69 | 3.78 | 3 | 9 | 32.29 | 0.38 | -0.58 | 0.7 | 0.33 | 0.04 |
| *Fructus Trichosanthis* | MOL007181 | Punicic acid | 278.48 | 5.95 | 1 | 2 | 44.9 | 1.21 | 0.87 | 0.15 | 0.28 | 5.91 |
| *Fructus Trichosanthis* | MOL007182 | 5-hydroxy-2-[4-hydroxy-3-[(2S,3R,4S,5S,6R)-3,4,5-trihydroxy-6-(hydroxymethyl)oxan-2-yl]oxyphenyl]-7-methoxychromen-4-one | 462.44 | 0.41 | 6 | 11 | 8.01 | -1.08 | -2.15 | 0.83 | 0.27 |  |
| *Fructus Trichosanthis* | MOL007183 | dibutyl (2R)-2-hydroxybutanedioate | 246.34 | 2.2 | 1 | 5 | 42.99 | 0.09 | -0.34 | 0.07 | 0.26 | 3.63 |
| *Fructus Trichosanthis* | MOL007184 | [(2R,5S)-5-(hydroxymethyl)oxolan-2-yl]methanol | 132.18 | -0.51 | 2 | 3 | 72.52 | -0.2 | -1.06 | 0.02 | 0.18 | 10.87 |
| *Fructus Trichosanthis* | MOL007185 | isokarounidiol | 440.78 | 5.96 | 2 | 2 | 29.16 | 0.77 | 0.11 | 0.77 | 0.22 |  |
| *Fructus Trichosanthis* | MOL007186 | Karounidiol | 440.78 | 5.96 | 2 | 2 | 26.26 | 0.77 | -0.08 | 0.77 | 0.22 |  |
| *Fructus Trichosanthis* | MOL007187 | stigmast-7,22-dien-3β-o-d-glucoside | 574.93 | 5.89 | 4 | 6 | 21.2 | -0.19 | -0.92 | 0.62 | 0.21 |  |
| *Fructus Trichosanthis* | MOL000731 | XYS | 150.15 | -2 | 4 | 5 | 58.74 | -1.16 | -3.18 | 0.03 | 0.22 | 11.08 |
| *Fructus Trichosanthis* | MOL000734 | GLO | 180.18 | -2.68 | 5 | 6 | 24.44 | -1.93 | -4.65 | 0.03 | 0.25 |  |
| *Fructus Trichosanthis* | MOL000748 | HMF | 126.12 | 0.67 | 1 | 3 | 45.07 | 0.05 | -0.27 | 0.02 | 0.22 | 11.73 |
| *Fructus Trichosanthis* | MOL000860 | stearic acid | 284.54 | 7.28 | 1 | 2 | 17.83 | 1.15 | 1.22 | 0.14 | 0.19 |  |
| *Fructus Trichosanthis* | MOL000879 | methyl palmitate | 270.51 | 6.62 | 0 | 2 | 18.09 | 1.37 | 1.18 | 0.12 | 0.14 |  |
| *Fructus Trichosanthis* | MOL000971 | Ethylpalmitate | 284.54 | 6.97 | 0 | 2 | 18.99 | 1.41 | 1.15 | 0.14 | 0.16 |  |
| *Radix Trichosanthis* | MOL002440 | cucurbitacin b | 558.78 | 2 | 3 | 8 | 25.9 | -0.87 | -1.58 | 0.75 | 0.31 |  |
| *Radix Trichosanthis* | MOL002805 | Elatericin A | 516.74 | 1.62 | 4 | 7 | 16.85 | -1.28 | -1.77 | 0.79 | 0 |  |
| *Radix Trichosanthis* | MOL003800 | CIR | 175.22 | -1.32 | 6 | 6 | 52.96 | -0.86 | -1.46 | 0.03 | 0.31 | 0.9 |
| *Radix Trichosanthis* | MOL000388 | gamma-aminobutyric acid | 103.14 | -0.62 | 3 | 3 | 24.09 | -0.26 | -0.57 | 0.01 | 0 |  |
| *Radix Trichosanthis* | MOL004355 | Spinasterol | 412.77 | 7.64 | 1 | 1 | 42.98 | 1.44 | 1.04 | 0.76 | 0.21 | 5.32 |
| *Radix Trichosanthis* | MOL004652 | (2R,3R,4S,5S,6R)-2-[[(3S,5S,9R,10S,13R,14R,17R)-17-[(E,2R,5S)-5-ethyl-6-methylhept-3-en-2-yl]-10,13-dimethyl-2,3,4,5,6,9,11,12,14,15,16,17-dodecahydro-1H-cyclopenta[a]phenanthren-3-yl]oxy]-6-(hydroxymethyl)oxane-3,4,5-triol | 574.93 | 5.89 | 4 | 6 | 21.2 | -0.2 | -0.99 | 0.63 | 0.21 |  |
| *Radix Trichosanthis* | MOL005367 | GUP | 180.18 | -2.51 | 5 | 6 | 43.04 | -1.82 | -4.46 | 0.04 | 0.26 | 11.1 |
| *Radix Trichosanthis* | MOL006755 | DIHYDROCUCURBITACIN B | 560.8 | 1.89 | 3 | 8 | 23.15 | -1 | -1.53 | 0.75 | 0.3 |  |
| *Radix Trichosanthis* | MOL006756 | Schottenol | 414.79 | 8.08 | 1 | 1 | 37.42 | 1.33 | 0.91 | 0.75 | 0.22 | 5.63 |
| *Radix Trichosanthis* | MOL006757 | Bryonolic acid | 456.78 | 6.62 | 2 | 3 | 16.88 | 0.49 | -0.08 | 0.76 | 0.26 |  |
| *Radix Trichosanthis* | MOL006758 | delta7-Stigmastenol-3-O-beta-D-glucoside | 576.95 | 6.34 | 4 | 6 | 20.67 | -0.19 | -0.98 | 0.63 | 0.22 |  |
| *Radix Trichosanthis* | MOL006759 | poriferast-7-en-3beta-ol | 414.79 | 8.08 | 1 | 1 | 8 | 1.45 | 1.08 | 0.75 | 0.21 |  |
| *Radix Trichosanthis* | MOL006760 | (R)-ornithine | 132.19 | -1.14 | 5 | 4 | 90.5 | -0.85 | -1.78 | 0.01 | 0.27 | 11.23 |
| *Radix Trichosanthis* | MOL006761 | Schottenol glucoside | 576.95 | 6.34 | 4 | 6 | 20.67 | -0.34 | -1.22 | 0.62 | 0.24 |  |
| *Radix Trichosanthis* | MOL006762 | 2-Hydroxymethylserine | 135.14 | -2.17 | 5 | 5 | 95.7 | -1.46 | -3.73 | 0.02 | 0.26 | 11.41 |
| *Poria Cocos* | MOL000273 | (2R)-2-[(3S,5R,10S,13R,14R,16R,17R)-3,16-dihydroxy-4,4,10,13,14-pentamethyl-2,3,5,6,12,15,16,17-octahydro-1H-cyclopenta[a]phenanthren-17-yl]-6-methylhept-5-enoic acid | 470.76 | 5.41 | 3 | 4 | 30.93 | 0.01 | -0.76 | 0.81 | 0 | 6.81 |
| *Poria Cocos* | MOL000274 | 3β-hydroxylanosta-7,9(11),24-trien-21-oic acid | 454.76 | 6.58 | 2 | 3 | 24.92 | 0.53 | -0.24 | 0.8 | 0 |  |
| *Poria Cocos* | MOL000275 | trametenolic acid | 456.78 | 7.03 | 2 | 3 | 38.71 | 0.52 | -0.14 | 0.8 | 0 | 7.78 |
| *Poria Cocos* | MOL000276 | 7,9(11)-dehydropachymic acid | 526.83 | 6.1 | 2 | 5 | 35.11 | 0.03 | -0.87 | 0.81 | 0 | 7.34 |
| *Poria Cocos* | MOL000277 | tumulosic acid | 486.81 | 6.16 | 3 | 4 | 15.95 | 0.02 | -0.51 | 0.81 | 0 |  |
| *Poria Cocos* | MOL000278 | Beta-Glucan | 516.56 | -4.88 | 9 | 15 | 0.73 | -3.13 | -7.78 | 0.7 | 0 |  |
| *Poria Cocos* | MOL000279 | Cerevisterol | 430.74 | 5.15 | 3 | 3 | 37.96 | 0.28 | -0.39 | 0.77 | 0 | 5.31 |
| *Poria Cocos* | MOL000280 | (2R)-2-[(3S,5R,10S,13R,14R,16R,17R)-3,16-dihydroxy-4,4,10,13,14-pentamethyl-2,3,5,6,12,15,16,17-octahydro-1H-cyclopenta[a]phenanthren-17-yl]-5-isopropyl-hex-5-enoic acid | 484.79 | 5.72 | 3 | 4 | 31.07 | 0.05 | -0.7 | 0.82 | 0 | 7.42 |
| *Poria Cocos* | MOL000281 | Dimethyl L-malate | 162.16 | -0.45 | 1 | 5 | 8.59 | -0.05 | -0.61 | 0.03 | 0 |  |
| *Poria Cocos* | MOL000282 | ergosta-7,22E-dien-3beta-ol | 398.74 | 7.18 | 1 | 1 | 43.51 | 1.32 | 0.91 | 0.72 | 0 | 5.11 |
| *Poria Cocos* | MOL000283 | Ergosterol peroxide | 430.74 | 7.17 | 1 | 3 | 40.36 | 0.84 | 0.34 | 0.81 | 0 | 3.43 |
| *Poria Cocos* | MOL000284 | L-uridine | 244.23 | -2.45 | 4 | 8 | 23.4 | -1.07 | -1.42 | 0.11 | 0 |  |
| *Poria Cocos* | MOL000285 | (2R)-2-[(5R,10S,13R,14R,16R,17R)-16-hydroxy-3-keto-4,4,10,13,14-pentamethyl-1,2,5,6,12,15,16,17-octahydrocyclopenta[a]phenanthren-17-yl]-5-isopropyl-hex-5-enoic acid | 482.77 | 5.68 | 2 | 4 | 38.26 | 0.12 | -0.57 | 0.82 | 0 | 6.77 |
| *Poria Cocos* | MOL000286 | β-amyrin acetate | 468.84 | 7.68 | 0 | 2 | 9.11 | 1.42 | 1.21 | 0.74 | 0 |  |
| *Poria Cocos* | MOL000287 | 3beta-Hydroxy-24-methylene-8-lanostene-21-oic acid | 470.81 | 7.33 | 2 | 3 | 38.7 | 0.61 | -0.04 | 0.81 | 0 | 6.59 |
| *Poria Cocos* | MOL000288 | pachyman | 500.56 | -4.27 | 9 | 14 | 0.45 | -2.36 | -3.58 | 0.68 | 0 |  |
| *Poria Cocos* | MOL000289 | pachymic acid | 528.85 | 6.54 | 2 | 5 | 33.63 | 0.1 | -0.57 | 0.81 | 0 | 9.27 |
| *Poria Cocos* | MOL000290 | Poricoic acid A | 498.77 | 5.94 | 3 | 5 | 30.61 | -0.14 | -0.93 | 0.76 | 0 | 8.26 |
| *Poria Cocos* | MOL000291 | Poricoic acid B | 484.74 | 5.64 | 3 | 5 | 30.52 | -0.08 | -0.87 | 0.75 | 0 | 8.67 |
| *Poria Cocos* | MOL000292 | poricoic acid C | 482.77 | 7.11 | 2 | 4 | 38.15 | 0.32 | -0.41 | 0.75 | 0 | 7.73 |
| *Poria Cocos* | MOL000293 | poricoic acid D | 514.77 | 4.73 | 4 | 6 | 22.38 | -0.62 | -1.29 | 0.78 | 0 |  |
| *Poria Cocos* | MOL000294 | poricoic acid DM | 528.8 | 4.98 | 3 | 6 | 29.32 | -0.34 | -0.94 | 0.78 | 0 |  |
| *Poria Cocos* | MOL000295 | alexandrin | 576.95 | 6.34 | 4 | 6 | 20.63 | -0.2 | -0.81 | 0.63 | 0 |  |
| *Poria Cocos* | MOL000296 | hederagenin | 414.79 | 8.08 | 1 | 1 | 36.91 | 1.32 | 0.96 | 0.75 | 0 | 5.35 |
| *Poria Cocos* | MOL000297 | Tumulosic acid | 486.81 | 6.16 | 3 | 4 | 29.88 | 0.13 | -0.47 | 0.81 | 0 |  |
| *Poria Cocos* | MOL000298 | ergosterol | 396.72 | 6.93 | 1 | 1 | 14.29 | 1.47 | 1.04 | 0.72 | 0 |  |
| *Poria Cocos* | MOL000299 | Trimethyl citrate | 234.23 | -0.64 | 1 | 7 | 67.61 | -0.06 | -0.33 | 0.07 | 0 | 6.28 |
| *Poria Cocos* | MOL000300 | dehydroeburicoic acid | 453.75 | 6.35 | 1 | 3 | 44.17 | 0.38 | -0.16 | 0.83 | 0.04 | 7.04 |
| *Poria Cocos* | MOL000301 | 2-lauroleic acid | 198.34 | 4.52 | 1 | 2 | 31.42 | 1.03 | 1.08 | 0.04 | 0 | 5.69 |
| *Poria Cocos* | MOL000302 | Undekansaeure | 186.33 | 4.09 | 1 | 2 | 30.14 | 0.98 | 0.94 | 0.03 | 0 | 4.93 |
| *Poria Cocos* | MOL000303 | caprylic acid | 144.24 | 2.72 | 1 | 2 | 16.4 | 0.9 | 1.02 | 0.02 | 0 |  |
| *Poria Cocos* | MOL000304 | Ethyl glucoside | 208.24 | -1.76 | 4 | 6 | 15.21 | -1.03 | -3.15 | 0.06 | 0 |  |
| *Poria Cocos* | MOL000305 | lauric acid | 200.36 | 4.54 | 1 | 2 | 23.59 | 1.02 | 1.1 | 0.04 | 0 |  |
| *Poria Cocos* | MOL000069 | palmitic acid | 256.48 | 6.37 | 1 | 2 | 19.3 | 1.09 | 1 | 0.1 | 0 |  |
| *Exocarpium Citri Grandis* | MOL000010 | Rhoifolin | 578.57 | -0.43 | 8 | 14 | 6.68 | -1.87 | -2.98 | 0.77 | 0.29 |  |
| *Exocarpium Citri Grandis* | MOL010267 | LYC | 536.96 | 12.76 | 0 | 0 | 32.57 | 2.41 | 1.45 | 0.51 | 0.37 | 3.42 |
| *Exocarpium Citri Grandis* | MOL010856 | putrescine | 88.18 | -0.83 | 4 | 2 | 81.23 | -0.08 | -0.92 | 0 | 0.16 | 11.02 |
| *Exocarpium Citri Grandis* | MOL011563 | gamma-Hexenol | 100.18 | 1.44 | 1 | 1 | 57.32 | 1.17 | 1.4 | 0.01 | 0.21 | 11.54 |
| *Exocarpium Citri Grandis* | MOL000123 | geraniol | 154.28 | 2.93 | 1 | 1 | 23.93 | 1.19 | 1.14 | 0.02 | 0.27 |  |
| *Exocarpium Citri Grandis* | MOL000127 | Neral | 152.26 | 3.19 | 0 | 1 | 19.48 | 1.36 | 1.51 | 0.02 | 0.34 |  |
| *Exocarpium Citri Grandis* | MOL013276 | poncirin | 594.62 | -0.21 | 7 | 14 | 36.55 | -1.67 | -2.59 | 0.74 | 0.28 | 14.83 |
| *Exocarpium Citri Grandis* | MOL013277 | Isosinensetin | 372.4 | 3.06 | 0 | 7 | 51.15 | 1.16 | 0.03 | 0.44 | 0.14 | 15.84 |
| *Exocarpium Citri Grandis* | MOL013278 | 4',5,7,8-Tetramethoxyflavone | 342.37 | 3.07 | 0 | 6 | 23.45 | 1.03 | 0.1 | 0.36 | 0.18 |  |
| *Exocarpium Citri Grandis* | MOL013279 | 5,7,4'-Trimethylapigenin | 312.34 | 3.09 | 0 | 5 | 39.83 | 1.01 | 0.12 | 0.3 | 0.22 | 15.85 |
| *Exocarpium Citri Grandis* | MOL001393 | myristic acid | 228.42 | 5.46 | 1 | 2 | 21.18 | 1.07 | 0.99 | 0.07 | 0.19 |  |
| *Exocarpium Citri Grandis* | MOL001396 | PENTADECYLIC ACID | 242.45 | 5.91 | 1 | 2 | 20.18 | 1.08 | 0.88 | 0.08 | 0.18 |  |
| *Exocarpium Citri Grandis* | MOL001641 | METHYL LINOLEATE | 294.53 | 6.64 | 0 | 2 | 41.93 | 1.44 | 1.08 | 0.17 | 0.21 | 6.05 |
| *Exocarpium Citri Grandis* | MOL000172 | Furol | 96.09 | 0.99 | 0 | 2 | 34.35 | 1.08 | 1.51 | 0.01 | 0.2 | 4.53 |
| *Exocarpium Citri Grandis* | MOL001797 | (2S)-7-[(2S,3R,4S,5S,6R)-4,5-dihydroxy-6-methylol-3-[(2S,3R,4R,5R,6S)-3,4,5-trihydroxy-6-methyl-tetrahydropyran-2-yl]oxy-tetrahydropyran-2-yl]oxy-5-hydroxy-2-(3-hydroxy-5-methoxy-phenyl)chroman-4-one | 610.62 | -0.48 | 8 | 15 | 11.17 | -2.17 | -3.01 | 0.7 | 0 |  |
| *Exocarpium Citri Grandis* | MOL001798 | neohesperidin_qt | 302.3 | 2.28 | 3 | 6 | 71.17 | 0.26 | -0.47 | 0.27 | 0 | 15.96 |
| *Exocarpium Citri Grandis* | MOL001803 | Sinensetin | 372.4 | 3.06 | 0 | 7 | 50.56 | 1.12 | 0.04 | 0.45 | 0.13 | 15.52 |
| *Exocarpium Citri Grandis* | MOL000193 | (Z)-caryophyllene | 204.39 | 4.75 | 0 | 0 | 30.29 | 1.82 | 2.15 | 0.09 | 0.28 | 8 |
| *Exocarpium Citri Grandis* | MOL001945 | Majudin | 216.2 | 2.19 | 0 | 4 | 42.21 | 0.94 | 0.69 | 0.13 | 0.21 | -3.6 |
| *Exocarpium Citri Grandis* | MOL000198 | (R)-linalool | 154.28 | 2.74 | 1 | 1 | 39.8 | 1.33 | 1.36 | 0.02 | 0.32 | 6.48 |
| *Exocarpium Citri Grandis* | MOL000234 | L-Limonen | 136.26 | 3.5 | 0 | 0 | 38.09 | 1.83 | 2.13 | 0.02 | 0.29 | 11.64 |
| *Exocarpium Citri Grandis* | MOL000254 | eugenol | 164.22 | 2.55 | 1 | 2 | 56.24 | 1.35 | 1.32 | 0.04 | 0.32 | 0.92 |
| *Exocarpium Citri Grandis* | MOL002558 | Skimmetin | 162.15 | 1.63 | 1 | 3 | 27.37 | 0.74 | 0.52 | 0.05 | 0.43 |  |
| *Exocarpium Citri Grandis* | MOL000269 | Elemicin | 208.28 | 2.79 | 0 | 3 | 21.94 | 1.41 | 1.28 | 0.06 | 0.2 |  |
| *Exocarpium Citri Grandis* | MOL003050 | nonanoic acid | 158.27 | 3.17 | 1 | 2 | 40.51 | 0.92 | 1.08 | 0.02 | 0.23 | 4.15 |
| *Exocarpium Citri Grandis* | MOL003534 | CADINENE | 204.39 | 4.75 | 0 | 0 | 17.12 | 1.88 | 2.06 | 0.08 | 0.25 |  |
| *Exocarpium Citri Grandis* | MOL000357 | Sitogluside | 576.95 | 6.34 | 4 | 6 | 20.63 | -0.14 | -0.93 | 0.62 | 0.23 |  |
| *Exocarpium Citri Grandis* | MOL000358 | beta-sitosterol | 414.79 | 8.08 | 1 | 1 | 36.91 | 1.32 | 0.99 | 0.75 | 0.23 | 5.36 |
| *Exocarpium Citri Grandis* | MOL003937 | Naphthalene, 1,2,3,4,4a,5,6,8a-octahydro-7-methyl-4-methylene-1-(1-methylethyl)-, (1alpha,4abeta,8aalpha)- | 204.39 | 4.8 | 0 | 0 | 20.21 | 1.87 | 2.06 | 0.08 | 0.26 |  |
| *Exocarpium Citri Grandis* | MOL004011 | Nevoli oil | 151.18 | 0.94 | 2 | 3 | 86.52 | 0.44 | 0.47 | 0.03 | 0.35 | -1.86 |
| *Exocarpium Citri Grandis* | MOL000421 | nicotinic acid | 123.12 | 0.28 | 1 | 3 | 47.65 | 0.34 | 0.21 | 0.02 | 0 | 11.98 |
| *Exocarpium Citri Grandis* | MOL004328 | naringenin | 272.27 | 2.3 | 3 | 5 | 59.29 | 0.28 | -0.37 | 0.21 | 0.4 | 16.98 |
| *Exocarpium Citri Grandis* | MOL000447 | stachydrine | 144.22 | 0.57 | 1 | 2 | 0.27 | 0.74 | 0.89 | 0.03 | 0.12 |  |
| *Exocarpium Citri Grandis* | MOL000475 | anethole | 148.22 | 2.77 | 0 | 1 | 32.49 | 1.75 | 1.81 | 0.03 | 0 | 1.68 |
| *Exocarpium Citri Grandis* | MOL000514 | Nonacosane | 408.89 | 13.6 | 0 | 0 | 8.12 | 1.92 | 1.54 | 0.39 | 0.16 |  |
| *Exocarpium Citri Grandis* | MOL002003 | (-)-Caryophyllene oxide | 220.39 | 3.52 | 0 | 1 | 32.67 | 1.58 | 1.76 | 0.13 | 0.28 | 6.51 |
| *Exocarpium Citri Grandis* | MOL005812 | naringin | 580.59 | -0.46 | 8 | 14 | 6.92 | -1.99 | -3 | 0.78 | 0.31 |  |
| *Exocarpium Citri Grandis* | MOL005814 | tangeretin | 372.4 | 3.06 | 0 | 7 | 21.38 | 1.23 | 0.09 | 0.43 | 0.16 |  |
| *Exocarpium Citri Grandis* | MOL005828 | nobiletin | 402.43 | 3.04 | 0 | 8 | 61.67 | 1.05 | -0.08 | 0.52 | 0.13 | 16.2 |
| *Exocarpium Citri Grandis* | MOL005849 | didymin | 286.3 | 2.55 | 2 | 5 | 38.55 | 0.6 | -0.07 | 0.24 | 0 | 16.86 |
| *Exocarpium Citri Grandis* | MOL000069 | palmitic acid | 256.48 | 6.37 | 1 | 2 | 19.3 | 1.09 | 1 | 0.1 | 0 |  |
| *Exocarpium Citri Grandis* | MOL000008 | apigenin | 270.25 | 2.33 | 3 | 5 | 23.06 | 0.43 | -0.61 | 0.21 | 0.41 |  |
| *Exocarpium Citri Grandis* | MOL000879 | methyl palmitate | 270.51 | 6.62 | 0 | 2 | 18.09 | 1.37 | 1.18 | 0.12 | 0.14 |  |
| *Exocarpium Citri Grandis* | MOL000089 | catechol | 110.12 | 1.3 | 2 | 2 | 29.86 | 1.09 | 1.32 | 0.02 | 0.42 |  |
| *Platycodon Grandiflora* | MOL001551 | Trochol | 442.8 | 6.31 | 2 | 2 | 15.48 | 0.84 | 0.35 | 0.78 | 0.24 |  |
| *Platycodon Grandiflora* | MOL001689 | acacetin | 284.28 | 2.59 | 2 | 5 | 34.97 | 0.67 | -0.05 | 0.24 | 0.35 | 17.25 |
| *Platycodon Grandiflora* | MOL001691 | vitamin c | 176.14 | -1.76 | 4 | 6 | 13.34 | -0.86 | -1.38 | 0.04 | 0.37 |  |
| *Platycodon Grandiflora* | MOL002378 | UND | 156.35 | 5.39 | 0 | 0 | 17.15 | 1.79 | 2.02 | 0.02 | 0.18 |  |
| *Platycodon Grandiflora* | MOL002778 | THZ | 135.2 | 1.91 | 0 | 1 | 70.33 | 1.39 | 1.51 | 0.03 | 0.25 | 13.39 |
| *Platycodon Grandiflora* | MOL002998 | IPH | 94.12 | 1.56 | 1 | 1 | 36.05 | 1.5 | 1.86 | 0.01 | 0.44 | 12.07 |
| *Platycodon Grandiflora* | MOL000421 | nicotinic acid | 123.12 | 0.28 | 1 | 3 | 47.65 | 0.34 | 0.21 | 0.02 | 0 | 11.98 |
| *Platycodon Grandiflora* | MOL004355 | Spinasterol | 412.77 | 7.64 | 1 | 1 | 42.98 | 1.44 | 1.04 | 0.76 | 0.21 | 5.32 |
| *Platycodon Grandiflora* | MOL004580 | cis-Dihydroquercetin | 304.27 | 1.49 | 5 | 7 | 66.44 | -0.34 | -1.11 | 0.27 | 0.4 | 14.51 |
| *Platycodon Grandiflora* | MOL005990 | 1-methoxy-1,2-butadiene | 84.13 | 0.83 | 0 | 1 | 52.77 | 1.4 | 1.7 | 0 | 0.35 | 11.78 |
| *Platycodon Grandiflora* | MOL005991 | ISOPROPYL FORMATE | 88.12 | 0.7 | 0 | 2 | 30.95 | 0.99 | 1.28 | 0 | 0.35 | 11.48 |
| *Platycodon Grandiflora* | MOL005992 | 2,3-dimethyl-1-pentene | 98.21 | 2.96 | 0 | 0 | 32.08 | 1.79 | 2.21 | 0.01 | 0.3 | 11.51 |
| *Platycodon Grandiflora* | MOL005993 | 2-cyclopentene-1-undecanoic acid | 252.44 | 5.35 | 1 | 2 | 14.33 | 1.11 | 1.05 | 0.11 | 0.22 |  |
| *Platycodon Grandiflora* | MOL005994 | 2-hydroxybicyclo[3.1.1]heptan-6-one | 126.17 | 0.17 | 1 | 2 | 79.61 | 0.27 | 0.42 | 0.04 | 0.28 | 11.04 |
| *Platycodon Grandiflora* | MOL005995 | 2'-O-acetylPlatycodin D2 | 520.78 | 2.17 | 6 | 7 | 10.19 | -1.05 | -1.89 | 0.67 | 0.23 |  |
| *Platycodon Grandiflora* | MOL005996 | 2-O-methyl-3―O-β-D-glucopyranosyl platycogenate A | 739.01 | 3.05 | 5 | 13 | 45.15 | -1.46 | -2.1 | 0.25 | 0.21 | 6.03 |
| *Platycodon Grandiflora* | MOL005997 | 2'-O-Polygalacin D | 1237.5 | -2.83 | 15 | 28 | 7.88 | -4.52 | -5.46 | 0.01 | 0.25 |  |
| *Platycodon Grandiflora* | MOL005998 | 2'-O-polygalacin D2 | 1385.62 | -4.41 | 18 | 33 | 7.57 | -5.81 | -6.89 | 0 | 0.26 |  |
| *Platycodon Grandiflora* | MOL005999 | crotonaldehyde | 70.1 | 0.92 | 0 | 1 | 64.99 | 1.15 | 1.61 | 0 | 0.38 | 12.09 |
| *Platycodon Grandiflora* | MOL000006 | luteolin | 286.25 | 2.07 | 4 | 6 | 36.16 | 0.19 | -0.84 | 0.25 | 0.39 | 15.94 |
| *Platycodon Grandiflora* | MOL006000 | Cyclopentenone | 82.11 | 0.7 | 0 | 1 | 13.06 | 1.14 | 1.54 | 0.01 | 0.38 |  |
| *Platycodon Grandiflora* | MOL006001 | Isobutenol | 72.12 | 0.71 | 1 | 1 | 28.16 | 1.04 | 1.26 | 0 | 0.3 |  |
| *Platycodon Grandiflora* | MOL006002 | 3,4-Heptadiene | 96.19 | 2.96 | 0 | 0 | 32.24 | 1.83 | 2.06 | 0.01 | 0.34 | 11.26 |
| *Platycodon Grandiflora* | MOL006003 | (1S,5R)-3,7,7-trimethylbicyclo[3.1.1]hept-3-ene | 136.26 | 2.87 | 0 | 0 | 51.75 | 1.81 | 2.18 | 0.05 | 0.25 | 11.21 |
| *Platycodon Grandiflora* | MOL006004 | 3'-a-O-Polygalacin D | 1237.5 | -2.83 | 15 | 28 | 7.72 | -4.64 | -5.57 | 0.01 | 0.24 |  |
| *Platycodon Grandiflora* | MOL006005 | allyloxyethylene | 84.13 | 0.75 | 0 | 1 | 48.91 | 1.41 | 1.72 | 0 | 0.36 | 11.74 |
| *Platycodon Grandiflora* | MOL006006 | 3-ethyl-1,4-hexadiene | 110.22 | 2.98 | 0 | 0 | 53.77 | 1.85 | 2.15 | 0.01 | 0.31 | 11.6 |
| *Platycodon Grandiflora* | MOL006007 | 3-ethyl-cyclohexene | 110.22 | 3 | 0 | 0 | 44.87 | 1.82 | 2.22 | 0.01 | 0.23 | 11.14 |
| *Platycodon Grandiflora* | MOL006008 | 3'-O-acetylPlatycodin D2 | 1401.62 | -5.5 | 19 | 34 | 7.57 | -6.1 | -6.79 | 0 | 0.27 |  |
| *Platycodon Grandiflora* | MOL006009 | 3'-O-acetylPlatycodin D2_qt | 520.78 | 2.17 | 6 | 7 | 7.61 | -0.76 | -1.46 | 0.67 | 0.22 |  |
| *Platycodon Grandiflora* | MOL006010 | 3-O-a-D-glucopyranosylplatycodigenin methyl ester | 696.97 | 0.68 | 8 | 12 | 3.72 | -2.08 | -2.87 | 0.29 | 0.23 |  |
| *Platycodon Grandiflora* | MOL006011 | 3-O-a-D-glucopyranosylplatycodigenin methyl ester_qt | 534.81 | 2.42 | 5 | 7 | 11.08 | -0.78 | -1.58 | 0.65 | 0.22 |  |
| *Platycodon Grandiflora* | MOL006012 | 3-O-a-gentiobiosylplatycodigenin methyl ester | 859.13 | -1.07 | 11 | 17 | 1.99 | -2.8 | -3.66 | 0.11 | 0.23 |  |
| *Platycodon Grandiflora* | MOL006013 | 3-O-a-Laminaribiosylplatycodigenin methyl ester | 859.13 | -1.07 | 11 | 17 | 2.89 | -2.93 | -3.89 | 0.11 | 0.23 |  |
| *Platycodon Grandiflora* | MOL006014 | 3-O-laminaribiosylplatycodigenin methyl ester | 861.15 | -0.82 | 11 | 17 | 2.58 | -2.85 | -3.68 | 0.11 | 0.18 |  |
| *Platycodon Grandiflora* | MOL006015 | 3-O-laminaribiosylplatycodigenin methyl ester_qt | 536.83 | 2.67 | 5 | 7 | 21.86 | -0.51 | -1.38 | 0.65 | 0.22 |  |
| *Platycodon Grandiflora* | MOL006016 | 3'-O-polygalacin D | 1385.62 | -4.41 | 18 | 33 | 7.58 | -5.86 | -6.85 | 0 | 0.27 |  |
| *Platycodon Grandiflora* | MOL006017 | 3-O-β-D-glucopyranosyl platycodigenin methyl ester | 696.97 | 0.68 | 8 | 12 | 2.83 | -1.73 | -2.34 | 0.29 | 0.22 |  |
| *Platycodon Grandiflora* | MOL006018 | dimethyl 3-o-β-D glucopyranosylplatycogenate A | 1221.5 | -4 | 16 | 27 | 7.61 | -5.94 | -7.1 | 0.01 | 0.24 |  |
| *Platycodon Grandiflora* | MOL006019 | 3-O-β-gentiobiosyl platycodigenin methylester | 859.13 | -1.07 | 11 | 17 | 13.5 | -3.25 | -3.82 | 0.11 | 0.22 |  |
| *Platycodon Grandiflora* | MOL006020 | 5-HEXENOIC ACID | 114.16 | 1.42 | 1 | 2 | 18.67 | 0.81 | 0.99 | 0.01 | 0.37 |  |
| *Platycodon Grandiflora* | MOL006021 | 16-oxo-platycodin D | 1223.47 | -4.51 | 16 | 28 | 7.84 | -4.93 | -6.24 | 0.01 | 0.25 |  |
| *Platycodon Grandiflora* | MOL006022 | α-Spinasterol-β-D-glucoside | 560.9 | 6.06 | 4 | 6 | 21.31 | -0.17 | -0.87 | 0.67 | 0.23 |  |
| *Platycodon Grandiflora* | MOL006023 | α-Spinasterol-β-D-glucoside_qt | 412.77 | 7.64 | 1 | 1 | 7.14 | 1.35 | 0.97 | 0.76 | 0.22 |  |
| *Platycodon Grandiflora* | MOL006024 | Deapio-Platycodin-D | 1093.36 | -3.06 | 15 | 24 | 2.85 | -4.71 | -5.87 | 0.03 | 0.24 |  |
| *Platycodon Grandiflora* | MOL006025 | Deapio-Platycodin-D3 | 1241.49 | -4.64 | 18 | 29 | 5.88 | -5.41 | -6.18 | 0.01 | 0.22 |  |
| *Platycodon Grandiflora* | MOL006026 | dimethyl 2-O-methyl-3-O-a-D-glucopyranosyl platycogenate A | 739.01 | 1.54 | 6 | 13 | 39.21 | -1.46 | -2.19 | 0.25 | 0.23 | 5.04 |
| *Platycodon Grandiflora* | MOL006027 | dimethyl 3-O-a-D-glucopyranosylplatycogenate A | 724.98 | 1.14 | 7 | 13 | 9.71 | -1.73 | -2.38 | 0.26 | 0.23 |  |
| *Platycodon Grandiflora* | MOL006028 | dimethyl 3-O-β-D-glucopyranosylplatycogenate A | 546.82 | 4.18 | 4 | 7 | 17.43 | -0.32 | -0.95 | 0.62 | 0.27 |  |
| *Platycodon Grandiflora* | MOL006029 | dimethyl platyconate A | 562.82 | 2.88 | 4 | 8 | 13.83 | -0.56 | -1.4 | 0.59 | 0.2 |  |
| *Platycodon Grandiflora* | MOL006030 | Flavoplatycoside | 612.59 | -1.28 | 10 | 16 | 5.77 | -2.6 | -3.55 | 0.66 | 0.33 |  |
| *Platycodon Grandiflora* | MOL006031 | Grandoside | 412.49 | -2.27 | 7 | 11 | 2.3 | -1.8 | -2.47 | 0.39 | 0.21 |  |
| *Platycodon Grandiflora* | MOL006032 | Inulin | 1113.16 | -11.16 | 18 | 33 | 3.01 | -5.81 | -7.23 | 0.14 | 0.23 |  |
| *Platycodon Grandiflora* | MOL006033 | latycodigenin | 520.78 | 2.17 | 6 | 7 | 7.43 | -0.75 | -1.45 | 0.67 | 0.21 |  |
| *Platycodon Grandiflora* | MOL006034 | lobetyol | 234.32 | 2.07 | 3 | 3 | 14.6 | -0.11 | -1.37 | 0.08 | 0.37 |  |
| *Platycodon Grandiflora* | MOL006035 | lobetyolin | 396.48 | 0.32 | 6 | 8 | 18.81 | -1.24 | -2.5 | 0.35 | 0.33 |  |
| *Platycodon Grandiflora* | MOL006036 | lobetyolinin | 556.67 | -1.22 | 9 | 12 | 6.08 | -2.27 | -3.6 | 0.67 | 0.31 |  |
| *Platycodon Grandiflora* | MOL006037 | lobetyolinin_qt | 234.32 | 2.07 | 3 | 3 | 31.16 | -0.1 | -1.16 | 0.08 | 0.36 | 3.85 |
| *Platycodon Grandiflora* | MOL006038 | Methy 2-O-methylPlatyconate A | 1251.53 | -2.73 | 15 | 28 | 8.04 | -4.39 | -5.49 | 0.01 | 0.25 |  |
| *Platycodon Grandiflora* | MOL006039 | Methyl 3-methyl-2-pentenoate | 128.19 | 2.02 | 0 | 2 | 32.85 | 1.21 | 1.43 | 0.01 | 0.25 | 11.25 |
| *Platycodon Grandiflora* | MOL006040 | methyl 3-O-β-D-glucopyranosyl polygalacate | 680.97 | 1.77 | 7 | 11 | 3.73 | -1.23 | -1.95 | 0.3 | 0.23 |  |
| *Platycodon Grandiflora* | MOL006041 | methyl 3-O-β-D-glucopyranosyl polygalacate _qt | 518.81 | 3.51 | 4 | 6 | 18.05 | -0.51 | -1.42 | 0.67 | 0.22 |  |
| *Platycodon Grandiflora* | MOL006042 | methyl 3-O-β-laminaribiosyl polygalacate | 859.18 | 0.2 | 10 | 16 | 3.4 | -2.6 | -3.42 | 0.11 | 0.21 |  |
| *Platycodon Grandiflora* | MOL006043 | methyl platyconate- A | 1121.37 | -2.78 | 14 | 25 | 8.47 | -4.01 | -4.98 | 0.03 | 0.24 |  |
| *Platycodon Grandiflora* | MOL006044 | methyl (4aR,5R,6aR,6aS,6bR,8aR,9R,10R,11S,12aR,14bS)-5,10,11-trihydroxy-9-(hydroxymethyl)-2,2,6a,6b,9,12a-hexamethyl-1,3,4,5,6,6a,7,8,8a,10,11,12,13,14b-tetradecahydropicene-4a-carboxylate | 518.81 | 3.51 | 4 | 6 | 9.43 | -0.36 | -1.03 | 0.67 | 0.22 |  |
| *Platycodon Grandiflora* | MOL006045 | methyl3-O-a-D-Glucopyranosylpolygalacate | 680.97 | 1.77 | 7 | 11 | 4.95 | -1.39 | -2.16 | 0.3 | 0.24 |  |
| *Platycodon Grandiflora* | MOL006046 | methyl3-O-a-laminaribiosylpolygalacate | 843.13 | 0.02 | 10 | 16 | 3.32 | -2.81 | -3.58 | 0.11 | 0.24 |  |
| *Platycodon Grandiflora* | MOL006047 | methylbutyl-1,2-benzenedicarboxylate | 236.29 | 3.38 | 2 | 4 | 68.3 | 0.31 | -0.02 | 0.09 | 0 | 4.39 |
| *Platycodon Grandiflora* | MOL006048 | methyl (4aR,5R,6aR,6aS,6bR,8aR,10R,11S,12aR,14bS)-5,10,11-trihydroxy-9,9-bis(hydroxymethyl)-2,2,6a,6b,12a-pentamethyl-1,3,4,5,6,6a,7,8,8a,10,11,12,13,14b-tetradecahydropicene-4a-carboxylate | 534.81 | 2.42 | 5 | 7 | 18.66 | -0.48 | -1.35 | 0.65 | 0.21 |  |
| *Platycodon Grandiflora* | MOL006049 | Platycodigenin | 522.8 | 2.42 | 6 | 7 | 14.62 | -0.9 | -1.73 | 0.67 | 0.24 |  |
| *Platycodon Grandiflora* | MOL006050 | Platycodin D2 | 1359.58 | -5.88 | 20 | 33 | 7.57 | -6.13 | -7.07 | 0 | 0.28 |  |
| *Platycodon Grandiflora* | MOL006051 | Platycodin-D3 | 1373.61 | -6.05 | 20 | 33 | 7.57 | -6.33 | -7.32 | 0 | 0.26 |  |
| *Platycodon Grandiflora* | MOL006052 | Platycogenic acid A | 536.78 | 2.63 | 6 | 8 | 13.94 | -1.04 | -1.79 | 0.65 | 0.31 |  |
| *Platycodon Grandiflora* | MOL006053 | Platycogenic acid B | 536.78 | 2.62 | 6 | 8 | 5.77 | -0.91 | -1.71 | 0.64 | 0.32 |  |
| *Platycodon Grandiflora* | MOL006054 | Platycogenic acid C | 506.8 | 3.5 | 5 | 6 | 14.12 | -0.58 | -1.31 | 0.69 | 0.27 |  |
| *Platycodon Grandiflora* | MOL006055 | platyconate A | 1239.47 | -3.84 | 16 | 29 | 7.68 | -5.36 | -6.2 | 0.01 | 0.29 |  |
| *Platycodon Grandiflora* | MOL006056 | platyconate A_qt | 548.79 | 2.63 | 5 | 8 | 13.21 | -0.94 | -1.7 | 0.62 | 0.25 |  |
| *Platycodon Grandiflora* | MOL006057 | Platyconic acid-A Lactone | 1295.59 | -3.17 | 16 | 29 | 7.72 | -5.08 | -6.4 | 0.01 | 0.25 |  |
| *Platycodon Grandiflora* | MOL006058 | platycoside A | 1227.46 | -4.47 | 18 | 29 | 6.13 | -5.2 | -6.08 | 0.01 | 0.25 |  |
| *Platycodon Grandiflora* | MOL006059 | platycoside B | 1121.37 | -2.51 | 14 | 25 | 2.21 | -4.42 | -5.46 | 0.03 | 0.24 |  |
| *Platycodon Grandiflora* | MOL006060 | platycoside C | 1121.37 | -2.51 | 14 | 25 | 2.18 | -4.53 | -5.34 | 0.03 | 0.26 |  |
| *Platycodon Grandiflora* | MOL006061 | platycoside F | 1045.31 | -1.07 | 11 | 22 | 17.74 | -3.45 | -4.56 | 0.05 | 0.24 |  |
| *Platycodon Grandiflora* | MOL006062 | platycoside G1 | 1355.6 | -4.43 | 17 | 32 | 5.03 | -5.82 | -7.02 | 0.01 | 0.26 |  |
| *Platycodon Grandiflora* | MOL006063 | platycoside G3 | 1325.57 | -4.09 | 16 | 31 | 8 | -5.73 | -7.15 | 0.02 | 0.26 |  |
| *Platycodon Grandiflora* | MOL006064 | Platyeoside D | 1533.8 | -6.88 | 22 | 37 | 7.57 | -6.87 | -8.27 | 0 | 0.24 |  |
| *Platycodon Grandiflora* | MOL006065 | Platyeoside E | 1549.8 | -7.97 | 23 | 38 | 7.57 | -7.24 | -8.77 | 0 | 0.23 |  |
| *Platycodon Grandiflora* | MOL006066 | Polygalacic acid | 506.8 | 3.51 | 5 | 6 | 20.07 | -0.66 | -1.46 | 0.7 | 0.26 |  |
| *Platycodon Grandiflora* | MOL006067 | Polygalacin D | 1195.46 | -3.21 | 16 | 27 | 7.6 | -5.61 | -6.5 | 0.01 | 0.26 |  |
| *Platycodon Grandiflora* | MOL006068 | polygalacin D2 | 1343.59 | -4.79 | 19 | 32 | 7.56 | -5.91 | -6.92 | 0.01 | 0.26 |  |
| *Platycodon Grandiflora* | MOL006069 | stigmastenol | 412.77 | 7.64 | 1 | 1 | 7.07 | 1.32 | 0.91 | 0.76 | 0.25 |  |
| *Platycodon Grandiflora* | MOL006070 | robinin | 592.6 | -0.18 | 7 | 14 | 39.84 | -1.48 | -2.36 | 0.71 | 0.3 | 16.67 |
| *Platycodon Grandiflora* | MOL006071 | (1R,2R,3R,5R)-5-(((1R,2R,4R,5S)-5-hydroxy-4-(hydroxymethyl)-2-(isopentylperoxy)cyclohexyl)oxy)-3-(hydroxymethyl)cyclohexane-1,2-diol | 392.55 | 0.29 | 5 | 8 | 5.68 | -1.09 | -1.78 | 0.36 | 0.18 |  |
| *Platycodon Grandiflora* | MOL006072 | Platycodin A | 1145.49 | -0.18 | 12 | 23 | 1.66 | -3 | -3.82 | 0.02 | 0.21 |  |
| *Platycodon Grandiflora* | MOL006073 | Platycodin C | 1145.49 | -0.18 | 12 | 23 | 1.61 | -3.25 | -3.86 | 0.02 | 0.21 |  |
| *Platycodon Grandiflora* | MOL006074 | Platycodin D | 1225.49 | -4.47 | 17 | 28 | 7.6 | -4.99 | -6.23 | 0.01 | 0.24 |  |
| *Platycodon Grandiflora* | MOL006075 | deapio platycoside F | 961.23 | -1.83 | 13 | 20 | 1.65 | -3.84 | -4.95 | 0.06 | 0.24 |  |
| *Platycodon Grandiflora* | MOL006076 | (deapio platycodin E | 1415.71 | -5.95 | 21 | 33 | 7.5 | -6.5 | -7.9 | 0 | 0.24 |  |
| *Platycodon Grandiflora* | MOL006077 | Thiamine | 265.4 | -0.05 | 3 | 4 | 19.87 | -0.32 | -1.14 | 0.11 | 0.15 |  |
| *Platycodon Grandiflora* | MOL000067 | L-Valin | 117.17 | 0.24 | 3 | 3 | 53.33 | 0.04 | -0.14 | 0.01 | 0 | 11.34 |
| *Platycodon Grandiflora* | MOL000068 | L-Ile | 131.2 | 0.7 | 3 | 3 | 59.05 | 0.06 | -0.11 | 0.02 | 0 | 11.21 |
| *Platycodon Grandiflora* | MOL000069 | palmitic acid | 256.48 | 6.37 | 1 | 2 | 19.3 | 1.09 | 1 | 0.1 | 0 |  |
| *Platycodon Grandiflora* | MOL000860 | stearic acid | 284.54 | 7.28 | 1 | 2 | 17.83 | 1.15 | 1.22 | 0.14 | 0.19 |  |
