# Supplementary material for: Network Pharmacology Analysis of the Therapeutic Mechanisms Underlying Beimu-Gualou Formula Activity against Bronchiectasis with In Silico Molecular Docking Validation
Source: Evid Based Complement Alternat Med. 2021 Jan 5;2021:3656272. doi: 10.1155/2021/3656272 (PMC7803403; doi:10.1155/2021/3656272)
Supplement: Supplementary Materials — Supplementary Table 1: the chemical compounds of 6 herbs in BMGLF. Supplementary Table 2: the targets of BMGLF and bronchiectasis. Supplementary Table 3: the data of GO enrichment analysis. Supplementary Table 4: the data of KEGG pathway enrichment analysis. [file 3656272.f1.zip › 3656272.f1/Supplementary Table 2.docx]

| **The targets of BMGLF and bronchiectasis** | |
| --- | --- |
| **Category** | **Targets** |
| BMGLF | ABAT |
| BMGLF | GABRA1 |
| BMGLF | ADH1B |
| BMGLF | ADH1C |
| BMGLF | ADH1A |
| BMGLF | LYZ |
| BMGLF | MAOB |
| BMGLF | SLC7A7 |
| BMGLF | SCN5A |
| BMGLF | CHRM3 |
| BMGLF | ADRB2 |
| BMGLF | CHRM1 |
| BMGLF | PTGS1 |
| BMGLF | MTAP |
| BMGLF | PTGS2 |
| BMGLF | ADA |
| BMGLF | ADRB1 |
| BMGLF | ADRA2A |
| BMGLF | ADRA2C |
| BMGLF | CTRB1 |
| BMGLF | SLC6A3 |
| BMGLF | AKR1B1 |
| BMGLF | PLAU |
| BMGLF | MAOA |
| BMGLF | ADRA1A |
| BMGLF | ADRA1D |
| BMGLF | TNFSF15 |
| BMGLF | CYP1A1 |
| BMGLF | PRKCB |
| BMGLF | BTK |
| BMGLF | GFAP |
| BMGLF | IGF2 |
| BMGLF | SELP |
| BMGLF | RAC1 |
| BMGLF | MGAM |
| BMGLF | F3 |
| BMGLF | PGR |
| BMGLF | KCNH2 |
| BMGLF | HTR3A |
| BMGLF | RXRA |
| BMGLF | ADRA1B |
| BMGLF | NCOA2 |
| BMGLF | CHRM4 |
| BMGLF | CHRM2 |
| BMGLF | CHRNA2 |
| BMGLF | SLC6A4 |
| BMGLF | OPRM1 |
| BMGLF | BCL2 |
| BMGLF | BAX |
| BMGLF | CASP9 |
| BMGLF | JUN |
| BMGLF | CASP3 |
| BMGLF | CASP8 |
| BMGLF | PRKCA |
| BMGLF | PON1 |
| BMGLF | MAP2 |
| BMGLF | NR3C2 |
| BMGLF | SLC6A2 |
| BMGLF | LTA4H |
| BMGLF | ADRA2B |
| BMGLF | NR3C1 |
| BMGLF | AR |
| BMGLF | CTSD |
| BMGLF | RHO |
| BMGLF | IL10 |
| BMGLF | COL1A1 |
| BMGLF | SLC22A5 |
| BMGLF | PCYT1A |
| BMGLF | TK1 |
| BMGLF | PLA2G2E |
| BMGLF | NOS3 |
| BMGLF | TYR |
| BMGLF | CCL16 |
| BMGLF | FOS |
| BMGLF | FOSL2 |
| BMGLF | PPARG |
| BMGLF | SREBF2 |
| BMGLF | CYP1A2 |
| BMGLF | COL7A1 |
| BMGLF | NR1I3 |
| BMGLF | SREBF1 |
| BMGLF | GLUL |
| BMGLF | ALAD |
| BMGLF | SI |
| BMGLF | LCT |
| BMGLF | G6PC |
| BMGLF | AMY2A |
| BMGLF | AMY1A |
| BMGLF | CHI3L1 |
| BMGLF | HK1 |
| BMGLF | ACSL1 |
| BMGLF | ACSL4 |
| BMGLF | SP1 |
| BMGLF | ENPP7 |
| BMGLF | F7 |
| BMGLF | NOS2 |
| BMGLF | CHRM5 |
| BMGLF | OPRD1 |
| BMGLF | DRD2 |
| BMGLF | GRIA2 |
| BMGLF | ESR1 |
| BMGLF | TYRP1 |
| BMGLF | BCHE |
| BMGLF | PLA2G4F |
| BMGLF | NCOA1 |
| BMGLF | LCAT |
| BMGLF | ESR2 |
| BMGLF | MAPK14 |
| BMGLF | GSK3B |
| BMGLF | PRSS1 |
| BMGLF | SULT1E1 |
| BMGLF | RELA |
| BMGLF | AKT1 |
| BMGLF | IL6 |
| BMGLF | CXCL8 |
| BMGLF | DUOX2 |
| BMGLF | CD80 |
| BMGLF | CD86 |
| BMGLF | CD40 |
| BMGLF | ISYNA1 |
| BMGLF | AMD1 |
| BMGLF | KYNU |
| BMGLF | GOT1 |
| BMGLF | PYGM |
| BMGLF | GOT2 |
| BMGLF | PPAT |
| BMGLF | CTH |
| BMGLF | GATM |
| BMGLF | NFS1 |
| BMGLF | SLC36A1 |
| BMGLF | ME2 |
| BMGLF | ME3 |
| BMGLF | PRSS3 |
| BMGLF | GPT |
| BMGLF | AGXT2 |
| BMGLF | OAT |
| BMGLF | GLB1 |
| BMGLF | TPI1 |
| BMGLF | ACHE |
| BMGLF | RXRG |
| BMGLF | TRPV1 |
| BMGLF | PCNA |
| BMGLF | ALB |
| BMGLF | MYC |
| BMGLF | HNF4A |
| BMGLF | UCP2 |
| BMGLF | HNF1A |
| BMGLF | ACTB |
| BMGLF | TRIM26 |
| BMGLF | ALDH5A1 |
| BMGLF | MMP8 |
| BMGLF | MMP12 |
| BMGLF | GALE |
| BMGLF | ARG1 |
| BMGLF | CAT |
| BMGLF | RRM1 |
| BMGLF | F13A1 |
| BMGLF | ME1 |
| BMGLF | LDHA |
| BMGLF | GCAT |
| BMGLF | LDHB |
| BMGLF | CDC25B |
| BMGLF | ALDH2 |
| BMGLF | SHMT2 |
| BMGLF | SLC6A9 |
| BMGLF | LTF |
| BMGLF | PFAS |
| BMGLF | MTR |
| BMGLF | GLUD1 |
| BMGLF | PYGL |
| BMGLF | ASNS |
| BMGLF | CPT2 |
| BMGLF | SHMT1 |
| BMGLF | GARS |
| BMGLF | GNMT |
| BMGLF | GLYAT |
| BMGLF | NAGS |
| BMGLF | GPT2 |
| BMGLF | GLYATL1 |
| BMGLF | GPHN |
| BMGLF | PIPOX |
| BMGLF | SLC25A10 |
| BMGLF | GLUD2 |
| BMGLF | ATP2C1 |
| BMGLF | ADSSL1 |
| BMGLF | GLRA2 |
| BMGLF | PDHB |
| BMGLF | ALAS2 |
| BMGLF | GLRA3 |
| BMGLF | ALAS1 |
| BMGLF | GRIA1 |
| BMGLF | OPLAH |
| BMGLF | SLC25A13 |
| BMGLF | PC |
| BMGLF | SDHA |
| BMGLF | GPI |
| BMGLF | ALDH1A1 |
| BMGLF | OTC |
| BMGLF | SLC7A4 |
| BMGLF | PPIA |
| BMGLF | DAO |
| BMGLF | P4HA1 |
| BMGLF | MMP9 |
| BMGLF | MAPK1 |
| BMGLF | CKM |
| BMGLF | ALOX5 |
| BMGLF | P3H3 |
| BMGLF | SLC25A12 |
| BMGLF | NCF1 |
| BMGLF | SOD1 |
| BMGLF | TEP1 |
| BMGLF | EDN3 |
| BMGLF | ERBB2 |
| BMGLF | LPL |
| BMGLF | GAP43 |
| BMGLF | SERPINE1 |
| BMGLF | BDNF |
| BMGLF | HMGCR |
| BMGLF | MPO |
| BMGLF | PPARA |
| BMGLF | PPARD |
| BMGLF | CRP |
| BMGLF | INS |
| BMGLF | PLG |
| BMGLF | FABP1 |
| BMGLF | RBP2 |
| BMGLF | ENPEP |
| BMGLF | SOAT1 |
| BMGLF | CCK |
| BMGLF | CITED1 |
| BMGLF | NTRK2 |
| BMGLF | PDX1 |
| BMGLF | SLC2A2 |
| BMGLF | PAM |
| BMGLF | SCD |
| BMGLF | UCP3 |
| BMGLF | CETP |
| BMGLF | PYY |
| BMGLF | DNPEP |
| BMGLF | BCAT1 |
| BMGLF | PTGER3 |
| BMGLF | STAT3 |
| BMGLF | CCNB1 |
| BMGLF | VCP |
| BMGLF | SLC6A1 |
| BMGLF | NEU1 |
| BMGLF | MAN2A1 |
| BMGLF | ACPP |
| BMGLF | DHCR7 |
| BMGLF | EP300 |
| BMGLF | TLR2 |
| BMGLF | NFKB1 |
| BMGLF | POMC |
| BMGLF | OXTR |
| BMGLF | TGM2 |
| BMGLF | CCND1 |
| BMGLF | MAPK3 |
| BMGLF | BAK1 |
| BMGLF | CYP2B6 |
| BMGLF | CHEK1 |
| BMGLF | KDR |
| BMGLF | CYP1B1 |
| BMGLF | AHR |
| BMGLF | ABCC2 |
| BMGLF | PPP3CA |
| BMGLF | MUC1 |
| BMGLF | CRYZ |
| BMGLF | TRPC3 |
| BMGLF | TRPV3 |
| BMGLF | ENOX2 |
| BMGLF | APOB |
| BMGLF | ATP5F1B |
| BMGLF | HP |
| BMGLF | PDHX |
| BMGLF | MOGAT2 |
| BMGLF | PPARGC1A |
| BMGLF | FASN |
| BMGLF | LDLR |
| BMGLF | MTTP |
| BMGLF | PLB1 |
| BMGLF | GSTP1 |
| BMGLF | UGT1A1 |
| BMGLF | GSR |
| BMGLF | ABCC1 |
| BMGLF | ADIPOQ |
| BMGLF | SOAT2 |
| BMGLF | AKR1C1 |
| BMGLF | CES1 |
| BMGLF | NFKBIA |
| BMGLF | IL2 |
| BMGLF | CDKN1A |
| BMGLF | RASGRF2 |
| BMGLF | RAF1 |
| BMGLF | EIF6 |
| BMGLF | HMOX1 |
| BMGLF | TP63 |
| BMGLF | MAPK8 |
| BMGLF | TIMP1 |
| BMGLF | CREB1 |
| BMGLF | PLA2G4A |
| BMGLF | CD163 |
| BMGLF | EPHB2 |
| BMGLF | VEGFA |
| BMGLF | BCL2L1 |
| BMGLF | RB1 |
| BMGLF | EEF1E1 |
| BMGLF | AHSA1 |
| BMGLF | ODC1 |
| BMGLF | MDM2 |
| BMGLF | MMP1 |
| BMGLF | HIF1A |
| BMGLF | IGF1R |
| BMGLF | RUNX1T1 |
| BMGLF | ACACA |
| BMGLF | ICAM1 |
| BMGLF | MCL1 |
| BMGLF | CCND2 |
| BMGLF | IFNG |
| BMGLF | IL4 |
| BMGLF | IKBKG |
| BMGLF | PSMD3 |
| BMGLF | SLC2A4 |
| BMGLF | INSR |
| BMGLF | CD40LG |
| BMGLF | CYCS |
| BMGLF | CFLAR |
| BMGLF | AAGAB |
| BMGLF | FCER2 |
| BMGLF | IL13 |
| BMGLF | MS4A2 |
| BMGLF | ALPI |
| BMGLF | PSME3 |
| BMGLF | APC |
| BMGLF | TRPM2 |
| BMGLF | AKR1C3 |
| BMGLF | SLC5A5 |
| BMGLF | FXYD2 |
| BMGLF | ALG5 |
| BMGLF | COMT |
| BMGLF | MYB |
| BMGLF | FASLG |
| BMGLF | CSF2 |
| BMGLF | EGFR |
| BMGLF | MMP2 |
| BMGLF | TOP1 |
| BMGLF | APP |
| BMGLF | CASP7 |
| BMGLF | BIRC5 |
| BMGLF | TOP2A |
| BMGLF | PTGES |
| BMGLF | NUF2 |
| BMGLF | ADCY2 |
| BMGLF | MET |
| BMGLF | ADORA2A |
| BMGLF | PDE3A |
| BMGLF | PIK3CG |
| BMGLF | DRD1 |
| BMGLF | GABRA2 |
| BMGLF | HTR2A |
| BMGLF | GABRA5 |
| BMGLF | GABRA3 |
| BMGLF | TGFB1 |
| BMGLF | GUSBP1 |
| BMGLF | CA2 |
| BMGLF | DPP4 |
| BMGLF | HTR2C |
| BMGLF | GRIN1 |
| BMGLF | SRC |
| BMGLF | XDH |
| BMGLF | SDS |
| BMGLF | NOS1 |
| BMGLF | IKBKB |
| BMGLF | GLDC |
| BMGLF | DHODH |
| BMGLF | MARS |
| BMGLF | GRIK2 |
| BMGLF | ARG2 |
| BMGLF | MAT1A |
| BMGLF | GABRA6 |
| BMGLF | MAT2A |
| BMGLF | SLC7A1 |
| BMGLF | GCG |
| BMGLF | KCNMA1 |
| BMGLF | HTR1A |
| BMGLF | PTEN |
| bronchiectasis | SERPINA1 |
| bronchiectasis | TNF |
| bronchiectasis | ICAM1 |
| bronchiectasis | CFTR |
| bronchiectasis | TAP2 |
| bronchiectasis | TAP1 |
| bronchiectasis | STAT1 |
| bronchiectasis | DNAH11 |
| bronchiectasis | TNFRSF13B |
| bronchiectasis | SCNN1B |
| bronchiectasis | SCNN1G |
| bronchiectasis | IL21R |
| bronchiectasis | ZMYND10 |
| bronchiectasis | DNAI1 |
| bronchiectasis | RTEL1 |
| bronchiectasis | LRRC6 |
| bronchiectasis | ICOS |
| bronchiectasis | ATP11A |
| bronchiectasis | CCNO |
| bronchiectasis | FAM13A |
| bronchiectasis | TNFSF12 |
| bronchiectasis | CXCR4 |
| bronchiectasis | TERT |
| bronchiectasis | CCDC39 |
| bronchiectasis | TERC |
| bronchiectasis | CCDC151 |
| bronchiectasis | HYDIN |
| bronchiectasis | CCDC40 |
| bronchiectasis | TNFRSF13C |
| bronchiectasis | DNAAF1 |
| bronchiectasis | PIH1D3 |
| bronchiectasis | DNAAF4 |
| bronchiectasis | RSPH9 |
| bronchiectasis | RSPH4A |
| bronchiectasis | DNAAF3 |
| bronchiectasis | CCDC103 |
| bronchiectasis | SFTPA1 |
| bronchiectasis | MUC5B |
| bronchiectasis | CCDC114 |
| bronchiectasis | DRC1 |
| bronchiectasis | ARMC4 |
| bronchiectasis | CFAP298 |
| bronchiectasis | DNAI2 |
| bronchiectasis | STN1 |
| bronchiectasis | TTC25 |
| bronchiectasis | DNAL1 |
| bronchiectasis | RSPH3 |
| bronchiectasis | CCDC65 |
| bronchiectasis | RSPH1 |
| bronchiectasis | DPP9 |
| bronchiectasis | SFTPA2 |
| bronchiectasis | PARN |
| bronchiectasis | CD19 |
| bronchiectasis | CD81 |
| bronchiectasis | LRBA |
| bronchiectasis | NFKB2 |
| bronchiectasis | NFKB1 |
| bronchiectasis | CR2 |
| bronchiectasis | NBN |
| bronchiectasis | DNMT3B |
| bronchiectasis | DSP |
| bronchiectasis | GAS8 |
| bronchiectasis | IGHM |
| bronchiectasis | PGM3 |
| bronchiectasis | PIK3CD |
| bronchiectasis | ABCA3 |
| bronchiectasis | ATM |
| bronchiectasis | SPAG1 |
| bronchiectasis | B2M |
| bronchiectasis | SFTPC |
| bronchiectasis | SCNN1A |
| bronchiectasis | MS4A1 |
| bronchiectasis | BLM |
| bronchiectasis | TAPBP |
| bronchiectasis | PRKCD |
| bronchiectasis | CD8A |
| bronchiectasis | MBL2 |
| bronchiectasis | COPD |
| bronchiectasis | CXCL8 |
| bronchiectasis | HLA-C |
| bronchiectasis | IL23A |
| bronchiectasis | RMRP |
| bronchiectasis | CTSB |
| bronchiectasis | SEC14L2 |
| bronchiectasis | JMJD6 |
| bronchiectasis | NXF1 |
| bronchiectasis | NOS2 |
| bronchiectasis | TGFB1 |
| bronchiectasis | SSB |
| bronchiectasis | USO1 |
| bronchiectasis | TLR2 |
| bronchiectasis | SFTPD |
| bronchiectasis | TRIM21 |
| bronchiectasis | STAT3 |
| bronchiectasis | MT1E |
| bronchiectasis | MMP8 |
| bronchiectasis | ELN |
| bronchiectasis | ISYNA1 |
| bronchiectasis | IL17A |
| bronchiectasis | MMP1 |
| bronchiectasis | IL1B |
| bronchiectasis | IL1A |
| bronchiectasis | FOXP3 |
| bronchiectasis | CFI |
| bronchiectasis | MMP9 |
| bronchiectasis | HLA-B |
| bronchiectasis | MBL3P |
| bronchiectasis | FLNB |
| bronchiectasis | FCN2 |
| bronchiectasis | FCGRT |
| bronchiectasis | MICA |
| bronchiectasis | DNAH5 |
| bronchiectasis | DNAH1 |
| bronchiectasis | DNAAF2 |
| bronchiectasis | FBLN5 |
| bronchiectasis | ELANE |
| bronchiectasis | MCIDAS |
| bronchiectasis | CFAP300 |
| bronchiectasis | HLA-DRB1 |
| bronchiectasis | ALDH18A1 |
| bronchiectasis | NME8 |
| bronchiectasis | DNAAF5 |
| bronchiectasis | CD79A |
| bronchiectasis | PIK3R1 |
| bronchiectasis | MPO |
| bronchiectasis | CTSG |
| bronchiectasis | BPI |
| bronchiectasis | RIN2 |
| bronchiectasis | LTBR |
| bronchiectasis | LOC105369626 |
| bronchiectasis | BTK |
| bronchiectasis | LOC107984500 |
| bronchiectasis | MUCL3 |
| bronchiectasis | IFNG |
| bronchiectasis | SERPINA3 |
| bronchiectasis | ALB |
| bronchiectasis | MALT1 |
| bronchiectasis | CEP164 |
| bronchiectasis | DNAJB13 |
| bronchiectasis | PTEN |
| bronchiectasis | ZAP70 |
| bronchiectasis | STX1A |
| bronchiectasis | FCGR2A |
| bronchiectasis | DCTN4 |
| bronchiectasis | RECQL4 |
| bronchiectasis | CLCA4 |
| bronchiectasis | ADRB2 |
| bronchiectasis | ENSG00000284862 |
| bronchiectasis | IL6 |
| bronchiectasis | HLA-A |
| bronchiectasis | CD40 |
| bronchiectasis | RASSF1 |
| bronchiectasis | MRPS18A |
| bronchiectasis | BLNK |
| bronchiectasis | CD79B |
| bronchiectasis | IRF8 |
| bronchiectasis | TCF3 |
| bronchiectasis | IGLL1 |
| bronchiectasis | SLC29A3 |
| bronchiectasis | LRRC8A |
| bronchiectasis | BTNL2 |
| bronchiectasis | TLR9 |
| bronchiectasis | SLPI |
| bronchiectasis | TLR4 |
| bronchiectasis | CXCR1 |
| bronchiectasis | CD36 |
| bronchiectasis | LOC101930593 |
| bronchiectasis | SLC11A1 |
| bronchiectasis | UNG |
| bronchiectasis | IL2RA |
| bronchiectasis | RIPK1 |
| bronchiectasis | CXCL12 |
| bronchiectasis | ABCC6 |
| bronchiectasis | RFXANK |
| bronchiectasis | RFX5 |
| bronchiectasis | KLRC1 |
| bronchiectasis | RFXAP |
| bronchiectasis | CARMIL2 |
| bronchiectasis | LOC105378083 |
| bronchiectasis | CILD4 |
| bronchiectasis | LOC105375668 |
| bronchiectasis | LOC105375669 |
| bronchiectasis | CILD8 |
| bronchiectasis | SS3 |
| bronchiectasis | LTA |
| bronchiectasis | RNASE3 |
| bronchiectasis | HGF |
| bronchiectasis | IL12RB1 |
| bronchiectasis | IL4 |
| bronchiectasis | CD40LG |
| bronchiectasis | IL5 |
| bronchiectasis | HLA-DQB1 |
| bronchiectasis | HLA-DQA1 |
| bronchiectasis | IL10 |
| bronchiectasis | AK7 |
| bronchiectasis | MUC5AC |
| bronchiectasis | S100A9 |
| bronchiectasis | S100A8 |
| bronchiectasis | IFNGR1 |
| bronchiectasis | PYCR1 |
| bronchiectasis | WRN |
| bronchiectasis | IL2 |
| bronchiectasis | TNFSF13B |
| bronchiectasis | FBN1 |
| bronchiectasis | ATP6V0A2 |
| bronchiectasis | IL13 |
| bronchiectasis | IL9 |
| bronchiectasis | PRKCSH |
| bronchiectasis | NODAL |
| bronchiectasis | CCL17 |
| bronchiectasis | DNAH8 |
| bronchiectasis | RGL3 |
| bronchiectasis | FAM219A |
| bronchiectasis | LOC105371891 |
| bronchiectasis | LOC111674463 |
| bronchiectasis | CCL2 |
| bronchiectasis | IL18 |
| bronchiectasis | SPINK1 |
| bronchiectasis | SERPINH1 |
| bronchiectasis | SOD1 |
| bronchiectasis | AICDA |
| bronchiectasis | CXCR3 |
| bronchiectasis | VIP |
| bronchiectasis | MUC2 |
| bronchiectasis | CCL3 |
| bronchiectasis | FGFR1 |
| bronchiectasis | TP53 |
| bronchiectasis | CHEK2 |
| bronchiectasis | ATP2A2 |
| bronchiectasis | BRCA1 |
| bronchiectasis | TF |
| bronchiectasis | SNCA |
| bronchiectasis | KCNQ1 |
| bronchiectasis | TNFRSF1A |
| bronchiectasis | CHRNA4 |
| bronchiectasis | ABCC1 |
| bronchiectasis | SGK1 |
| bronchiectasis | RAD50 |
| bronchiectasis | TNNI3 |
| bronchiectasis | VCP |
| bronchiectasis | GAA |
| bronchiectasis | ATF6 |
| bronchiectasis | LEF1 |
| bronchiectasis | SLC9A3 |
| bronchiectasis | CLCN2 |
| bronchiectasis | ACLY |
| bronchiectasis | APEX1 |
| bronchiectasis | GUCY2C |
| bronchiectasis | PRKG2 |
| bronchiectasis | PRSS1 |
| bronchiectasis | HELLS |
| bronchiectasis | TARDBP |
| bronchiectasis | SLC9A3R1 |
| bronchiectasis | VDAC1 |
| bronchiectasis | CHRNA5 |
| bronchiectasis | CANX |
| bronchiectasis | CCR7 |
| bronchiectasis | P2RY2 |
| bronchiectasis | FOXC2 |
| bronchiectasis | CSF2 |
| bronchiectasis | DNAJC5 |
| bronchiectasis | BMP6 |
| bronchiectasis | POSTN |
| bronchiectasis | PSMB1 |
| bronchiectasis | PSMB4 |
| bronchiectasis | PRSS8 |
| bronchiectasis | PRTN3 |
| bronchiectasis | H2AFX |
| bronchiectasis | CCL5 |
| bronchiectasis | CLCN3 |
| bronchiectasis | DAB2 |
| bronchiectasis | NOL3 |
| bronchiectasis | QPCT |
| bronchiectasis | IL31RA |
| bronchiectasis | HSPH1 |
| bronchiectasis | UBC |
| bronchiectasis | EPX |
| bronchiectasis | CD80 |
| bronchiectasis | GOPC |
| bronchiectasis | SST |
| bronchiectasis | ERLIN1 |
| bronchiectasis | ERLIN2 |
| bronchiectasis | DNASE1 |
| bronchiectasis | CAMP |
| bronchiectasis | PLCZ1 |
| bronchiectasis | PSMB3 |
| bronchiectasis | RECQL |
| bronchiectasis | RNF5 |
| bronchiectasis | RAB11B |
| bronchiectasis | PSMD11 |
| bronchiectasis | ADAM28 |
| bronchiectasis | IFRD1 |
| bronchiectasis | PSMC4 |
| bronchiectasis | RECQL5 |
| bronchiectasis | SCGB1A1 |
| bronchiectasis | MRE11 |
| bronchiectasis | SLC9A3R2 |
| bronchiectasis | INSIG1 |
| bronchiectasis | CLCA1 |
| bronchiectasis | SOX18 |
| bronchiectasis | REG3A |
| bronchiectasis | LPO |
| bronchiectasis | CXCL9 |
| bronchiectasis | DEFB1 |
| bronchiectasis | PES1 |
| bronchiectasis | GUCA2A |
| bronchiectasis | TTC14 |
| bronchiectasis | DERL1 |
| bronchiectasis | DEFB4A |
| bronchiectasis | SCT |
| bronchiectasis | TCHH |
| bronchiectasis | ADGRL3 |
| bronchiectasis | CYS1 |
| bronchiectasis | GAS8-AS1 |
| bronchiectasis | URAHP |
| bronchiectasis | MIR1268B |
| bronchiectasis | CFTR-AS1 |
| bronchiectasis | CFM1 |
| bronchiectasis | LOC105375767 |
| bronchiectasis | LOC108491823 |
| bronchiectasis | LOC111674464 |
| bronchiectasis | LOC111674465 |
| bronchiectasis | LOC111674466 |
| bronchiectasis | LOC111674470 |
| bronchiectasis | LOC111674471 |
| bronchiectasis | LOC111674472 |
| bronchiectasis | LOC111674473 |
| bronchiectasis | LOC111674474 |
| bronchiectasis | LOC111674475 |
| bronchiectasis | LOC111674476 |
| bronchiectasis | LOC111674477 |
| bronchiectasis | LOC111674478 |
| bronchiectasis | LOC111674479 |
| bronchiectasis | LOC113523647 |
| bronchiectasis | SELL |
| bronchiectasis | ADA |
| bronchiectasis | EDN1 |
| bronchiectasis | CDH1 |
| bronchiectasis | CCL4 |
| bronchiectasis | SH2D1A |
| bronchiectasis | LIG4 |
| bronchiectasis | CTLA4 |
| bronchiectasis | MUC1 |
| bronchiectasis | RAG1 |
| bronchiectasis | DCLRE1C |
| bronchiectasis | TNFRSF8 |
| bronchiectasis | USB1 |
| bronchiectasis | LCK |
| bronchiectasis | TGFBR1 |
| bronchiectasis | DNMT1 |
| bronchiectasis | ALK |
| bronchiectasis | BLK |
| bronchiectasis | ATR |
| bronchiectasis | PCNA |
| bronchiectasis | MAPK1 |
| bronchiectasis | CDKN1A |
| bronchiectasis | FAS |
| bronchiectasis | PTPRC |
| bronchiectasis | PRKDC |
| bronchiectasis | SPARC |
| bronchiectasis | RAD51 |
| bronchiectasis | CHEK1 |
| bronchiectasis | GATA3 |
| bronchiectasis | PDGFB |
| bronchiectasis | ACE |
| bronchiectasis | AGT |
| bronchiectasis | ALOX5 |
| bronchiectasis | MMP7 |
| bronchiectasis | MIF |
| bronchiectasis | STAT6 |
| bronchiectasis | IL1RN |
| bronchiectasis | SMAD3 |
| bronchiectasis | CXCR2 |
| bronchiectasis | DDR1 |
| bronchiectasis | FASLG |
| bronchiectasis | LEP |
| bronchiectasis | PNP |
| bronchiectasis | NOD2 |
| bronchiectasis | IL2RB |
| bronchiectasis | FGF2 |
| bronchiectasis | TNFRSF1B |
| bronchiectasis | CD28 |
| bronchiectasis | FEN1 |
| bronchiectasis | CCR3 |
| bronchiectasis | CD3G |
| bronchiectasis | SMAD2 |
| bronchiectasis | TRPV1 |
| bronchiectasis | VAV1 |
| bronchiectasis | CD27 |
| bronchiectasis | ADIPOQ |
| bronchiectasis | IL1R1 |
| bronchiectasis | LIG1 |
| bronchiectasis | PML |
| bronchiectasis | SAG |
| bronchiectasis | PAX5 |
| bronchiectasis | PDE4A |
| bronchiectasis | LPAR1 |
| bronchiectasis | MECP2 |
| bronchiectasis | TIMP1 |
| bronchiectasis | SPP1 |
| bronchiectasis | VCAM1 |
| bronchiectasis | CD38 |
| bronchiectasis | FLNC |
| bronchiectasis | FHIT |
| bronchiectasis | CCR4 |
| bronchiectasis | C1QC |
| bronchiectasis | LIG3 |
| bronchiectasis | RPA1 |
| bronchiectasis | RPA2 |
| bronchiectasis | TMPO |
| bronchiectasis | EPHB6 |
| bronchiectasis | CD2 |
| bronchiectasis | CDC45 |
| bronchiectasis | CHIT1 |
| bronchiectasis | CXCL10 |
| bronchiectasis | CD86 |
| bronchiectasis | APTX |
| bronchiectasis | ATP6V1A |
| bronchiectasis | ATP6V1E1 |
| bronchiectasis | CBX5 |
| bronchiectasis | ARRB2 |
| bronchiectasis | INVS |
| bronchiectasis | SFTPB |
| bronchiectasis | PDGFA |
| bronchiectasis | NLRC4 |
| bronchiectasis | NLRP12 |
| bronchiectasis | IL21 |
| bronchiectasis | TNFSF13 |
| bronchiectasis | SMARCAL1 |
| bronchiectasis | TNFRSF17 |
| bronchiectasis | TOLLIP |
| bronchiectasis | UNC13D |
| bronchiectasis | EFEMP2 |
| bronchiectasis | CXCL1 |
| bronchiectasis | CYSLTR1 |
| bronchiectasis | AREG |
| bronchiectasis | CCL11 |
| bronchiectasis | SEL1L |
| bronchiectasis | NOX4 |
| bronchiectasis | LRP4 |
| bronchiectasis | TAC1 |
| bronchiectasis | IL3 |
| bronchiectasis | IL12B |
| bronchiectasis | IGFBP5 |
| bronchiectasis | TP53BP1 |
| bronchiectasis | VAMP7 |
| bronchiectasis | MS4A2 |
| bronchiectasis | FGF7 |
| bronchiectasis | CXCL5 |
| bronchiectasis | CD69 |
| bronchiectasis | CD70 |
| bronchiectasis | FOXN1 |
| bronchiectasis | FANCM |
| bronchiectasis | CHRDL1 |
| bronchiectasis | MDC1 |
| bronchiectasis | LTBP4 |
| bronchiectasis | MSH5 |
| bronchiectasis | STX11 |
| bronchiectasis | CCN2 |
| bronchiectasis | CD68 |
| bronchiectasis | ACKR3 |
| bronchiectasis | ORMDL3 |
| bronchiectasis | NPTN |
| bronchiectasis | SWAP70 |
| bronchiectasis | TRIM29 |
| bronchiectasis | BBS5 |
| bronchiectasis | BBS9 |
| bronchiectasis | IL33 |
| bronchiectasis | ZBTB24 |
| bronchiectasis | CCL22 |
| bronchiectasis | IER3IP1 |
| bronchiectasis | VPREB1 |
| bronchiectasis | TSLP |
| bronchiectasis | GRK3 |
| bronchiectasis | TNFSF8 |
| bronchiectasis | ELMOD2 |
| bronchiectasis | ADAM33 |
| bronchiectasis | CCL18 |
| bronchiectasis | IRF2BP2 |
| bronchiectasis | NPAT |
| bronchiectasis | CEP83 |
| bronchiectasis | DPCD |
| bronchiectasis | SCLT1 |
| bronchiectasis | DOP1A |
| bronchiectasis | FAS-AS1 |
| bronchiectasis | ENSG00000251569 |
| bronchiectasis | MIR6774 |
| bronchiectasis | LOC105372146 |
| bronchiectasis | LOC105378353 |
| bronchiectasis | IGHE |
| bronchiectasis | MST1R |
| bronchiectasis | MST1 |
| bronchiectasis | SERPINB1 |
| bronchiectasis | IGES |
| bronchiectasis | CD14 |
| bronchiectasis | HMOX1 |
| bronchiectasis | CRP |
| bronchiectasis | CHGA |
| bronchiectasis | PKD1 |
| bronchiectasis | GAST |
| bronchiectasis | ITGAV |
| bronchiectasis | MMP2 |
| bronchiectasis | MMP14 |
| bronchiectasis | FUT2 |
| bronchiectasis | ITCH |
| bronchiectasis | PEPD |
| bronchiectasis | MINPP1 |
| bronchiectasis | HABP2 |
| bronchiectasis | FOXE1 |
| bronchiectasis | AFF4 |
| bronchiectasis | CSPP1 |
| bronchiectasis | KIAA0586 |
| bronchiectasis | LACTB |
| bronchiectasis | SAA1 |
| bronchiectasis | S100A12 |
| bronchiectasis | AQP3 |
| bronchiectasis | ITGAM |
| bronchiectasis | CEACAM3 |
| bronchiectasis | TMPRSS11D |
| bronchiectasis | PTCH1 |
| bronchiectasis | C3 |
| bronchiectasis | IL2RG |
| bronchiectasis | HSPG2 |
| bronchiectasis | CSTB |
| bronchiectasis | KRT19 |
| bronchiectasis | CST3 |
| bronchiectasis | CSTA |
| bronchiectasis | PADI4 |
| bronchiectasis | SDC1 |
| bronchiectasis | SBDS |
| bronchiectasis | G6PC3 |
| bronchiectasis | WFDC2 |
| bronchiectasis | MIR140 |
| bronchiectasis | CTNNB1 |
| bronchiectasis | NME1 |
| bronchiectasis | IL16 |
| bronchiectasis | PRSS2 |
| bronchiectasis | DNAH6 |
| bronchiectasis | TLR1 |
| bronchiectasis | TLR5 |
| bronchiectasis | DIAPH1 |
| bronchiectasis | TIMP3 |
| bronchiectasis | PRX |
| bronchiectasis | SLC26A9 |
| bronchiectasis | JAK3 |
| bronchiectasis | DPP4 |
| bronchiectasis | CAT |
| bronchiectasis | VDR |
| bronchiectasis | XIAP |
| bronchiectasis | WAS |
| bronchiectasis | CYP27B1 |
| bronchiectasis | PON1 |
| bronchiectasis | IKBKG |
| bronchiectasis | ADORA2A |
| bronchiectasis | COL4A3 |
| bronchiectasis | IL6ST |
| bronchiectasis | SELE |
| bronchiectasis | CSF1 |
| bronchiectasis | CEBPB |
| bronchiectasis | LBP |
| bronchiectasis | GC |
| bronchiectasis | CALCA |
| bronchiectasis | CORO1A |
| bronchiectasis | RAG2 |
| bronchiectasis | IL17F |
| bronchiectasis | GRP |
| bronchiectasis | RPGR |
| bronchiectasis | HPS1 |
| bronchiectasis | RBP3 |
| bronchiectasis | MSMB |
| bronchiectasis | STK16 |
| bronchiectasis | IGSF3 |
| bronchiectasis | DNAH9 |
| bronchiectasis | IL17D |
| bronchiectasis | MARCKS |
| bronchiectasis | DNALI1 |
| bronchiectasis | SPAG16 |
| bronchiectasis | FOXJ1 |
| bronchiectasis | SH2D4B |
| bronchiectasis | SERPINA2 |
