# Supplementary material for: Network Pharmacology Analysis of the Therapeutic Mechanisms Underlying Beimu-Gualou Formula Activity against Bronchiectasis with In Silico Molecular Docking Validation
Source: Evid Based Complement Alternat Med. 2021 Jan 5;2021:3656272. doi: 10.1155/2021/3656272 (PMC7803403; doi:10.1155/2021/3656272)
Supplement: Supplementary Materials — Supplementary Table 1: the chemical compounds of 6 herbs in BMGLF. Supplementary Table 2: the targets of BMGLF and bronchiectasis. Supplementary Table 3: the data of GO enrichment analysis. Supplementary Table 4: the data of KEGG pathway enrichment analysis. [file 3656272.f1.zip › 3656272.f1/Supplementary Table 3.docx]

| **GO Functional Enrichment** | | | | | | | | | | | | |
| --- | --- | --- | --- | --- | --- | --- | --- | --- | --- | --- | --- | --- |
| **Category** | **Term** | **Count** | **%** | **PValue** | **Genes** | **List Total** | **Pop Hits** | **Pop Total** | **Fold Enrichment** | **Bonferroni** | **Benjamini** | **FDR** |
| GOTERM_BP_DIRECT | GO:0042493~response to drug | 15 | 29.41176471 | 1.57E-13 | IL4, ICAM1, IL6, ADORA2A, SOD1, PTEN, IL10, ADA, STAT3, TGFB1, CDKN1A, CD86, IFNG, ABCC1, CAT | 51 | 304 | 16792 | 16.24613003 | 1.89E-10 | 1.89E-10 | 2.55E-10 |
| GOTERM_BP_DIRECT | GO:0071222~cellular response to lipopolysaccharide | 11 | 21.56862745 | 1.04E-12 | CSF2, ICAM1, IL6, CD86, CD80, IFNG, CXCL8, NFKB1, CD40, NOS2, IL10 | 51 | 113 | 16792 | 32.05136214 | 1.25E-09 | 6.25E-10 | 1.68E-09 |
| GOTERM_BP_DIRECT | GO:0006955~immune response | 15 | 29.41176471 | 1.29E-11 | IL4, CSF2, IL6, TLR2, CXCL8, IL13, FASLG, CD40, IL10, CD86, CD40LG, IKBKG, IFNG, MS4A2, IL2 | 51 | 421 | 16792 | 11.73117228 | 1.55E-08 | 5.16E-09 | 2.08E-08 |
| GOTERM_BP_DIRECT | GO:0007568~aging | 11 | 21.56862745 | 4.67E-11 | IL6, CD86, MPO, CAT, SOD1, PTEN, TGFB1, IL10, STAT3, ADA, TIMP1 | 51 | 165 | 16792 | 21.9503268 | 5.61E-08 | 1.40E-08 | 7.55E-08 |
| GOTERM_BP_DIRECT | GO:0051092~positive regulation of NF-kappaB transcription factor activity | 10 | 19.60784314 | 1.78E-10 | ICAM1, IL6, CD40LG, IKBKG, TLR2, NFKB1, CAT, CD40, TGFB1, BTK | 51 | 133 | 16792 | 24.75600767 | 2.13E-07 | 4.27E-08 | 2.87E-07 |
| GOTERM_BP_DIRECT | GO:0043066~negative regulation of apoptotic process | 14 | 27.45098039 | 5.10E-10 | IL4, IL6, MMP9, NFKB1, PTEN, IL10, STAT3, TIMP1, CDKN1A, CD40LG, ALB, MPO, CAT, IL2 | 51 | 455 | 16792 | 10.13092006 | 6.12E-07 | 1.02E-07 | 8.24E-07 |
| GOTERM_BP_DIRECT | GO:0045471~response to ethanol | 9 | 17.64705882 | 7.70E-10 | IL4, ICAM1, IL13, CAT, SOD1, PTEN, ADIPOQ, STAT3, IL2 | 51 | 105 | 16792 | 28.22184874 | 9.25E-07 | 1.32E-07 | 1.25E-06 |
| GOTERM_BP_DIRECT | GO:0006954~inflammatory response | 13 | 25.49019608 | 8.24E-10 | IL6, ADORA2A, CRP, TLR2, IL13, CXCL8, NFKB1, CD40, IL10, TGFB1, CD40LG, IKBKG, MS4A2 | 51 | 379 | 16792 | 11.29370376 | 9.89E-07 | 1.24E-07 | 1.33E-06 |
| GOTERM_BP_DIRECT | GO:0001666~response to hypoxia | 10 | 19.60784314 | 1.76E-09 | MUC1, HMOX1, TLR2, CAT, NOS2, ADIPOQ, MMP2, TGFB1, DPP4, ADA | 51 | 172 | 16792 | 19.14272686 | 2.11E-06 | 2.35E-07 | 2.84E-06 |
| GOTERM_BP_DIRECT | GO:0008284~positive regulation of cell proliferation | 12 | 23.52941176 | 9.40E-08 | CSF2, MAPK1, IL6, CD86, IFNG, FASLG, PTEN, TGFB1, DPP4, STAT3, TIMP1, IL2 | 51 | 466 | 16792 | 8.478667003 | 1.13E-04 | 1.13E-05 | 1.52E-04 |
| GOTERM_BP_DIRECT | GO:0030890~positive regulation of B cell proliferation | 6 | 11.76470588 | 1.02E-07 | IL4, CDKN1A, IL13, CD40, ADA, IL2 | 51 | 39 | 16792 | 50.6546003 | 1.22E-04 | 1.11E-05 | 1.64E-04 |
| GOTERM_BP_DIRECT | GO:0042832~defense response to protozoan | 5 | 9.803921569 | 2.61E-07 | IL4, IL6, IFNG, CD40, IL10 | 51 | 19 | 16792 | 86.64602683 | 3.13E-04 | 2.61E-05 | 4.22E-04 |
| GOTERM_BP_DIRECT | GO:0048304~positive regulation of isotype switching to IgG isotypes | 4 | 7.843137255 | 1.38E-06 | IL4, IFNG, CD40, IL2 | 51 | 8 | 16792 | 164.627451 | 0.001651912 | 1.27E-04 | 0.002225686 |
| GOTERM_BP_DIRECT | GO:0022617~extracellular matrix disassembly | 6 | 11.76470588 | 3.00E-06 | MMP9, MMP8, PRSS1, MMP2, MMP1, TIMP1 | 51 | 76 | 16792 | 25.99380805 | 0.003601024 | 2.58E-04 | 0.004856478 |
| GOTERM_BP_DIRECT | GO:0001774~microglial cell activation | 4 | 7.843137255 | 5.36E-06 | IL4, TRPV1, TLR2, IL13 | 51 | 12 | 16792 | 109.751634 | 0.006419993 | 4.29E-04 | 0.008670346 |
| GOTERM_BP_DIRECT | GO:0007267~cell-cell signaling | 8 | 15.68627451 | 9.57E-06 | CD86, ADRB2, CD80, ADORA2A, FASLG, IL13, IL10, IL2 | 51 | 254 | 16792 | 10.37023313 | 0.011423621 | 7.18E-04 | 0.015466271 |
| GOTERM_BP_DIRECT | GO:0008285~negative regulation of cell proliferation | 9 | 17.64705882 | 2.01E-05 | CDKN1A, IL6, ADORA2A, TLR2, CXCL8, PTEN, TGFB1, IL10, STAT3 | 51 | 396 | 16792 | 7.483065954 | 0.023865594 | 0.001419873 | 0.032513158 |
| GOTERM_BP_DIRECT | GO:0043011~myeloid dendritic cell differentiation | 4 | 7.843137255 | 2.33E-05 | IL4, CSF2, CD86, TGFB1 | 51 | 19 | 16792 | 69.31682147 | 0.027567588 | 0.001551834 | 0.037626695 |
| GOTERM_BP_DIRECT | GO:0032735~positive regulation of interleukin-12 production | 4 | 7.843137255 | 5.46E-05 | CD40LG, IFNG, TLR2, CD40 | 51 | 25 | 16792 | 52.68078431 | 0.063423156 | 0.003442676 | 0.08817182 |
| GOTERM_BP_DIRECT | GO:0071260~cellular response to mechanical stimulus | 5 | 9.803921569 | 5.83E-05 | IL13, CHEK1, NFKB1, CD40, TGFB1 | 51 | 71 | 16792 | 23.18696493 | 0.06767373 | 0.003497492 | 0.094289941 |
| GOTERM_BP_DIRECT | GO:2000352~negative regulation of endothelial cell apoptotic process | 4 | 7.843137255 | 7.72E-05 | IL4, ICAM1, IL13, IL10 | 51 | 28 | 16792 | 47.03641457 | 0.088573422 | 0.004406654 | 0.124778206 |
| GOTERM_BP_DIRECT | GO:0042113~B cell activation | 4 | 7.843137255 | 8.59E-05 | IL4, CD86, CD40, BTK | 51 | 29 | 16792 | 45.41446924 | 0.098079828 | 0.004681248 | 0.138874971 |
| GOTERM_BP_DIRECT | GO:0097421~liver regeneration | 4 | 7.843137255 | 8.59E-05 | HMOX1, PCNA, TGFB1, IL10 | 51 | 29 | 16792 | 45.41446924 | 0.098079828 | 0.004681248 | 0.138874971 |
| GOTERM_BP_DIRECT | GO:0050728~negative regulation of inflammatory response | 5 | 9.803921569 | 8.87E-05 | ADORA2A, NFKB1, ADIPOQ, ADA, IL2 | 51 | 79 | 16792 | 20.83891785 | 0.101006287 | 0.004618832 | 0.143244049 |
| GOTERM_BP_DIRECT | GO:0050731~positive regulation of peptidyl-tyrosine phosphorylation | 5 | 9.803921569 | 1.03E-04 | ICAM1, IL6, CD80, ADIPOQ, TGFB1 | 51 | 82 | 16792 | 20.07651841 | 0.115862446 | 0.005117802 | 0.165642339 |
| GOTERM_BP_DIRECT | GO:0045944~positive regulation of transcription from RNA polymerase II promoter | 12 | 23.52941176 | 1.16E-04 | IL4, IL6, ADRB2, IFNG, IKBKG, TLR2, NFKB1, CD40, TGFB1, IL10, STAT3, IL2 | 51 | 981 | 16792 | 4.027582899 | 0.13054425 | 0.005579888 | 0.188145559 |
| GOTERM_BP_DIRECT | GO:0010628~positive regulation of gene expression | 7 | 13.7254902 | 1.22E-04 | CSF2, IL6, CRP, IFNG, TLR2, TGFB1, STAT3 | 51 | 262 | 16792 | 8.796886694 | 0.136056308 | 0.005609121 | 0.196690972 |
| GOTERM_BP_DIRECT | GO:0045893~positive regulation of transcription, DNA-templated | 9 | 17.64705882 | 1.28E-04 | IL4, MAPK1, IL6, CD86, CD80, NFKB1, TGFB1, IL10, STAT3 | 51 | 515 | 16792 | 5.75396916 | 0.142525326 | 0.005678764 | 0.206788842 |
| GOTERM_BP_DIRECT | GO:0032355~response to estradiol | 5 | 9.803921569 | 1.54E-04 | PCNA, CAT, PTEN, TGFB1, STAT3 | 51 | 91 | 16792 | 18.09092868 | 0.168486322 | 0.006567891 | 0.248083409 |
| GOTERM_BP_DIRECT | GO:1901215~negative regulation of neuron death | 4 | 7.843137255 | 2.27E-04 | IL6, IKBKG, IL13, STAT3 | 51 | 40 | 16792 | 32.9254902 | 0.238738746 | 0.009362062 | 0.366552452 |
| GOTERM_BP_DIRECT | GO:0045348~positive regulation of MHC class II biosynthetic process | 3 | 5.882352941 | 2.41E-04 | IL4, IFNG, IL10 | 51 | 8 | 16792 | 123.4705882 | 0.250924942 | 0.009584311 | 0.38819532 |
| GOTERM_BP_DIRECT | GO:0045429~positive regulation of nitric oxide biosynthetic process | 4 | 7.843137255 | 2.82E-04 | ICAM1, IL6, TRPV1, IFNG | 51 | 43 | 16792 | 30.62836297 | 0.287232617 | 0.010863151 | 0.45480014 |
| GOTERM_BP_DIRECT | GO:0002237~response to molecule of bacterial origin | 3 | 5.882352941 | 3.09E-04 | TLR2, CXCL8, IL10 | 51 | 9 | 16792 | 109.751634 | 0.309796662 | 0.011519667 | 0.497900904 |
| GOTERM_BP_DIRECT | GO:0032930~positive regulation of superoxide anion generation | 3 | 5.882352941 | 3.09E-04 | CRP, SOD1, TGFB1 | 51 | 9 | 16792 | 109.751634 | 0.309796662 | 0.011519667 | 0.497900904 |
| GOTERM_BP_DIRECT | GO:0042110~T cell activation | 4 | 7.843137255 | 3.67E-04 | CD86, CD80, DPP4, ADA | 51 | 47 | 16792 | 28.02169378 | 0.356735933 | 0.013280718 | 0.592201113 |
| GOTERM_BP_DIRECT | GO:0045080~positive regulation of chemokine biosynthetic process | 3 | 5.882352941 | 3.85E-04 | IL4, HMOX1, IFNG | 51 | 10 | 16792 | 98.77647059 | 0.370353588 | 0.01351365 | 0.620831804 |
| GOTERM_BP_DIRECT | GO:0050765~negative regulation of phagocytosis | 3 | 5.882352941 | 3.85E-04 | PTEN, ADIPOQ, TGFB1 | 51 | 10 | 16792 | 98.77647059 | 0.370353588 | 0.01351365 | 0.620831804 |
| GOTERM_BP_DIRECT | GO:0042542~response to hydrogen peroxide | 4 | 7.843137255 | 4.68E-04 | HMOX1, CAT, SOD1, ADA | 51 | 51 | 16792 | 25.82391388 | 0.429897983 | 0.015927226 | 0.753652432 |
| GOTERM_BP_DIRECT | GO:0001878~response to yeast | 3 | 5.882352941 | 6.64E-04 | IL6, CD86, MPO | 51 | 13 | 16792 | 75.98190045 | 0.54949125 | 0.021905886 | 1.067721796 |
| GOTERM_BP_DIRECT | GO:0010629~negative regulation of gene expression | 5 | 9.803921569 | 7.30E-04 | CDKN1A, IFNG, NFKB1, NOS2, TGFB1 | 51 | 137 | 16792 | 12.01660226 | 0.584020905 | 0.023427174 | 1.173871213 |
| GOTERM_BP_DIRECT | GO:0042102~positive regulation of T cell proliferation | 4 | 7.843137255 | 7.54E-04 | IL4, IL6, CD40LG, IFNG | 51 | 60 | 16792 | 21.9503268 | 0.595984229 | 0.023567871 | 1.212687226 |
| GOTERM_BP_DIRECT | GO:2000353~positive regulation of endothelial cell apoptotic process | 3 | 5.882352941 | 7.73E-04 | CD40LG, FASLG, CD40 | 51 | 14 | 16792 | 70.55462185 | 0.604875973 | 0.023527916 | 1.242279007 |
| GOTERM_BP_DIRECT | GO:0006691~leukotriene metabolic process | 3 | 5.882352941 | 8.90E-04 | TLR2, ABCC1, ALOX5 | 51 | 15 | 16792 | 65.85098039 | 0.656797891 | 0.026381654 | 1.429404136 |
| GOTERM_BP_DIRECT | GO:1904707~positive regulation of vascular smooth muscle cell proliferation | 3 | 5.882352941 | 8.90E-04 | MMP9, MMP2, IL10 | 51 | 15 | 16792 | 65.85098039 | 0.656797891 | 0.026381654 | 1.429404136 |
| GOTERM_BP_DIRECT | GO:0030574~collagen catabolic process | 4 | 7.843137255 | 9.11E-04 | MMP9, MMP8, MMP2, MMP1 | 51 | 64 | 16792 | 20.57843137 | 0.665243331 | 0.026338427 | 1.462461553 |
| GOTERM_BP_DIRECT | GO:0050852~T cell receptor signaling pathway | 5 | 9.803921569 | 9.74E-04 | MAPK1, IFNG, IKBKG, NFKB1, PTEN | 51 | 148 | 16792 | 11.12347642 | 0.689726471 | 0.027479681 | 1.563161295 |
| GOTERM_BP_DIRECT | GO:0044130~negative regulation of growth of symbiont in host | 3 | 5.882352941 | 0.001015275 | IFNG, MPO, IL10 | 51 | 16 | 16792 | 61.73529412 | 0.704759569 | 0.027972599 | 1.628954179 |
| GOTERM_BP_DIRECT | GO:0032868~response to insulin | 4 | 7.843137255 | 0.001040671 | IL6, TLR2, CAT, IL10 | 51 | 67 | 16792 | 19.65700907 | 0.713637631 | 0.028020331 | 1.669379766 |
| GOTERM_BP_DIRECT | GO:0042523~positive regulation of tyrosine phosphorylation of Stat5 protein | 3 | 5.882352941 | 0.001148458 | IL4, CSF2, IL2 | 51 | 17 | 16792 | 58.10380623 | 0.748444414 | 0.030203182 | 1.840782578 |
| GOTERM_BP_DIRECT | GO:0070542~response to fatty acid | 3 | 5.882352941 | 0.001148458 | PON1, TLR2, CAT | 51 | 17 | 16792 | 58.10380623 | 0.748444414 | 0.030203182 | 1.840782578 |
| GOTERM_BP_DIRECT | GO:0071347~cellular response to interleukin-1 | 4 | 7.843137255 | 0.001231285 | ICAM1, IL6, CXCL8, NFKB1 | 51 | 71 | 16792 | 18.54957194 | 0.772290596 | 0.031655208 | 1.972303482 |
| GOTERM_BP_DIRECT | GO:0043123~positive regulation of I-kappaB kinase/NF-kappaB signaling | 5 | 9.803921569 | 0.001330272 | HMOX1, IKBKG, FASLG, CD40, ADIPOQ | 51 | 161 | 16792 | 10.22530751 | 0.797844935 | 0.033443307 | 2.129267182 |
| GOTERM_BP_DIRECT | GO:0006915~apoptotic process | 8 | 15.68627451 | 0.001365916 | MAPK1, ADORA2A, IFNG, IKBKG, TLR2, FASLG, NFKB1, PTEN | 51 | 567 | 16792 | 4.645571809 | 0.806327476 | 0.03362152 | 2.18573056 |
| GOTERM_BP_DIRECT | GO:0032496~response to lipopolysaccharide | 5 | 9.803921569 | 0.001423885 | TLR2, MPO, FASLG, IL13, CD40 | 51 | 164 | 16792 | 10.03825921 | 0.819369919 | 0.034321749 | 2.277492639 |
| GOTERM_BP_DIRECT | GO:0031295~T cell costimulation | 4 | 7.843137255 | 0.001615125 | CD86, CD80, CD40LG, DPP4 | 51 | 78 | 16792 | 16.88486677 | 0.856487558 | 0.038082577 | 2.579646862 |
| GOTERM_BP_DIRECT | GO:0046718~viral entry into host cell | 4 | 7.843137255 | 0.001737027 | ICAM1, CD86, CD80, DPP4 | 51 | 80 | 16792 | 16.4627451 | 0.876063221 | 0.040114101 | 2.771790698 |
| GOTERM_BP_DIRECT | GO:0038095~Fc-epsilon receptor signaling pathway | 5 | 9.803921569 | 0.001922245 | MAPK1, IKBKG, MS4A2, NFKB1, BTK | 51 | 178 | 16792 | 9.248733201 | 0.90082194 | 0.043466246 | 3.063054014 |
| GOTERM_BP_DIRECT | GO:0043065~positive regulation of apoptotic process | 6 | 11.76470588 | 0.001932363 | IL6, TRPV1, HMOX1, FASLG, SOD1, TGFB1 | 51 | 300 | 16792 | 6.585098039 | 0.902022115 | 0.042883771 | 3.078941186 |
| GOTERM_BP_DIRECT | GO:0009636~response to toxic substance | 4 | 7.843137255 | 0.002066532 | MAPK1, CDKN1A, PON1, TLR2 | 51 | 85 | 16792 | 15.49434833 | 0.916630471 | 0.044966393 | 3.28938257 |
| GOTERM_BP_DIRECT | GO:0032733~positive regulation of interleukin-10 production | 3 | 5.882352941 | 0.002112238 | IL4, CD40LG, TLR2 | 51 | 23 | 16792 | 42.94629156 | 0.921092642 | 0.045122652 | 3.360974697 |
| GOTERM_BP_DIRECT | GO:0006928~movement of cell or subcellular component | 4 | 7.843137255 | 0.002136765 | IFNG, CXCL8, IL13, STAT3 | 51 | 86 | 16792 | 15.31418149 | 0.923387945 | 0.044838664 | 3.399372465 |
| GOTERM_BP_DIRECT | GO:0042104~positive regulation of activated T cell proliferation | 3 | 5.882352941 | 0.002908238 | IL4, CD86, IL2 | 51 | 27 | 16792 | 36.583878 | 0.969738625 | 0.059521387 | 4.599841407 |
| GOTERM_BP_DIRECT | GO:0035987~endodermal cell differentiation | 3 | 5.882352941 | 0.002908238 | MMP9, MMP8, MMP2 | 51 | 27 | 16792 | 36.583878 | 0.969738625 | 0.059521387 | 4.599841407 |
| GOTERM_BP_DIRECT | GO:0048662~negative regulation of smooth muscle cell proliferation | 3 | 5.882352941 | 0.003351199 | HMOX1, IFNG, ADIPOQ | 51 | 29 | 16792 | 34.06085193 | 0.982253176 | 0.067148692 | 5.282780581 |
| GOTERM_BP_DIRECT | GO:0006974~cellular response to DNA damage stimulus | 5 | 9.803921569 | 0.003374325 | MAPK1, CDKN1A, VCP, IKBKG, CHEK1 | 51 | 208 | 16792 | 7.914781297 | 0.98274093 | 0.066490077 | 5.318309999 |
| GOTERM_BP_DIRECT | GO:0002576~platelet degranulation | 4 | 7.843137255 | 0.003564006 | ALB, SOD1, TGFB1, TIMP1 | 51 | 103 | 16792 | 12.78659813 | 0.986267868 | 0.068972949 | 5.609244846 |
| GOTERM_BP_DIRECT | GO:0051091~positive regulation of sequence-specific DNA binding transcription factor activity | 4 | 7.843137255 | 0.003762113 | IL4, IL6, PTEN, IL10 | 51 | 105 | 16792 | 12.54304388 | 0.989184963 | 0.071523425 | 5.912208491 |
| GOTERM_BP_DIRECT | GO:0001975~response to amphetamine | 3 | 5.882352941 | 0.003823658 | ICAM1, ADORA2A, SOD1 | 51 | 31 | 16792 | 31.86337761 | 0.989958361 | 0.071523231 | 6.006143352 |
| GOTERM_BP_DIRECT | GO:0042100~B cell proliferation | 3 | 5.882352941 | 0.004070839 | CD40LG, CD40, IL10 | 51 | 32 | 16792 | 30.86764706 | 0.992546386 | 0.074816143 | 6.382524433 |
| GOTERM_BP_DIRECT | GO:0031663~lipopolysaccharide-mediated signaling pathway | 3 | 5.882352941 | 0.004070839 | MAPK1, TLR2, TGFB1 | 51 | 32 | 16792 | 30.86764706 | 0.992546386 | 0.074816143 | 6.382524433 |
| GOTERM_BP_DIRECT | GO:0071356~cellular response to tumor necrosis factor | 4 | 7.843137255 | 0.004286445 | ICAM1, IL6, TRPV1, CXCL8 | 51 | 110 | 16792 | 11.97290553 | 0.994253045 | 0.077447233 | 6.709670295 |
| GOTERM_BP_DIRECT | GO:1902042~negative regulation of extrinsic apoptotic signaling pathway via death domain receptors | 3 | 5.882352941 | 0.004325263 | ICAM1, HMOX1, FASLG | 51 | 33 | 16792 | 29.93226381 | 0.994515931 | 0.076967552 | 6.768457125 |
| GOTERM_BP_DIRECT | GO:0043491~protein kinase B signaling | 3 | 5.882352941 | 0.004325263 | CD40, PTEN, TGFB1 | 51 | 33 | 16792 | 29.93226381 | 0.994515931 | 0.076967552 | 6.768457125 |
| GOTERM_BP_DIRECT | GO:0030168~platelet activation | 4 | 7.843137255 | 0.00485311 | MAPK1, IL6, CD40LG, CD40 | 51 | 115 | 16792 | 11.45234442 | 0.997099217 | 0.084721497 | 7.564385072 |
| GOTERM_BP_DIRECT | GO:0050714~positive regulation of protein secretion | 3 | 5.882352941 | 0.005131558 | ADORA2A, IL13, TGFB1 | 51 | 36 | 16792 | 27.4379085 | 0.997927229 | 0.088097245 | 7.981678284 |
| GOTERM_BP_DIRECT | GO:0034605~cellular response to heat | 3 | 5.882352941 | 0.00541452 | CDKN1A, TRPV1, HMOX1 | 51 | 37 | 16792 | 26.6963434 | 0.998527079 | 0.09143588 | 8.403924575 |
| GOTERM_BP_DIRECT | GO:0006953~acute-phase response | 3 | 5.882352941 | 0.006001482 | IL6, CRP, STAT3 | 51 | 39 | 16792 | 25.32730015 | 0.999275113 | 0.099473148 | 9.274021562 |
| GOTERM_BP_DIRECT | GO:0001934~positive regulation of protein phosphorylation | 4 | 7.843137255 | 0.006391404 | MMP9, CD40, ADIPOQ, TGFB1 | 51 | 127 | 16792 | 10.37023313 | 0.999547495 | 0.104174966 | 9.847738323 |
| GOTERM_BP_DIRECT | GO:0007566~embryo implantation | 3 | 5.882352941 | 0.006933883 | MMP9, SOD1, MMP2 | 51 | 42 | 16792 | 23.51820728 | 0.999765158 | 0.111036032 | 10.64026342 |
| GOTERM_BP_DIRECT | GO:0045740~positive regulation of DNA replication | 3 | 5.882352941 | 0.006933883 | CSF2, IL6, PCNA | 51 | 42 | 16792 | 23.51820728 | 0.999765158 | 0.111036032 | 10.64026342 |
| GOTERM_BP_DIRECT | GO:0045599~negative regulation of fat cell differentiation | 3 | 5.882352941 | 0.006933883 | IL6, ADIPOQ, TGFB1 | 51 | 42 | 16792 | 23.51820728 | 0.999765158 | 0.111036032 | 10.64026342 |
| GOTERM_BP_DIRECT | GO:0097191~extrinsic apoptotic signaling pathway | 3 | 5.882352941 | 0.006933883 | IFNG, FASLG, TGFB1 | 51 | 42 | 16792 | 23.51820728 | 0.999765158 | 0.111036032 | 10.64026342 |
| GOTERM_BP_DIRECT | GO:0014823~response to activity | 3 | 5.882352941 | 0.00725837 | CAT, ADIPOQ, IL10 | 51 | 43 | 16792 | 22.97127223 | 0.999841395 | 0.114422394 | 11.11118812 |
| GOTERM_BP_DIRECT | GO:0007050~cell cycle arrest | 4 | 7.843137255 | 0.008516473 | CDKN1A, IFNG, CXCL8, TGFB1 | 51 | 141 | 16792 | 9.340564595 | 0.999965415 | 0.131261846 | 12.91513372 |
| GOTERM_BP_DIRECT | GO:0014854~response to inactivity | 2 | 3.921568627 | 0.008906782 | CAT, IL10 | 51 | 3 | 16792 | 219.503268 | 0.999978446 | 0.135152658 | 13.46776567 |
| GOTERM_BP_DIRECT | GO:0001781~neutrophil apoptotic process | 2 | 3.921568627 | 0.008906782 | IL6, IFNG | 51 | 3 | 16792 | 219.503268 | 0.999978446 | 0.135152658 | 13.46776567 |
| GOTERM_BP_DIRECT | GO:0034465~response to carbon monoxide | 2 | 3.921568627 | 0.008906782 | SOD1, IL10 | 51 | 3 | 16792 | 219.503268 | 0.999978446 | 0.135152658 | 13.46776567 |
| GOTERM_BP_DIRECT | GO:1903660~negative regulation of complement-dependent cytotoxicity | 2 | 3.921568627 | 0.008906782 | IL4, IL13 | 51 | 3 | 16792 | 219.503268 | 0.999978446 | 0.135152658 | 13.46776567 |
| GOTERM_BP_DIRECT | GO:0050954~sensory perception of mechanical stimulus | 2 | 3.921568627 | 0.008906782 | TRPV1, IFNG | 51 | 3 | 16792 | 219.503268 | 0.999978446 | 0.135152658 | 13.46776567 |
| GOTERM_BP_DIRECT | GO:0051712~positive regulation of killing of cells of other organism | 2 | 3.921568627 | 0.008906782 | IFNG, NOS2 | 51 | 3 | 16792 | 219.503268 | 0.999978446 | 0.135152658 | 13.46776567 |
| GOTERM_BP_DIRECT | GO:0002902~regulation of B cell apoptotic process | 2 | 3.921568627 | 0.008906782 | PTEN, BTK | 51 | 3 | 16792 | 219.503268 | 0.999978446 | 0.135152658 | 13.46776567 |
| GOTERM_BP_DIRECT | GO:0033632~regulation of cell-cell adhesion mediated by integrin | 2 | 3.921568627 | 0.008906782 | DPP4, ADA | 51 | 3 | 16792 | 219.503268 | 0.999978446 | 0.135152658 | 13.46776567 |
| GOTERM_BP_DIRECT | GO:0042742~defense response to bacterium | 4 | 7.843137255 | 0.009191021 | IFNG, MPO, NOS2, IL10 | 51 | 145 | 16792 | 9.082893847 | 0.999984727 | 0.137447634 | 13.86814223 |
| GOTERM_BP_DIRECT | GO:0002250~adaptive immune response | 4 | 7.843137255 | 0.009716944 | CD86, IFNG, BTK, IL2 | 51 | 148 | 16792 | 8.898781134 | 0.999991928 | 0.142988718 | 14.60437452 |
| GOTERM_BP_DIRECT | GO:0001821~histamine secretion | 2 | 3.921568627 | 0.011858397 | CSF2, ADA | 51 | 4 | 16792 | 164.627451 | 0.9999994 | 0.169780871 | 17.54163385 |
| GOTERM_BP_DIRECT | GO:0071677~positive regulation of mononuclear cell migration | 2 | 3.921568627 | 0.011858397 | IL4, TGFB1 | 51 | 4 | 16792 | 164.627451 | 0.9999994 | 0.169780871 | 17.54163385 |
| GOTERM_BP_DIRECT | GO:0010716~negative regulation of extracellular matrix disassembly | 2 | 3.921568627 | 0.011858397 | TGFB1, DPP4 | 51 | 4 | 16792 | 164.627451 | 0.9999994 | 0.169780871 | 17.54163385 |
| GOTERM_BP_DIRECT | GO:0045191~regulation of isotype switching | 2 | 3.921568627 | 0.011858397 | IL4, IL10 | 51 | 4 | 16792 | 164.627451 | 0.9999994 | 0.169780871 | 17.54163385 |
| GOTERM_BP_DIRECT | GO:0010155~regulation of proton transport | 2 | 3.921568627 | 0.011858397 | IL4, IL13 | 51 | 4 | 16792 | 164.627451 | 0.9999994 | 0.169780871 | 17.54163385 |
| GOTERM_BP_DIRECT | GO:0051607~defense response to virus | 4 | 7.843137255 | 0.013026663 | IL6, CD86, IFNG, CD40 | 51 | 165 | 16792 | 7.981937017 | 0.999999855 | 0.182819344 | 19.10382178 |
| GOTERM_BP_DIRECT | GO:0048661~positive regulation of smooth muscle cell proliferation | 3 | 5.882352941 | 0.013777687 | IL6, HMOX1, IL13 | 51 | 60 | 16792 | 16.4627451 | 0.999999942 | 0.190154691 | 20.09337249 |
| GOTERM_BP_DIRECT | GO:0007249~I-kappaB kinase/NF-kappaB signaling | 3 | 5.882352941 | 0.013777687 | IKBKG, NFKB1, BTK | 51 | 60 | 16792 | 16.4627451 | 0.999999942 | 0.190154691 | 20.09337249 |
| GOTERM_BP_DIRECT | GO:0001933~negative regulation of protein phosphorylation | 3 | 5.882352941 | 0.01421789 | PTEN, TGFB1, IL2 | 51 | 61 | 16792 | 16.19286403 | 0.999999966 | 0.19344081 | 20.66809381 |
| GOTERM_BP_DIRECT | GO:0006977~DNA damage response, signal transduction by p53 class mediator resulting in cell cycle arrest | 3 | 5.882352941 | 0.014664146 | MUC1, CDKN1A, PCNA | 51 | 62 | 16792 | 15.9316888 | 0.99999998 | 0.196708487 | 21.24675755 |
| GOTERM_BP_DIRECT | GO:0051023~regulation of immunoglobulin secretion | 2 | 3.921568627 | 0.014801397 | CD40LG, CD40 | 51 | 5 | 16792 | 131.7019608 | 0.999999983 | 0.196201519 | 21.42393363 |
| GOTERM_BP_DIRECT | GO:0032747~positive regulation of interleukin-23 production | 2 | 3.921568627 | 0.014801397 | CSF2, IFNG | 51 | 5 | 16792 | 131.7019608 | 0.999999983 | 0.196201519 | 21.42393363 |
| GOTERM_BP_DIRECT | GO:0042092~type 2 immune response | 2 | 3.921568627 | 0.014801397 | IL4, IL10 | 51 | 5 | 16792 | 131.7019608 | 0.999999983 | 0.196201519 | 21.42393363 |
| GOTERM_BP_DIRECT | GO:0070374~positive regulation of ERK1 and ERK2 cascade | 4 | 7.843137255 | 0.015239694 | ICAM1, IL6, PTEN, TGFB1 | 51 | 175 | 16792 | 7.525826331 | 0.99999999 | 0.199256094 | 21.98722903 |
| GOTERM_BP_DIRECT | GO:0050776~regulation of immune response | 4 | 7.843137255 | 0.015942566 | IL4, ICAM1, CD40LG, CD40 | 51 | 178 | 16792 | 7.398986561 | 0.999999996 | 0.205289408 | 22.88265234 |
| GOTERM_BP_DIRECT | GO:0051384~response to glucocorticoid | 3 | 5.882352941 | 0.016038846 | IL6, ADIPOQ, IL10 | 51 | 65 | 16792 | 15.19638009 | 0.999999996 | 0.204239082 | 23.0045547 |
| GOTERM_BP_DIRECT | GO:0006508~proteolysis | 6 | 11.76470588 | 0.016165826 | MMP9, MMP8, PRSS1, MMP2, DPP4, MMP1 | 51 | 500 | 16792 | 3.951058824 | 0.999999997 | 0.203559084 | 23.16505024 |
| GOTERM_BP_DIRECT | GO:0030183~B cell differentiation | 3 | 5.882352941 | 0.016508931 | IL4, CD40LG, IL10 | 51 | 66 | 16792 | 14.96613191 | 0.999999998 | 0.205308504 | 23.59714661 |
| GOTERM_BP_DIRECT | GO:0009749~response to glucose | 3 | 5.882352941 | 0.017466648 | PTEN, ADIPOQ, TGFB1 | 51 | 68 | 16792 | 14.52595156 | 0.999999999 | 0.213754713 | 24.79123907 |
| GOTERM_BP_DIRECT | GO:0008203~cholesterol metabolic process | 3 | 5.882352941 | 0.017466648 | IL4, PON1, CAT | 51 | 68 | 16792 | 14.52595156 | 0.999999999 | 0.213754713 | 24.79123907 |
| GOTERM_BP_DIRECT | GO:0042127~regulation of cell proliferation | 4 | 7.843137255 | 0.017653 | CHEK1, CD40, NOS2, BTK | 51 | 185 | 16792 | 7.119024907 | 0.999999999 | 0.213642707 | 25.02154006 |
| GOTERM_BP_DIRECT | GO:2001171~positive regulation of ATP biosynthetic process | 2 | 3.921568627 | 0.017735806 | VCP, STAT3 | 51 | 6 | 16792 | 109.751634 | 1 | 0.212426385 | 25.12366264 |
| GOTERM_BP_DIRECT | GO:0051045~negative regulation of membrane protein ectodomain proteolysis | 2 | 3.921568627 | 0.017735806 | IL10, TIMP1 | 51 | 6 | 16792 | 109.751634 | 1 | 0.212426385 | 25.12366264 |
| GOTERM_BP_DIRECT | GO:0045591~positive regulation of regulatory T cell differentiation | 2 | 3.921568627 | 0.017735806 | TGFB1, IL2 | 51 | 6 | 16792 | 109.751634 | 1 | 0.212426385 | 25.12366264 |
| GOTERM_BP_DIRECT | GO:0002686~negative regulation of leukocyte migration | 2 | 3.921568627 | 0.017735806 | HMOX1, ADA | 51 | 6 | 16792 | 109.751634 | 1 | 0.212426385 | 25.12366264 |
| GOTERM_BP_DIRECT | GO:0097190~apoptotic signaling pathway | 3 | 5.882352941 | 0.018946536 | FASLG, CD40, BTK | 51 | 71 | 16792 | 13.91217896 | 1 | 0.223106343 | 26.60200251 |
| GOTERM_BP_DIRECT | GO:0007584~response to nutrient | 3 | 5.882352941 | 0.020477508 | IL4, PTEN, ADIPOQ | 51 | 74 | 16792 | 13.3481717 | 1 | 0.236693394 | 28.43217514 |
| GOTERM_BP_DIRECT | GO:0071312~cellular response to alkaloid | 2 | 3.921568627 | 0.020661649 | ICAM1, TRPV1 | 51 | 7 | 16792 | 94.07282913 | 1 | 0.236329738 | 28.6493964 |
| GOTERM_BP_DIRECT | GO:0031642~negative regulation of myelination | 2 | 3.921568627 | 0.020661649 | IFNG, PTEN | 51 | 7 | 16792 | 94.07282913 | 1 | 0.236329738 | 28.6493964 |
| GOTERM_BP_DIRECT | GO:0014066~regulation of phosphatidylinositol 3-kinase signaling | 3 | 5.882352941 | 0.02259656 | MAPK1, CD86, CD80 | 51 | 78 | 16792 | 12.66365008 | 1 | 0.253245649 | 30.89480883 |
| GOTERM_BP_DIRECT | GO:0010888~negative regulation of lipid storage | 2 | 3.921568627 | 0.023578952 | IL6, CRP | 51 | 8 | 16792 | 82.31372549 | 1 | 0.260409129 | 32.00930811 |
| GOTERM_BP_DIRECT | GO:0034383~low-density lipoprotein particle clearance | 2 | 3.921568627 | 0.023578952 | HMOX1, ADIPOQ | 51 | 8 | 16792 | 82.31372549 | 1 | 0.260409129 | 32.00930811 |
| GOTERM_BP_DIRECT | GO:1904706~negative regulation of vascular smooth muscle cell proliferation | 2 | 3.921568627 | 0.023578952 | HMOX1, IL10 | 51 | 8 | 16792 | 82.31372549 | 1 | 0.260409129 | 32.00930811 |
| GOTERM_BP_DIRECT | GO:0045630~positive regulation of T-helper 2 cell differentiation | 2 | 3.921568627 | 0.023578952 | IL6, CD86 | 51 | 8 | 16792 | 82.31372549 | 1 | 0.260409129 | 32.00930811 |
| GOTERM_BP_DIRECT | GO:0034115~negative regulation of heterotypic cell-cell adhesion | 2 | 3.921568627 | 0.023578952 | ADIPOQ, IL10 | 51 | 8 | 16792 | 82.31372549 | 1 | 0.260409129 | 32.00930811 |
| GOTERM_BP_DIRECT | GO:0007626~locomotory behavior | 3 | 5.882352941 | 0.025937087 | ADORA2A, SOD1, PTEN | 51 | 84 | 16792 | 11.75910364 | 1 | 0.280188608 | 34.61612285 |
| GOTERM_BP_DIRECT | GO:0070102~interleukin-6-mediated signaling pathway | 2 | 3.921568627 | 0.026487737 | IL6, STAT3 | 51 | 9 | 16792 | 73.16775599 | 1 | 0.282783893 | 35.21118749 |
| GOTERM_BP_DIRECT | GO:0002024~diet induced thermogenesis | 2 | 3.921568627 | 0.026487737 | ADRB2, TRPV1 | 51 | 9 | 16792 | 73.16775599 | 1 | 0.282783893 | 35.21118749 |
| GOTERM_BP_DIRECT | GO:0050830~defense response to Gram-positive bacterium | 3 | 5.882352941 | 0.026512281 | IL6, CRP, TLR2 | 51 | 85 | 16792 | 11.62076125 | 1 | 0.280569567 | 35.23759268 |
| GOTERM_BP_DIRECT | GO:0001525~angiogenesis | 4 | 7.843137255 | 0.028671722 | HMOX1, CXCL8, PTEN, MMP2 | 51 | 223 | 16792 | 5.905917524 | 1 | 0.297359169 | 37.52165761 |
| GOTERM_BP_DIRECT | GO:0007565~female pregnancy | 3 | 5.882352941 | 0.028864466 | MUC1, IL4, TGFB1 | 51 | 89 | 16792 | 11.09847984 | 1 | 0.296553708 | 37.72180771 |
| GOTERM_BP_DIRECT | GO:0046888~negative regulation of hormone secretion | 2 | 3.921568627 | 0.02938803 | IL6, ADIPOQ | 51 | 10 | 16792 | 65.85098039 | 1 | 0.298611587 | 38.26245878 |
| GOTERM_BP_DIRECT | GO:0042511~positive regulation of tyrosine phosphorylation of Stat1 protein | 2 | 3.921568627 | 0.032279854 | IFNG, CD40 | 51 | 11 | 16792 | 59.86452763 | 1 | 0.320466814 | 41.17019781 |
| GOTERM_BP_DIRECT | GO:0032700~negative regulation of interleukin-17 production | 2 | 3.921568627 | 0.032279854 | IFNG, TGFB1 | 51 | 11 | 16792 | 59.86452763 | 1 | 0.320466814 | 41.17019781 |
| GOTERM_BP_DIRECT | GO:0043306~positive regulation of mast cell degranulation | 2 | 3.921568627 | 0.032279854 | IL4, IL13 | 51 | 11 | 16792 | 59.86452763 | 1 | 0.320466814 | 41.17019781 |
| GOTERM_BP_DIRECT | GO:0033197~response to vitamin E | 2 | 3.921568627 | 0.032279854 | CAT, ADA | 51 | 11 | 16792 | 59.86452763 | 1 | 0.320466814 | 41.17019781 |
| GOTERM_BP_DIRECT | GO:0051024~positive regulation of immunoglobulin secretion | 2 | 3.921568627 | 0.032279854 | IL6, IL2 | 51 | 11 | 16792 | 59.86452763 | 1 | 0.320466814 | 41.17019781 |
| GOTERM_BP_DIRECT | GO:0071456~cellular response to hypoxia | 3 | 5.882352941 | 0.033173453 | ICAM1, HMOX1, PTEN | 51 | 96 | 16792 | 10.28921569 | 1 | 0.325221181 | 42.0424003 |
| GOTERM_BP_DIRECT | GO:0019430~removal of superoxide radicals | 2 | 3.921568627 | 0.035163235 | MPO, SOD1 | 51 | 12 | 16792 | 54.87581699 | 1 | 0.338588419 | 43.94114842 |
| GOTERM_BP_DIRECT | GO:0031000~response to caffeine | 2 | 3.921568627 | 0.035163235 | IL6, ADORA2A | 51 | 12 | 16792 | 54.87581699 | 1 | 0.338588419 | 43.94114842 |
| GOTERM_BP_DIRECT | GO:0031669~cellular response to nutrient levels | 2 | 3.921568627 | 0.035163235 | ICAM1, IL6 | 51 | 12 | 16792 | 54.87581699 | 1 | 0.338588419 | 43.94114842 |
| GOTERM_BP_DIRECT | GO:0097028~dendritic cell differentiation | 2 | 3.921568627 | 0.035163235 | IL4, CSF2 | 51 | 12 | 16792 | 54.87581699 | 1 | 0.338588419 | 43.94114842 |
| GOTERM_BP_DIRECT | GO:0045086~positive regulation of interleukin-2 biosynthetic process | 2 | 3.921568627 | 0.035163235 | CD86, CD80 | 51 | 12 | 16792 | 54.87581699 | 1 | 0.338588419 | 43.94114842 |
| GOTERM_BP_DIRECT | GO:0090399~replicative senescence | 2 | 3.921568627 | 0.035163235 | CDKN1A, CHEK1 | 51 | 12 | 16792 | 54.87581699 | 1 | 0.338588419 | 43.94114842 |
| GOTERM_BP_DIRECT | GO:0043032~positive regulation of macrophage activation | 2 | 3.921568627 | 0.035163235 | IL13, IL10 | 51 | 12 | 16792 | 54.87581699 | 1 | 0.338588419 | 43.94114842 |
| GOTERM_BP_DIRECT | GO:0042593~glucose homeostasis | 3 | 5.882352941 | 0.036396071 | IL6, ADIPOQ, STAT3 | 51 | 101 | 16792 | 9.779848573 | 1 | 0.345619694 | 45.08813409 |
| GOTERM_BP_DIRECT | GO:0032740~positive regulation of interleukin-17 production | 2 | 3.921568627 | 0.038038195 | TGFB1, IL2 | 51 | 13 | 16792 | 50.6546003 | 1 | 0.355571084 | 46.58173792 |
| GOTERM_BP_DIRECT | GO:0060134~prepulse inhibition | 2 | 3.921568627 | 0.038038195 | ADORA2A, PTEN | 51 | 13 | 16792 | 50.6546003 | 1 | 0.355571084 | 46.58173792 |
| GOTERM_BP_DIRECT | GO:0045019~negative regulation of nitric oxide biosynthetic process | 2 | 3.921568627 | 0.038038195 | IL4, IL10 | 51 | 13 | 16792 | 50.6546003 | 1 | 0.355571084 | 46.58173792 |
| GOTERM_BP_DIRECT | GO:0010745~negative regulation of macrophage derived foam cell differentiation | 2 | 3.921568627 | 0.038038195 | CRP, ADIPOQ | 51 | 13 | 16792 | 50.6546003 | 1 | 0.355571084 | 46.58173792 |
| GOTERM_BP_DIRECT | GO:0071318~cellular response to ATP | 2 | 3.921568627 | 0.038038195 | TRPV1, SOD1 | 51 | 13 | 16792 | 50.6546003 | 1 | 0.355571084 | 46.58173792 |
| GOTERM_BP_DIRECT | GO:0046685~response to arsenic-containing substance | 2 | 3.921568627 | 0.038038195 | CDKN1A, PTEN | 51 | 13 | 16792 | 50.6546003 | 1 | 0.355571084 | 46.58173792 |
| GOTERM_BP_DIRECT | GO:0048015~phosphatidylinositol-mediated signaling | 3 | 5.882352941 | 0.039734694 | CD86, CD80, PTEN | 51 | 106 | 16792 | 9.318534961 | 1 | 0.365613416 | 48.08471446 |
| GOTERM_BP_DIRECT | GO:0000187~activation of MAPK activity | 3 | 5.882352941 | 0.040415987 | MAPK1, IKBKG, SOD1 | 51 | 107 | 16792 | 9.231445849 | 1 | 0.367942824 | 48.67705068 |
| GOTERM_BP_DIRECT | GO:0035729~cellular response to hepatocyte growth factor stimulus | 2 | 3.921568627 | 0.04090476 | IL6, IL10 | 51 | 14 | 16792 | 47.03641457 | 1 | 0.3688301 | 49.098092 |
| GOTERM_BP_DIRECT | GO:0045721~negative regulation of gluconeogenesis | 2 | 3.921568627 | 0.04090476 | IL6, ADIPOQ | 51 | 14 | 16792 | 47.03641457 | 1 | 0.3688301 | 49.098092 |
| GOTERM_BP_DIRECT | GO:0010875~positive regulation of cholesterol efflux | 2 | 3.921568627 | 0.04090476 | PON1, ADIPOQ | 51 | 14 | 16792 | 47.03641457 | 1 | 0.3688301 | 49.098092 |
| GOTERM_BP_DIRECT | GO:0000165~MAPK cascade | 4 | 7.843137255 | 0.043013722 | CSF2, MAPK1, TGFB1, IL2 | 51 | 262 | 16792 | 5.026792396 | 1 | 0.381235946 | 50.87792463 |
| GOTERM_BP_DIRECT | GO:0060397~JAK-STAT cascade involved in growth hormone signaling pathway | 2 | 3.921568627 | 0.043762953 | MAPK1, STAT3 | 51 | 15 | 16792 | 43.90065359 | 1 | 0.383798262 | 51.49604883 |
| GOTERM_BP_DIRECT | GO:0046688~response to copper ion | 2 | 3.921568627 | 0.043762953 | ICAM1, SOD1 | 51 | 15 | 16792 | 43.90065359 | 1 | 0.383798262 | 51.49604883 |
| GOTERM_BP_DIRECT | GO:0032270~positive regulation of cellular protein metabolic process | 2 | 3.921568627 | 0.043762953 | ADIPOQ, TGFB1 | 51 | 15 | 16792 | 43.90065359 | 1 | 0.383798262 | 51.49604883 |
| GOTERM_BP_DIRECT | GO:0010744~positive regulation of macrophage derived foam cell differentiation | 2 | 3.921568627 | 0.046612798 | CSF2, NFKB1 | 51 | 16 | 16792 | 41.15686275 | 1 | 0.400622478 | 53.78117255 |
| GOTERM_BP_DIRECT | GO:0048566~embryonic digestive tract development | 2 | 3.921568627 | 0.046612798 | CXCL8, ADA | 51 | 16 | 16792 | 41.15686275 | 1 | 0.400622478 | 53.78117255 |
| GOTERM_BP_DIRECT | GO:0006978~DNA damage response, signal transduction by p53 class mediator resulting in transcription of p21 class mediator | 2 | 3.921568627 | 0.046612798 | MUC1, CDKN1A | 51 | 16 | 16792 | 41.15686275 | 1 | 0.400622478 | 53.78117255 |
| GOTERM_BP_DIRECT | GO:0033209~tumor necrosis factor-mediated signaling pathway | 3 | 5.882352941 | 0.048197 | CD40LG, FASLG, CD40 | 51 | 118 | 16792 | 8.370887338 | 1 | 0.408450058 | 55.00737309 |
| GOTERM_BP_DIRECT | GO:0044267~cellular protein metabolic process | 3 | 5.882352941 | 0.048197 | ADORA2A, MMP2, MMP1 | 51 | 118 | 16792 | 8.370887338 | 1 | 0.408450058 | 55.00737309 |
| GOTERM_BP_DIRECT | GO:0032722~positive regulation of chemokine production | 2 | 3.921568627 | 0.049454319 | IL6, TLR2 | 51 | 17 | 16792 | 38.73587082 | 1 | 0.413938023 | 55.95876615 |
| GOTERM_BP_DIRECT | GO:0071276~cellular response to cadmium ion | 2 | 3.921568627 | 0.049454319 | HMOX1, SOD1 | 51 | 17 | 16792 | 38.73587082 | 1 | 0.413938023 | 55.95876615 |
| GOTERM_BP_DIRECT | GO:0055093~response to hyperoxia | 2 | 3.921568627 | 0.049454319 | CDKN1A, CAT | 51 | 17 | 16792 | 38.73587082 | 1 | 0.413938023 | 55.95876615 |
