# Supplementary material for: Network Pharmacology Analysis of the Therapeutic Mechanisms Underlying Beimu-Gualou Formula Activity against Bronchiectasis with In Silico Molecular Docking Validation
Source: Evid Based Complement Alternat Med. 2021 Jan 5;2021:3656272. doi: 10.1155/2021/3656272 (PMC7803403; doi:10.1155/2021/3656272)
Supplement: Supplementary Materials — Supplementary Table 1: the chemical compounds of 6 herbs in BMGLF. Supplementary Table 2: the targets of BMGLF and bronchiectasis. Supplementary Table 3: the data of GO enrichment analysis. Supplementary Table 4: the data of KEGG pathway enrichment analysis. [file 3656272.f1.zip › 3656272.f1/Supplementary Table 4.docx]

| **KEGG Pathway Enrichment** | | | | | | | | | | | | |
| --- | --- | --- | --- | --- | --- | --- | --- | --- | --- | --- | --- | --- |
| **Category** | **Term** | **Count** | **%** | **PValue** | **Genes** | **List Total** | **Pop Hits** | **Pop Total** | **Fold Enrichment** | **Bonferroni** | **Benjamini** | **FDR** |
| KEGG_PATHWAY | hsa05142:Chagas disease (American trypanosomiasis) | 12 | 23.52941176 | 3.65E-11 | MAPK1, IL6, IFNG, IKBKG, TLR2, CXCL8, FASLG, NFKB1, NOS2, TGFB1, IL10, IL2 | 46 | 104 | 6879 | 17.25501672 | 5.91E-09 | 5.91E-09 | 4.42E-08 |
| KEGG_PATHWAY | hsa05330:Allograft rejection | 9 | 17.64705882 | 5.85E-11 | IL4, CD86, CD80, CD40LG, IFNG, FASLG, CD40, IL10, IL2 | 46 | 37 | 6879 | 36.37544066 | 9.47E-09 | 4.73E-09 | 7.07E-08 |
| KEGG_PATHWAY | hsa05145:Toxoplasmosis | 12 | 23.52941176 | 6.79E-11 | MAPK1, CD40LG, IFNG, IKBKG, TLR2, NFKB1, ALOX5, CD40, NOS2, TGFB1, IL10, STAT3 | 46 | 110 | 6879 | 16.31383399 | 1.10E-08 | 3.66E-09 | 8.21E-08 |
| KEGG_PATHWAY | hsa05161:Hepatitis B | 13 | 25.49019608 | 7.71E-11 | IL6, MMP9, TLR2, FASLG, CXCL8, NFKB1, PTEN, TGFB1, STAT3, MAPK1, CDKN1A, IKBKG, PCNA | 46 | 145 | 6879 | 13.40734633 | 1.25E-08 | 3.12E-09 | 9.33E-08 |
| KEGG_PATHWAY | hsa05321:Inflammatory bowel disease (IBD) | 10 | 19.60784314 | 1.99E-10 | IL4, IL6, IFNG, TLR2, IL13, NFKB1, TGFB1, IL10, STAT3, IL2 | 46 | 64 | 6879 | 23.36616848 | 3.22E-08 | 6.44E-09 | 2.41E-07 |
| KEGG_PATHWAY | hsa04672:Intestinal immune network for IgA production | 9 | 17.64705882 | 4.54E-10 | IL4, IL6, CD86, CD80, CD40LG, CD40, TGFB1, IL10, IL2 | 46 | 47 | 6879 | 28.6359852 | 7.35E-08 | 1.23E-08 | 5.49E-07 |
| KEGG_PATHWAY | hsa05144:Malaria | 9 | 17.64705882 | 6.45E-10 | ICAM1, IL6, CD40LG, IFNG, TLR2, CXCL8, CD40, TGFB1, IL10 | 46 | 49 | 6879 | 27.46716948 | 1.04E-07 | 1.49E-08 | 7.80E-07 |
| KEGG_PATHWAY | hsa05323:Rheumatoid arthritis | 10 | 19.60784314 | 3.68E-09 | CSF2, ICAM1, IL6, CD86, CD80, IFNG, TLR2, CXCL8, TGFB1, MMP1 | 46 | 88 | 6879 | 16.99357708 | 5.96E-07 | 7.45E-08 | 4.45E-06 |
| KEGG_PATHWAY | hsa05320:Autoimmune thyroid disease | 8 | 15.68627451 | 3.38E-08 | IL4, CD86, CD80, CD40LG, FASLG, CD40, IL10, IL2 | 46 | 52 | 6879 | 23.00668896 | 5.48E-06 | 6.09E-07 | 4.10E-05 |
| KEGG_PATHWAY | hsa04066:HIF-1 signaling pathway | 9 | 17.64705882 | 1.51E-07 | MAPK1, CDKN1A, IL6, HMOX1, IFNG, NFKB1, NOS2, STAT3, TIMP1 | 46 | 96 | 6879 | 14.01970109 | 2.45E-05 | 2.45E-06 | 1.83E-04 |
| KEGG_PATHWAY | hsa04660:T cell receptor signaling pathway | 9 | 17.64705882 | 2.08E-07 | IL4, CSF2, MAPK1, CD40LG, IFNG, IKBKG, NFKB1, IL10, IL2 | 46 | 100 | 6879 | 13.45891304 | 3.37E-05 | 3.06E-06 | 2.52E-04 |
| KEGG_PATHWAY | hsa04060:Cytokine-cytokine receptor interaction | 12 | 23.52941176 | 2.99E-07 | IL4, CSF2, IL6, CD40LG, IFNG, CXCL8, FASLG, IL13, CD40, TGFB1, IL10, IL2 | 46 | 243 | 6879 | 7.384863124 | 4.84E-05 | 4.03E-06 | 3.62E-04 |
| KEGG_PATHWAY | hsa05140:Leishmaniasis | 8 | 15.68627451 | 3.07E-07 | IL4, MAPK1, IFNG, TLR2, NFKB1, NOS2, TGFB1, IL10 | 46 | 71 | 6879 | 16.84996938 | 4.97E-05 | 3.82E-06 | 3.71E-04 |
| KEGG_PATHWAY | hsa04620:Toll-like receptor signaling pathway | 9 | 17.64705882 | 3.27E-07 | MAPK1, IL6, CD86, CD80, IKBKG, TLR2, CXCL8, NFKB1, CD40 | 46 | 106 | 6879 | 12.69708778 | 5.30E-05 | 3.79E-06 | 3.96E-04 |
| KEGG_PATHWAY | hsa05146:Amoebiasis | 9 | 17.64705882 | 3.27E-07 | CSF2, IL6, IFNG, TLR2, CXCL8, NFKB1, NOS2, TGFB1, IL10 | 46 | 106 | 6879 | 12.69708778 | 5.30E-05 | 3.79E-06 | 3.96E-04 |
| KEGG_PATHWAY | hsa05200:Pathways in cancer | 14 | 27.45098039 | 7.75E-07 | IL6, MMP9, FASLG, CXCL8, NFKB1, MMP2, PTEN, TGFB1, STAT3, MMP1, MAPK1, CDKN1A, IKBKG, NOS2 | 46 | 393 | 6879 | 5.327248589 | 1.25E-04 | 8.37E-06 | 9.37E-04 |
| KEGG_PATHWAY | hsa05310:Asthma | 6 | 11.76470588 | 1.20E-06 | IL4, CD40LG, MS4A2, IL13, CD40, IL10 | 46 | 30 | 6879 | 29.90869565 | 1.95E-04 | 1.22E-05 | 0.001455586 |
| KEGG_PATHWAY | hsa05162:Measles | 9 | 17.64705882 | 1.87E-06 | IL4, IL6, IFNG, TLR2, FASLG, IL13, NFKB1, STAT3, IL2 | 46 | 133 | 6879 | 10.11948349 | 3.02E-04 | 1.78E-05 | 0.002259182 |
| KEGG_PATHWAY | hsa05332:Graft-versus-host disease | 6 | 11.76470588 | 1.97E-06 | IL6, CD86, CD80, IFNG, FASLG, IL2 | 46 | 33 | 6879 | 27.18972332 | 3.20E-04 | 1.78E-05 | 0.00238911 |
| KEGG_PATHWAY | hsa04068:FoxO signaling pathway | 9 | 17.64705882 | 1.98E-06 | MAPK1, CDKN1A, IL6, FASLG, CAT, PTEN, TGFB1, IL10, STAT3 | 46 | 134 | 6879 | 10.04396496 | 3.20E-04 | 1.68E-05 | 0.002391024 |
| KEGG_PATHWAY | hsa05166:HTLV-I infection | 11 | 21.56862745 | 4.02E-06 | CSF2, ICAM1, CDKN1A, IL6, IKBKG, PCNA, CHEK1, NFKB1, CD40, TGFB1, IL2 | 46 | 254 | 6879 | 6.476292366 | 6.51E-04 | 3.26E-05 | 0.004864675 |
| KEGG_PATHWAY | hsa05219:Bladder cancer | 6 | 11.76470588 | 6.00E-06 | MAPK1, CDKN1A, MMP9, CXCL8, MMP2, MMP1 | 46 | 41 | 6879 | 21.88441145 | 9.71E-04 | 4.63E-05 | 0.007255978 |
| KEGG_PATHWAY | hsa05202:Transcriptional misregulation in cancer | 9 | 17.64705882 | 1.02E-05 | CSF2, CDKN1A, IL6, CD86, MMP9, CXCL8, MPO, NFKB1, CD40 | 46 | 167 | 6879 | 8.059229367 | 0.001655689 | 7.53E-05 | 0.012376558 |
| KEGG_PATHWAY | hsa05132:Salmonella infection | 7 | 13.7254902 | 1.44E-05 | CSF2, MAPK1, IL6, IFNG, CXCL8, NFKB1, NOS2 | 46 | 83 | 6879 | 12.61210058 | 0.002326535 | 1.01E-04 | 0.017396657 |
| KEGG_PATHWAY | hsa04064:NF-kappa B signaling pathway | 7 | 13.7254902 | 1.89E-05 | ICAM1, CD40LG, IKBKG, CXCL8, NFKB1, CD40, BTK | 46 | 87 | 6879 | 12.03223388 | 0.003051327 | 1.27E-04 | 0.022823956 |
| KEGG_PATHWAY | hsa04630:Jak-STAT signaling pathway | 8 | 15.68627451 | 3.71E-05 | IL4, CSF2, IL6, IFNG, IL13, IL10, STAT3, IL2 | 46 | 145 | 6879 | 8.250674663 | 0.005995149 | 2.40E-04 | 0.044905109 |
| KEGG_PATHWAY | hsa05143:African trypanosomiasis | 5 | 9.803921569 | 5.69E-05 | ICAM1, IL6, IFNG, FASLG, IL10 | 46 | 33 | 6879 | 22.65810277 | 0.00918247 | 3.55E-04 | 0.068881052 |
| KEGG_PATHWAY | hsa04668:TNF signaling pathway | 7 | 13.7254902 | 6.12E-05 | CSF2, ICAM1, MAPK1, IL6, MMP9, IKBKG, NFKB1 | 46 | 107 | 6879 | 9.783218204 | 0.009870242 | 3.67E-04 | 0.074064029 |
| KEGG_PATHWAY | hsa05340:Primary immunodeficiency | 5 | 9.803921569 | 6.42E-05 | CD40LG, IKBKG, CD40, ADA, BTK | 46 | 34 | 6879 | 21.99168798 | 0.010351207 | 3.72E-04 | 0.077690505 |
| KEGG_PATHWAY | hsa04664:Fc epsilon RI signaling pathway | 6 | 11.76470588 | 7.32E-05 | IL4, CSF2, MAPK1, MS4A2, IL13, BTK | 46 | 68 | 6879 | 13.19501279 | 0.011782817 | 4.09E-04 | 0.088494523 |
| KEGG_PATHWAY | hsa05133:Pertussis | 6 | 11.76470588 | 1.17E-04 | MAPK1, IL6, CXCL8, NFKB1, NOS2, IL10 | 46 | 75 | 6879 | 11.96347826 | 0.018790831 | 6.32E-04 | 0.14159218 |
| KEGG_PATHWAY | hsa05164:Influenza A | 8 | 15.68627451 | 1.18E-04 | ICAM1, MAPK1, IL6, IFNG, PRSS1, CXCL8, FASLG, NFKB1 | 46 | 174 | 6879 | 6.875562219 | 0.018989286 | 6.18E-04 | 0.143100915 |
| KEGG_PATHWAY | hsa05152:Tuberculosis | 8 | 15.68627451 | 1.32E-04 | MAPK1, IL6, IFNG, TLR2, NFKB1, NOS2, TGFB1, IL10 | 46 | 177 | 6879 | 6.759027266 | 0.021113255 | 6.67E-04 | 0.159265931 |
| KEGG_PATHWAY | hsa04940:Type I diabetes mellitus | 5 | 9.803921569 | 1.49E-04 | CD86, CD80, IFNG, FASLG, IL2 | 46 | 42 | 6879 | 17.80279503 | 0.0238843 | 7.32E-04 | 0.180404551 |
| KEGG_PATHWAY | hsa05205:Proteoglycans in cancer | 8 | 15.68627451 | 2.81E-04 | MAPK1, CDKN1A, MMP9, TLR2, FASLG, MMP2, TGFB1, STAT3 | 46 | 200 | 6879 | 5.98173913 | 0.044463795 | 0.00133683 | 0.339153462 |
| KEGG_PATHWAY | hsa04151:PI3K-Akt signaling pathway | 10 | 19.60784314 | 3.22E-04 | IL4, MAPK1, CDKN1A, IL6, IKBKG, TLR2, FASLG, NFKB1, PTEN, IL2 | 46 | 345 | 6879 | 4.334593573 | 0.050813127 | 0.001488879 | 0.388770686 |
| KEGG_PATHWAY | hsa05134:Legionellosis | 5 | 9.803921569 | 3.98E-04 | IL6, VCP, TLR2, CXCL8, NFKB1 | 46 | 54 | 6879 | 13.84661836 | 0.062477863 | 0.001790476 | 0.480731223 |
| KEGG_PATHWAY | hsa04621:NOD-like receptor signaling pathway | 5 | 9.803921569 | 4.58E-04 | MAPK1, IL6, IKBKG, CXCL8, NFKB1 | 46 | 56 | 6879 | 13.35209627 | 0.071531575 | 0.002003906 | 0.552840092 |
| KEGG_PATHWAY | hsa05416:Viral myocarditis | 5 | 9.803921569 | 4.90E-04 | ICAM1, CD86, CD80, CD40LG, CD40 | 46 | 57 | 6879 | 13.11784897 | 0.076368882 | 0.002088409 | 0.591634235 |
| KEGG_PATHWAY | hsa05212:Pancreatic cancer | 5 | 9.803921569 | 8.09E-04 | MAPK1, IKBKG, NFKB1, TGFB1, STAT3 | 46 | 65 | 6879 | 11.50334448 | 0.122867898 | 0.003355835 | 0.974447797 |
| KEGG_PATHWAY | hsa05169:Epstein-Barr virus infection | 6 | 11.76470588 | 0.001119618 | ICAM1, CDKN1A, IKBKG, NFKB1, CD40, STAT3 | 46 | 122 | 6879 | 7.354597292 | 0.165964867 | 0.004526717 | 1.34640648 |
| KEGG_PATHWAY | hsa05220:Chronic myeloid leukemia | 5 | 9.803921569 | 0.001188942 | MAPK1, CDKN1A, IKBKG, NFKB1, TGFB1 | 46 | 72 | 6879 | 10.38496377 | 0.175289713 | 0.004689534 | 1.42922268 |
| KEGG_PATHWAY | hsa04380:Osteoclast differentiation | 6 | 11.76470588 | 0.001538846 | MAPK1, IFNG, IKBKG, NFKB1, TGFB1, BTK | 46 | 131 | 6879 | 6.849319615 | 0.220798082 | 0.005922513 | 1.846258485 |
| KEGG_PATHWAY | hsa05160:Hepatitis C | 6 | 11.76470588 | 0.001645886 | MAPK1, CDKN1A, IKBKG, CXCL8, NFKB1, STAT3 | 46 | 133 | 6879 | 6.746322328 | 0.234214491 | 0.006186671 | 1.973510793 |
| KEGG_PATHWAY | hsa05322:Systemic lupus erythematosus | 6 | 11.76470588 | 0.001701419 | CD86, CD80, CD40LG, IFNG, CD40, IL10 | 46 | 134 | 6879 | 6.695976639 | 0.241084319 | 0.006250037 | 2.039470634 |
| KEGG_PATHWAY | hsa05215:Prostate cancer | 5 | 9.803921569 | 0.002497686 | MAPK1, CDKN1A, IKBKG, NFKB1, PTEN | 46 | 88 | 6879 | 8.496788538 | 0.333110698 | 0.008962511 | 2.980776466 |
| KEGG_PATHWAY | hsa04932:Non-alcoholic fatty liver disease (NAFLD) | 6 | 11.76470588 | 0.002871796 | IL6, CXCL8, FASLG, NFKB1, ADIPOQ, TGFB1 | 46 | 151 | 6879 | 5.942124964 | 0.372430128 | 0.010077148 | 3.420159438 |
| KEGG_PATHWAY | hsa05221:Acute myeloid leukemia | 4 | 7.843137255 | 0.005693744 | MAPK1, IKBKG, NFKB1, STAT3 | 46 | 56 | 6879 | 10.68167702 | 0.603477471 | 0.019488915 | 6.676107253 |
| KEGG_PATHWAY | hsa05168:Herpes simplex infection | 6 | 11.76470588 | 0.006507868 | IL6, IFNG, IKBKG, TLR2, FASLG, NFKB1 | 46 | 183 | 6879 | 4.903064861 | 0.652753412 | 0.02179482 | 7.596543999 |
| KEGG_PATHWAY | hsa04071:Sphingolipid signaling pathway | 5 | 9.803921569 | 0.007560961 | MAPK1, MS4A2, NFKB1, ABCC1, PTEN | 46 | 120 | 6879 | 6.230978261 | 0.707569963 | 0.024780247 | 8.774802091 |
| KEGG_PATHWAY | hsa04650:Natural killer cell mediated cytotoxicity | 5 | 9.803921569 | 0.008008431 | CSF2, ICAM1, MAPK1, IFNG, FASLG | 46 | 122 | 6879 | 6.128831076 | 0.728172802 | 0.025715355 | 9.271270528 |
| KEGG_PATHWAY | hsa05131:Shigellosis | 4 | 7.843137255 | 0.00825271 | MAPK1, IKBKG, CXCL8, NFKB1 | 46 | 64 | 6879 | 9.346467391 | 0.738804532 | 0.025979822 | 9.541250749 |
| KEGG_PATHWAY | hsa04662:B cell receptor signaling pathway | 4 | 7.843137255 | 0.010145552 | MAPK1, IKBKG, NFKB1, BTK | 46 | 69 | 6879 | 8.669187146 | 0.808328793 | 0.031269403 | 11.60840059 |
| KEGG_PATHWAY | hsa05206:MicroRNAs in cancer | 7 | 13.7254902 | 0.010187895 | CDKN1A, HMOX1, MMP9, NFKB1, ABCC1, PTEN, STAT3 | 46 | 286 | 6879 | 3.660155062 | 0.809652459 | 0.030815294 | 11.6541423 |
| KEGG_PATHWAY | hsa05203:Viral carcinogenesis | 6 | 11.76470588 | 0.010384879 | MAPK1, CDKN1A, IKBKG, CHEK1, NFKB1, STAT3 | 46 | 205 | 6879 | 4.376882291 | 0.815691968 | 0.03083222 | 11.86665682 |
| KEGG_PATHWAY | hsa04920:Adipocytokine signaling pathway | 4 | 7.843137255 | 0.010551932 | IKBKG, NFKB1, ADIPOQ, STAT3 | 46 | 70 | 6879 | 8.545341615 | 0.820664277 | 0.030762284 | 12.04651244 |
| KEGG_PATHWAY | hsa04514:Cell adhesion molecules (CAMs) | 5 | 9.803921569 | 0.013468205 | ICAM1, CD86, CD80, CD40LG, CD40 | 46 | 142 | 6879 | 5.265615432 | 0.888827845 | 0.038466954 | 15.13253826 |
| KEGG_PATHWAY | hsa05222:Small cell lung cancer | 4 | 7.843137255 | 0.017791047 | IKBKG, NFKB1, NOS2, PTEN | 46 | 85 | 6879 | 7.037340153 | 0.945420448 | 0.049739601 | 19.52459542 |
| KEGG_PATHWAY | hsa05020:Prion diseases | 3 | 5.882352941 | 0.020557396 | MAPK1, IL6, SOD1 | 46 | 34 | 6879 | 13.19501279 | 0.965438184 | 0.056366417 | 22.22477124 |
| KEGG_PATHWAY | hsa04062:Chemokine signaling pathway | 5 | 9.803921569 | 0.032565017 | MAPK1, IKBKG, CXCL8, NFKB1, STAT3 | 46 | 186 | 6879 | 4.019985975 | 0.995314784 | 0.086894748 | 33.00899886 |
| KEGG_PATHWAY | hsa04931:Insulin resistance | 4 | 7.843137255 | 0.033157542 | IL6, NFKB1, PTEN, STAT3 | 46 | 108 | 6879 | 5.538647343 | 0.995757463 | 0.087021759 | 33.50379797 |
| KEGG_PATHWAY | hsa04110:Cell cycle | 4 | 7.843137255 | 0.046884693 | CDKN1A, PCNA, CHEK1, TGFB1 | 46 | 124 | 6879 | 4.82398317 | 0.999581629 | 0.119730267 | 44.06939933 |
